# ArchR is a scalable software package for integrative single-cell chromatin accessibility analysis

**DOI:** 10.1038/s41588-021-00790-6

**Published:** 2021-02-25

**Authors:** Jeffrey M. Granja, M. Ryan Corces, Sarah E. Pierce, S. Tansu Bagdatli, Hani Choudhry, Howard Y. Chang, William J. Greenleaf

**Affiliations:** 1 Department of Genetics, Stanford University School of Medicine, Stanford, CA USA; 2 Program in Biophysics, Stanford University, Stanford, CA USA; 3 Center for Personal Dynamic Regulomes, Stanford University, Stanford, CA USA; 4 Department of Pathology, Stanford University School of Medicine, Stanford, CA USA; 5 Gladstone Institute of Neurological Disease, Gladstone Institute of Data Science and Biotechnology, San Francisco, CA USA; 6 Department of Neurology, University of California San Francisco, San Francisco, CA USA; 7 Program in Cancer Biology, Stanford University School of Medicine, Stanford, CA USA; 8 Department of Biochemistry, Faculty of Science, Cancer and Mutagenesis Unit, King Fahd Center for Medical Research, King Abdulaziz University, Jeddah, Saudi Arabia; 9 Howard Hughes Medical Institute, Stanford University, Stanford, CA USA; 10 Department of Applied Physics, Stanford University, Stanford, CA USA; 11 Chan Zuckerberg Biohub, San Francisco, CA USA

**Keywords:** Epigenetics, Gene regulation, Epigenomics

## Abstract

The advent of single-cell chromatin accessibility profiling has accelerated the ability to map gene regulatory landscapes but has outpaced the development of scalable software to rapidly extract biological meaning from these data. Here we present a software suite for single-cell analysis of regulatory chromatin in R (ArchR; https://www.archrproject.com/) that enables fast and comprehensive analysis of single-cell chromatin accessibility data. ArchR provides an intuitive, user-focused interface for complex single-cell analyses, including doublet removal, single-cell clustering and cell type identification, unified peak set generation, cellular trajectory identification, DNA element-to-gene linkage, transcription factor footprinting, mRNA expression level prediction from chromatin accessibility and multi-omic integration with single-cell RNA sequencing (scRNA-seq). Enabling the analysis of over 1.2 million single cells within 8 h on a standard Unix laptop, ArchR is a comprehensive software suite for end-to-end analysis of single-cell chromatin accessibility that will accelerate the understanding of gene regulation at the resolution of individual cells.

## Abstract (web summary)

ArchR is a software suite that enables efficient and end-to-end analysis of single-cell chromatin accessibility data (scATAC-seq).

## Main

Single-cell approaches have revolutionized the understanding of biology, from interrogation of cellular heterogeneity to identification of disease-specific processes. The advent of single-cell approaches for the assay for transposase-accessible chromatin using sequencing (scATAC-seq) has made it possible to study chromatin accessibility and gene regulation in single cells^1,2^, illuminating cell-type-specific biology^3–7^. Recent advances increased the throughput of scATAC-seq, enabling a laboratory to generate data from hundreds of thousands of cells on the timescale of weeks^5,6,8^. These advances were driven by an increased interest in chromatin-based gene regulation across a diversity of cellular contexts and biological systems^1,2,5,6,8,9^. This capacity for data generation outpaced the development of intuitive, benchmarked and comprehensive software for scATAC-seq analysis^10^, a crucial requirement that would facilitate the broad use of these methods for investigating gene regulation at cellular resolution.

**Fig. 1:**
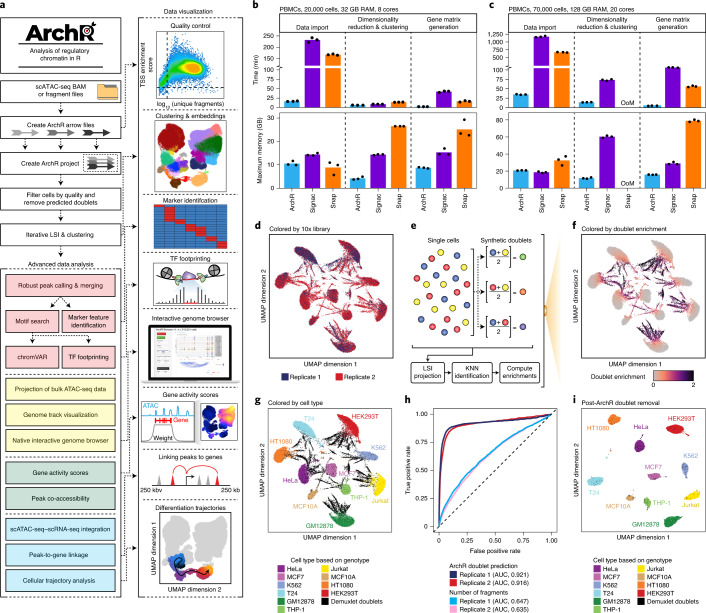
ArchR, a rapid, extensible and comprehensive scATAC-seq analysis platform. **a**, Schematic of the ArchR workflow from pre-aligned scATAC-seq data as BAM or fragment files to diverse data analysis. **b**,**c**, Comparison of runtime and memory usage by ArchR, Signac and SnapATAC (Snap) for the analysis of ~20,000 PBMCs using 32 GB of RAM and eight cores (**b**) or ~70,000 PBMCs using 128 GB of RAM and 20 cores (**c**). Dots represent replicates of benchmarking analysis (*n* = 3). OoM corresponds to out of memory. **d**, Initial UMAP embedding of scATAC-seq data from two replicates of the cell line-mixing experiment (*n* = 38,072 total cells from ten different cell lines), colored by replicate number. **e**, Schematic of doublet identification with ArchR. KNN, *k*-nearest neighbors. **f**,**g**, Initial UMAP embedding of scATAC-seq data from two replicates of the cell line-mixing experiment (*n* = 38,072 total cells from ten different cell lines), colored by the enrichment of projected synthetic doublets (**f**) or the demuxlet identification labels based on genotype identification using single-nucleotide polymorphisms (SNPs) within accessible chromatin sites (**g**). **h**, ROC curves of doublet prediction using ArchR doublet identification or the number of fragments per cell compared to demuxlet as a ground truth. The AUCs for these ROC curves are annotated below. **i**, UMAP after ArchR doublet removal of scATAC-seq data from two replicates of the cell line-mixing experiment (*n* = 27,220 doublet-filtered cells from ten different cell lines), colored by demuxlet identification labels based on genotype identification using SNPs within accessible chromatin sites.

**Extended Data Fig. 1:**
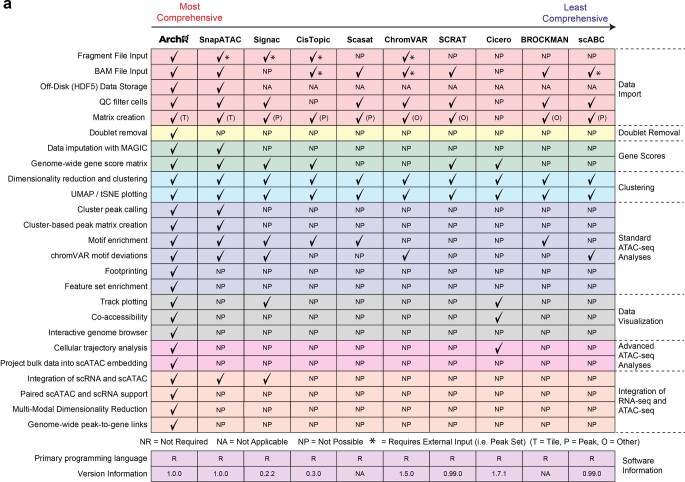
Comparison of supported features from currently available scATAC-seq software. **a**, Comparison of comprehensiveness of supported scATAC-seq features across ArchR and other existing software packages.

To this end, we sought to develop a software suite for both routine and advanced analysis of massive-scale single-cell chromatin accessibility data without the need for high-performance computing environments. This package for single-cell Analysis of Regulatory Chromatin in R (ArchR; https://www.archrproject.com/) provides a facile platform to interrogate scATAC-seq data from multiple scATAC-seq implementations, including the 10x Genomics Chromium system^6,7^, the Bio-Rad droplet scATAC-seq system^8^, single-cell combinatorial indexing^2,5^ and the Fluidigm C1 system^1,4^ (Fig. 1a). ArchR provides a user-focused interface for complex scATAC-seq analysis, such as marker feature identification, transcription factor (TF) footprinting, interactive sequencing track visualization, scRNA-seq integration and cellular trajectory identification (Fig. 1a). When compared to other existing tools, such as SnapATAC^11^ and Signac^12^, ArchR provides a more extensive set of features (Extended Data Fig. 1a) and is designed to provide the speed and flexibility to support interactive analysis, enabling iterative extraction of meaningful biological interpretations^11–19^.

## Results

### The ArchR framework

ArchR takes as input aligned BAM or fragment files, which are first parsed in small chunks per chromosome, read in parallel to conserve memory and then efficiently stored on disk using the compressed random-access hierarchical data format version 5 (HDF5) file format (Supplementary Fig. 1a). These HDF5 files form the constituent pieces of an ArchR analysis that we call ‘Arrow’ files. Arrow files are grouped into an ‘ArchR Project’, a compressed R data file that is stored in memory, which provides an organized, rapid and low memory-use framework for manipulation of the larger Arrow files stored on disk (Supplementary Fig. 1b). Arrow files are always accessed in minimal chunks using efficient parallel read and write operations that reduce runtime and memory usage (Supplementary Fig. 1c,d). Moreover, the base file size of Arrow files remains smaller than the input fragment files across various cellular inputs (Supplementary Fig. 2a,b). Throughout this report, we compare ArchR to SnapATAC and Signac, as these are two commonly used scATAC-seq analysis packages with the most comparable set of features, and many of the other existing software are not suited for analyzing datasets larger than 80,000 cells^10^. However, we note that these comparisons use specific versions of software (Extended Data Fig. 1a) that are still in active development and are likely to change over time.

### ArchR enables efficient and comprehensive single-cell chromatin accessibility analysis

To benchmark the performance of ArchR, we collected three diverse publicly available datasets (Supplementary Table 1): (1) peripheral blood mononuclear cells (PBMCs), which represent discrete primary cell types^6,7^ (Supplementary Fig. 2c–f), (2) bone marrow stem and progenitor cells and differentiated cells, which represent a continuous cellular hierarchy^7^ (Supplementary Fig. 2g–j), and (3) a large atlas of murine cell types from diverse organ systems^5^ (Supplementary Fig. 2k–m). Before downstream analysis, we removed cells generating low-quality data. To assess per-cell data quality, ArchR computes transcription start site (TSS) enrichment scores, which have become the standard for bulk ATAC-seq analysis^20^ and provide clearer separation of cells generating low- and high-quality data compared to that from the fraction of reads in promoters^11^ (Supplementary Fig. 2d,h).

**Extended Data Fig. 2:**
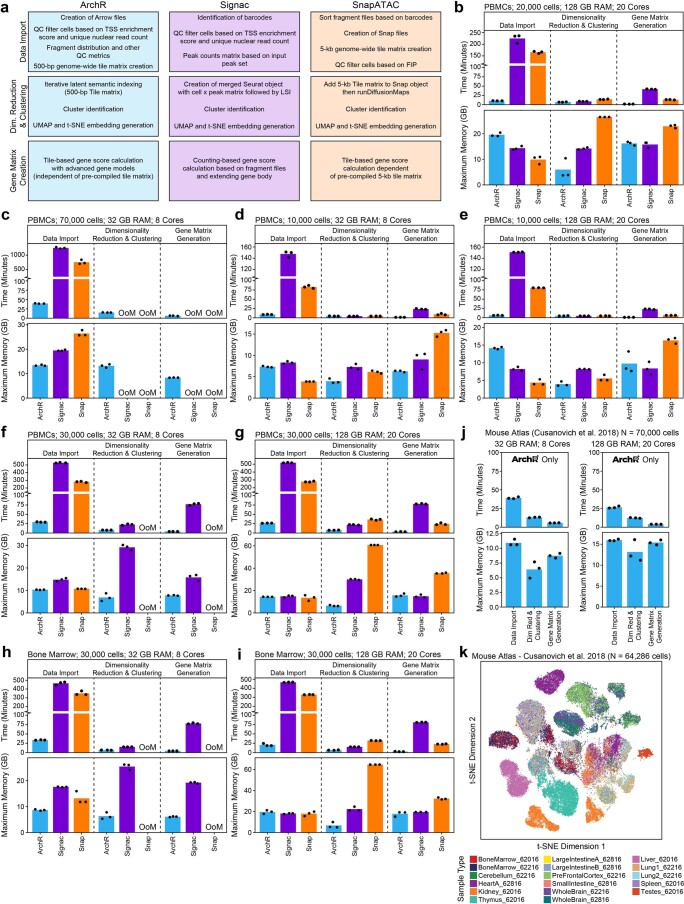
Benchmarking comparisons of runtime and memory usage for ArchR, Signac, and SnapATAC. **a**, Schematic describing the individual benchmarking steps compared across ArchR (blue), Signac (purple), and SnapATAC (orange) for (1) Data Import, (2) Dimensionality Reduction and Clustering, and (3) Gene Score Matrix Creation. **b-i**, Comparison of ArchR, Signac, and SnapATAC for run time and peak memory usage for the analysis of (**b**) ~20,000 cells from the PBMCs dataset using 128 GB of RAM and 20 cores (plot corresponds to Fig. 1b), (**c**) ~70,000 cells from the PBMCs dataset using 32 GB of RAM and 8 cores (plot corresponds to Fig. 1c), (**d-e**) ~10,000 cells from the PBMCs dataset using (**d**) 32 GB of RAM and 8 cores or (**e**) 128 GB of RAM and 20 cores, (**f-g**) ~30,000 cells from the PBMCs dataset using (**f**) 32 GB of RAM and 8 cores or (**g**) 128 GB of RAM and 20 cores, and (**h-i**) ~30,000 cells from the bone marrow dataset using (**h**) 32 GB of RAM and 8 cores or (**i**) 128 GB of RAM and 20 cores. Dots represent individual replicates of benchmarking analysis (N = 3). OoM corresponds to out of memory. **j**, Benchmarks from ArchR for run time and peak memory usage for the analysis of ~70,000 cells from the sci-ATAC-seq mouse atlas dataset (N = 13 tissues) for (left) 32 GB of RAM with 8 cores and (right) 128 GB of RAM with 20 cores. Dots represent individual replicates of benchmarking analysis. **k**, t-SNE of mouse atlas scATAC-seq data (N = 64,286 cells) colored by individual samples.

To quantify the ability of ArchR to analyze large-scale data, we benchmarked ArchR for three of the major scATAC-seq analytical steps across these three datasets using two different computational infrastructures (Extended Data Fig. 2a and Supplementary Table 2). We observed that ArchR outperforms SnapATAC and Signac in speed and memory usage across all comparisons, enabling analysis of 70,000-cell datasets in under an hour with 32 GB of random-access memory (RAM) and eight cores (Fig. 1b,c and Extended Data Fig. 2b–i). Additionally, when analyzing a 70,000-cell dataset, SnapATAC exceeded the available memory in the high-memory setting (128 GB RAM, 20 cores) (Fig. 1c), and both SnapATAC and Signac exceeded the available memory in the low-memory setting (32 GB RAM, eight cores) (Extended Data Fig. 2c), while ArchR completed these analyses faster and without exceeding the available memory. In addition to using fragment files as input, ArchR can directly convert BAM files to Arrow files, enabling the analysis of scATAC-seq data from diverse single-cell platforms, including single-cell combinatorial indexing (sci)-ATAC-seq^5^ (Extended Data Fig. 2j,k).

### ArchR identifies putative doublets in scATAC-seq data

**Extended Data Fig. 3:**
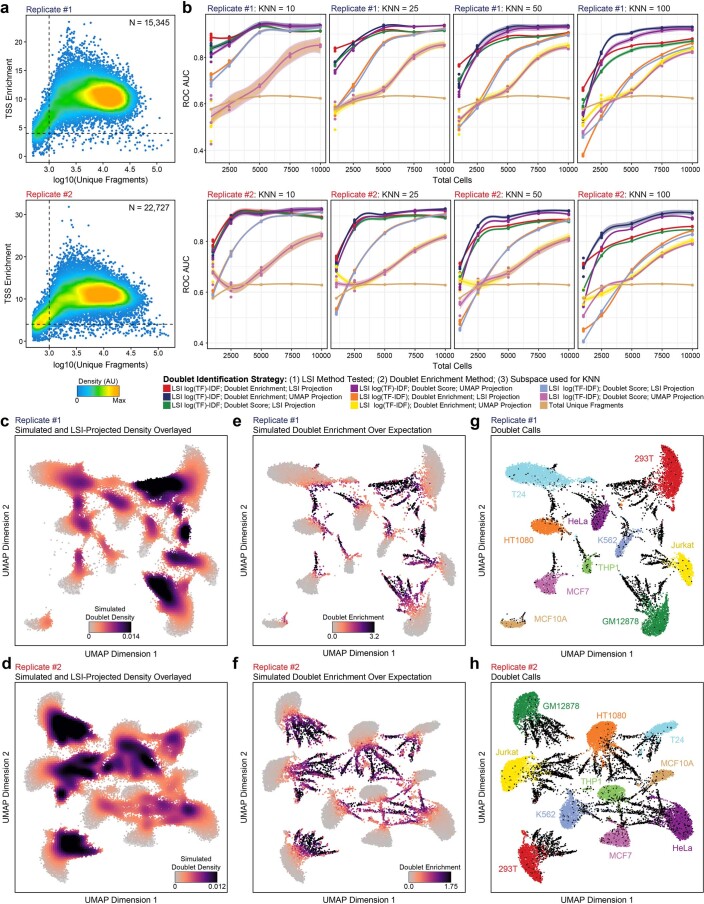
Optimization of doublet identification using admixtures of cell lines. **a**, QC filtering plots from ArchR for (top) replicate 1 and (bottom) replicate 2 from the cell line mixing dataset showing the TSS enrichment score vs unique nuclear fragments per cell. Dot color represents the density in arbitrary units of points in the plot. **b**, Accuracy of various doublet prediction methods for (top) replicate 1 and (bottom) replicate 2 from the cell line mixing dataset, measured by the area under the curve (AUC) of the receiver operating characteristic (ROC), across different in silico cell loadings. Accuracy is determined with respect to genotype-based identification of doublets using demuxlet. Above each plot, ‘KNN’ represents the number of cells nearby each projected synthetic doublet to record when calculating doublet enrichment scores. The distance for KNN recording is determined in the LSI subspace for LSI projection and in the UMAP embedding for UMAP projection parameters. The smooth line represents a LOESS fit (shading represents 95% confidence interval). **c-h**, UMAP of scATAC-seq data showing the (**c-d**) simulated doublet density, (**e-f**) simulated doublet enrichment, or (**g-h**) cell line identity based on genotyping information and demuxlet for (**c,e,g**) replicate 1 (N = 15,345 cells) and (**d,f,h**) replicate 2 (N = 22,727 cells) of the cell line mixing dataset.

**Extended Data Fig. 4:**
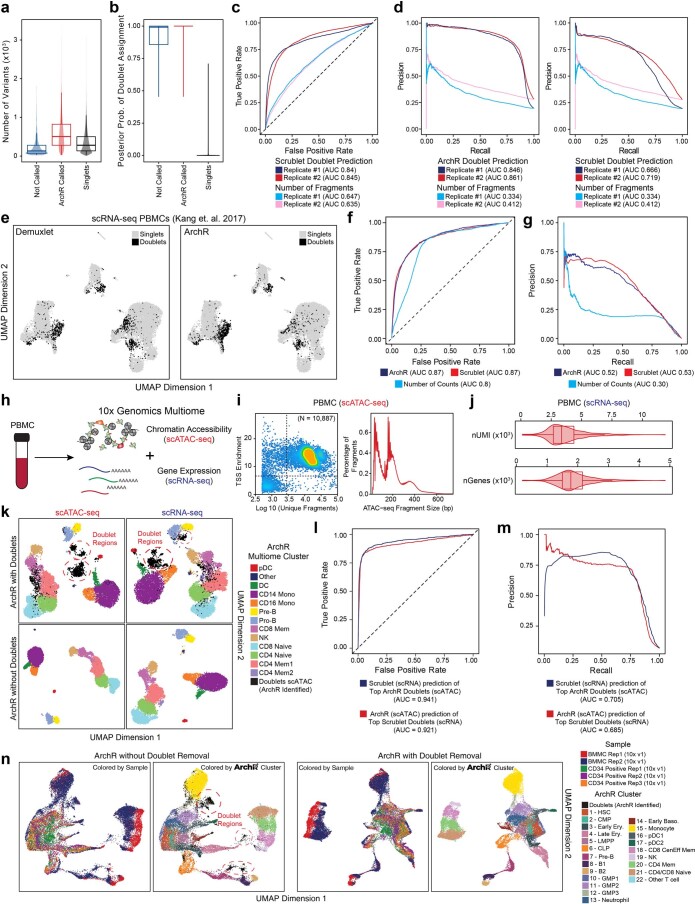
Benchmarking of doublet identification in ArchR. **a**, Total number of sample-relevant single-nucleotide variants that overlapped scATAC-seq fragments for (blue) cells identified as doublets by demuxlet and not identified by ArchR, (red) cells identified as doublets by both demuxlet and ArchR, and (black) cells identified as singlets by demuxlet. Box-whisker plot; lower whisker is the lowest value greater than the 25% quantile minus 1.5 times the interquartile range (IQR), the lower hinge is the 25% quantile, the middle is the median, the upper hinge is the 75% quantile and the upper whisker is the largest value less than the 75% quantile plus 1.5 times the IQR. **b**, Posterior probability of demuxlet doublet assignment. Coloration and box-whisker plot as in (**a**). **c**, Receiver operating characteristic (ROC) curves of doublet prediction using Scrublet or the number of nuclear fragments per cell compared to demuxlet as a ground truth. The area under the curve (AUC) for these ROC curves is annotated below the plot. The Scrublet ROC curve can be directly compared to ArchR in Fig. 1h. **d**, Precision recall (PR) curves of doublet prediction using (left) ArchR and (right) Scrublet doublet identification or the number of nuclear fragments per cell compared to demuxlet as a ground truth. The AUC is annotated below the plot. **e**, UMAP of PBMC mixing scRNA-seq from Kang et. al, 2017. Cells are colored as doublets (black) or singlets (gray) as identified by (left) demuxlet or (right) ArchR. **f-g.** **(f)** ROC curves or (**g**) PR curves of doublet prediction using ArchR (dark blue), Scrublet (red), or the number of total counts per cell (light blue) compared to demuxlet as a ground truth. The AUC is annotated below the plot. **h**, Schematic of 10x Genomics Multiome workflow. **i**, (left) TSS enrichment score vs unique nuclear fragments per cell (color is density), or (right) aggregate fragment size distributions for the cells passing ArchR QC thresholds from the PBMC Multiome data. **j**, Distribution of (top) the number of unique molecular identifiers (nUMIs) per cell passing scATAC-seq filtration and (bottom) the number of unique genes (nGenes) identified with at least 1 UMI per cell. Box-whisker plot as in (**a**). **k**, UMAPs of (left) scATAC-seq data or (right) scRNA-seq data from the Multiome dataset shown (top) with doublets present (black, N = 10,887) and (bottom) with ArchR-identified doublets removed (N = 9,702). **l-m**, (**l**) ROC and (**m**) PR curves of doublet prediction using ArchR (red) compared to the top doublets (N = 750) identified by Scrublet as a ground truth and vice versa (blue). The AUC is annotated below the plot. **n**, UMAP of scATAC-seq data from CD34 + bone marrow cells (green, purple, orange) and unfractionated bone marrow cells (blue and red) colored by (left) sample and (right) ArchR identified clusters (N ~ 30,000 cells total). Plots are shown (left pair) without doublet removal and (right pair) with ArchR-based doublet removal.

The presence of ‘doublets’ (two cells that are captured in the same droplet or nanoreaction) often complicates single-cell analysis. Doublets appear as a superposition of signals from both cells, leading to the false appearance of distinct clusters or false connections between distinct cell types. To mitigate this issue, we designed a doublet detection-and-removal algorithm as part of ArchR. Similarly to methods employed for doublet detection in scRNA-seq^21,22^, ArchR identifies heterotypic doublets by bioinformatically generating a collection of synthetic doublets, projecting these synthetic doublets into the low-dimensional data embedding and then identifying the nearest neighbors to these synthetic doublets as doublets themselves^21,22^ (Fig. 1d–f). To validate this approach, we carried out scATAC-seq on a mixture of ten human cell lines (*n* = 38,072 cells), allowing for genotype-based identification of doublets via demuxlet^23^ as a ground-truth comparison for computational identification of doublets by ArchR (Fig. 1g and Extended Data Fig. 3a). Optimization of doublet prediction parameters (Extended Data Fig. 3b) led to accurate doublet predictions (receiver operating characteristic (ROC) area under the curve (AUC) = 0.918), significantly outperforming doublet prediction based on the total number of fragments (ROC AUC = 0.641) (Fig. 1h and Extended Data Fig. 3c–h). With these predicted doublets excluded, the remaining cells formed ten large groups according to their cell line of origin (Fig. 1i). We note there were some predicted doublets identified by demuxlet that were not identified by ArchR, residing within cluster boundaries and not in intermediate zones (Fig. 1i). We predict that these are imbalanced doublets with the majority of fragments in the droplet, and thus the majority of the scATAC-seq signal coming from a single cell. This hypothesis is further supported by a lower predicted doublet probability in demuxlet for these undetected putative doublets (Extended Data Fig. 4a,b).

To benchmark the performance of doublet identification in ArchR, we compared it to doublet identification with Scrublet^22^, a tool designed for detecting doublets in scRNA-seq data. Using our cell line-mixing scATAC-seq data, ArchR shows a modest performance improvement over Scrublet (Extended Data Fig. 4c,d), likely attributable to the fact that Scrublet was not designed specifically for scATAC-seq data. Consistent with this result, ArchR and Scrublet performed comparably in identification of doublets from scRNA-seq cell-mixing data (Extended Data Fig. 4e–g)^23^. To further benchmark doublet identification in ArchR, we used data from PBMCs generated using the 10x Genomics Multiome platform, which collects both scATAC-seq and scRNA-seq data from the same single cells. By comparing doublets identified in scATAC-seq space by ArchR to doublets identified in scRNA-seq space with Scrublet, we found that the high-confidence doublet calls in ArchR were highly concordant (AUC = 0.921) with doublet calls from Scrublet (Extended Data Fig. 4h–m). Last, doublet identification in ArchR for continuous cellular trajectories, such as hematopoietic differentiation, does not exclusively identify doublets along the biologically relevant continuous branches of differentiation (Extended Data Fig. 4n). The majority of predicted doublets reside in spurious clusters, which, if not removed, can be misinterpreted as bonafide cell types. This result indicates that true biological intermediate cell types are not confounded with synthetic cellular mixtures in our doublet identification, consistent with the performance of similar projection-based doublet identification in scRNA-seq data^22^. In summary, the identification and removal of heterotypic doublets in ArchR reduces false cluster identification and improves the fidelity of downstream results.

### ArchR provides high-resolution and efficient dimensionality reduction of scATAC-seq data

ArchR additionally provides methodological improvements over other available software. One of the fundamental aspects of ATAC-seq analysis is the identification of a peak set for downstream analysis. In the context of scATAC-seq, identification of peak regions before cluster identification requires peak calling from all cells as a single group. This obscures cell-type-specific chromatin accessibility, which distorts downstream analyses. For Signac, a counts matrix is created using a predetermined peak set, preventing the contribution of peaks that are specific to lowly represented cell types. Instead of using a predetermined peak set, SnapATAC creates a genome-wide tile matrix of 5-kb bins by default, allowing for unbiased genome-wide identification of cell-type-specific chromatin accessibility. However, 5-kb bins are substantially larger than the average regulatory element (~300–500 bp, containing TF-binding sites less than 50 bp)^24–26^, thus causing multiple regulatory elements to be grouped together, again obscuring cell-type-specific biology. To avoid both of these pitfalls, ArchR operates efficiently on a genome-wide tile matrix of 500-bp bins, allowing for the sensitivity to capture cell-type-specific biology at regulatory elements across the genome. Despite this 500-bp tile matrix, with tenfold higher resolution than SnapATAC, ArchR stores both per-tile accessibility information and all ATAC-seq fragments in an Arrow file that is smaller than either the original input fragments file or the SnapATAC file containing the genome-wide tile matrix at a resolution of only 5-kb (Supplementary Fig. 2a,b). We note that, while SnapATAC has the ability to use a genome-wide 500-bp tile matrix, downstream computation using this high-resolution matrix exceeds the memory limits of common computational infrastructure (Supplementary Fig. 3a,b).

**Extended Data Fig. 5:**
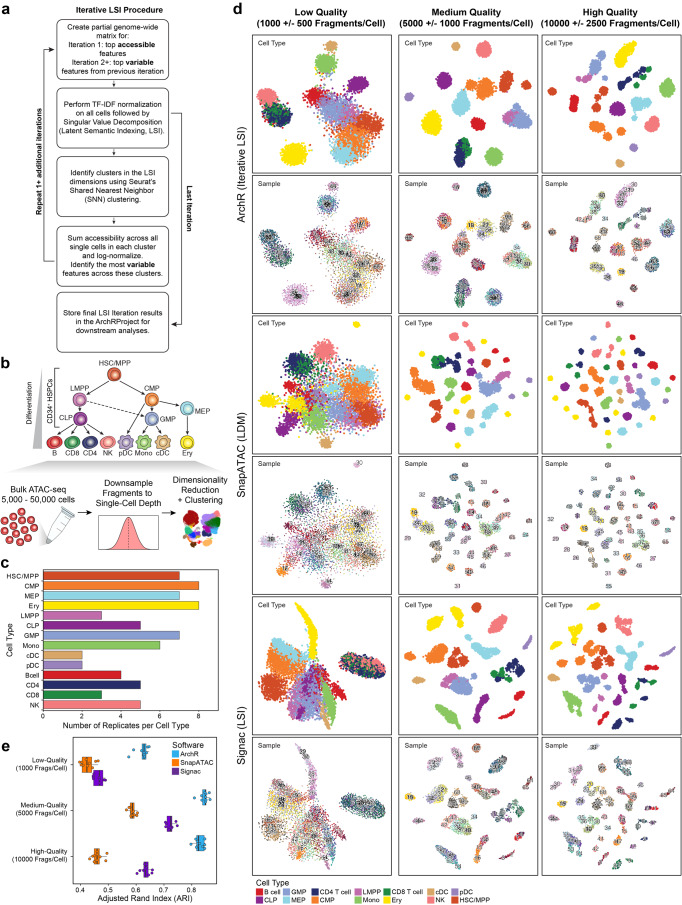
Benchmarking of performance of clustering approaches in ArchR, SnapATAC, and Signac. **a**, Schematic of the iterative LSI procedure implemented in ArchR for dimensionality reduction. **b**, Schematic of the downsampling approach used on bulk ATAC-seq data to enable evaluation of clustering performance for simulated scATAC-seq data. **c**, Bar plot showing the number of replicates generated per cell type by downsampling of bulk ATAC-seq data from hematopoietic cells. **d**, *t*-distributed stochastic neighbor embedding (t-SNE) of downsampled bulk ATAC-seq data from hematopoeitc cells (N = 7,200) to various data quality scales. Left; low-quality scATAC-seq data (~1,000 fragments/cell). Middle; medium-quality scATAC-seq data (~5,000 fragments/cell). Right; high-quality scATAC-seq data (~10,000 fragments/cell). t-SNE plots were created for (top) ArchR (iterative LSI), (middle) SnapATAC (LDM), and (bottom) Signac (LSI). Within each group, these t-SNE plots are colored by (top) cell type and (bottom) sample replicate. **e**, Adjusted Rand Index (ARI) of clusters identified by ArchR (blue), SnapATAC (orange), and Signac (purple) for low-quality, medium-quality and high-quality downsampling of bulk ATAC-seq data from hematopoietic cells.

One major application of single-cell analysis is the identification of cellular subsets through dimensionality reduction and clustering. To benchmark the performance of dimensionality reduction and clustering in ArchR, we compared ArchR to the two best-performing methods identified in previous assessments of scATAC-seq analysis tools^10^, latent semantic indexing (LSI), implemented by Signac, and landmark diffusion maps (LDM), implemented by SnapATAC. For dimensionality reduction, ArchR uses an optimized iterative LSI method^6,7^ (Extended Data Fig. 5a) that exhibits less susceptibility to batch effects by focusing on the most variable features through multiple iterations of LSI. We directly compared the results from these different dimensionality reduction methods using bulk hematopoietic ATAC-seq data downsampled to match single-cell depth (Extended Data Fig. 5b). We performed this downsampling across multiple biological samples for each cell type (14 cell types with, on average, five biological replicates), allowing biological and technical variability to contribute to clustering (Extended Data Fig. 5c). We additionally downsampled these data across multiple quality scales, simulating low-quality scATAC-seq data (1,000 ± 500 fragments per cell), medium-quality scATAC-seq data (5,000 ± 1,000 fragments per cell) and high-quality scATAC-seq data (10,000 ± 2,500 fragments per cell) (Extended Data Fig. 5d). In all cases, ArchR outperformed both SnapATAC and Signac, as assessed by a higher adjusted Rand index (Extended Data Fig. 5e). This was due to overclustering by SnapATAC and Signac, which group downsampled cells first based on biological sample rather than on cell type (Extended Data Fig. 5d). To illustrate these performance differences using real-world data, we compared these dimensionality reduction methods using scATAC-seq data derived from PBMCs (Supplementary Fig. 4) and scATAC-seq data derived from bone marrow cells (Supplementary Fig. 5). In both cases, ArchR identified clusters similar to those in other methods while being less biased by low-quality cells and doublets (Supplementary Figs. 4 and 5). However, when comparing clustering of the bone marrow cell dataset, we found that ArchR alone maintained the structure of the continuous differentiation trajectories from immature CD34^+^ hematopoietic stem and progenitor cells through differentiated myeloid, erythroid and B cells (Supplementary Fig. 5)^4,6–8,12,27^. Notably, the T cell differentiation trajectory, which involves maturation in the thymus, is not captured in the bone marrow.

To enable the efficient examination of massive datasets, ArchR implements a new estimated LSI dimensionality reduction by first creating an iterative LSI reduction from a subset of the total cells and then linearly projecting the remaining cells into this subspace using LSI projection^7^ (Supplementary Fig. 6a). We compared this approach to the LDM estimation method used by SnapATAC, which uses a non-linear reduction based on a subset of cells and then projects the remaining cells into this subspace using LDM projection. When comparing ‘landmark’ subsets of different cell numbers, the estimated LSI approach implemented by ArchR was more consistent and could recapitulate the clusters called and the overall structure of the data with as few as 50 cells across both the PBMC (*n* = 27,845 cells) and bone marrow cell (*n* = 26,748 cells) datasets (Supplementary Figs. 6b and 7a,b). We hypothesize that the observed differences stem from (1) the stability of using a feature matrix versus a Jaccard distance matrix and (2) the linearity of the LSI projection (based on singular value decomposition dimensionality reduction)^28,29^, as compared to the non-linear LDM projection (based on diffusion maps)^30,31^. The estimated LSI approach implemented by ArchR was also faster than the estimated LDM approach implemented by SnapATAC (Supplementary Fig. 7c). Furthermore, the efficiency of the iterative LSI implementation in ArchR limits the requirement for this estimated LSI approach to only extremely large datasets (>200,000 cells for 32 GB of RAM and eight cores), whereas estimated LDM approaches are required for comparatively smaller datasets (>25,000 cells for 32 GB of RAM and eight cores) in SnapATAC. ArchR therefore has the ability to efficiently analyze both large- and small-scale datasets.

### Improved inference of gene scores enables accurate cluster identification with ArchR

**Fig. 2:**
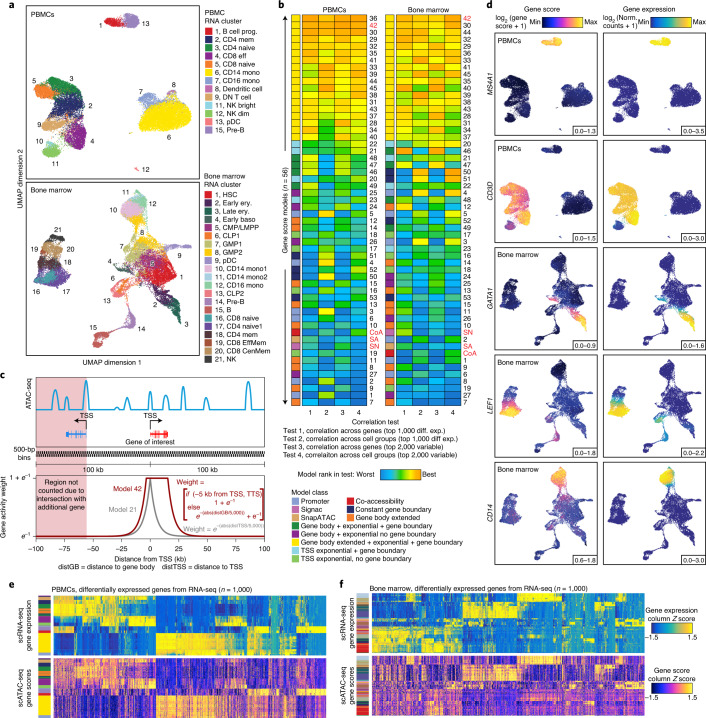
Optimized gene score inference models improve prediction of gene expression from scATAC-seq data. **a**, UMAPs of scATAC-seq from PBMCs (top) and bone marrow cells (bottom), colored by aligned scRNA-seq clusters. This alignment was used for benchmarking of scATAC-seq gene score models. A list of abbreviations used in this figure appear in the Methods. **b**, Heatmaps summarizing the accuracy (measured by Pearson correlation) across 56 gene score models for both the top 1,000 differentially expressed (diff. exp.) and the top 2,000 variable genes for both PBMC and bone marrow cell datasets. Each heatmap entry is colored by the model rank in the given correlation test as described below the heatmap. The model class is indicated to the left of each heatmap by color. SA, SnapATAC; SN, Signac; CoA, co-accessibility. **c**, Illustration of the gene score model 42, which uses bi-directional exponential decays from the gene TSS (extended upstream by 5 kb) and the gene transcription termination site (TTS) while accounting for neighboring gene boundaries. This model was shown to be more accurate than other models, such as model 21 (exponential decay). **d**, Side-by-side UMAPs for PBMCs and bone marrow cells colored by gene scores from model 42 (left) and gene expression from the scRNA-seq alignment for key immune cell-related marker genes (right). Norm., normalized. **e**,**f**, Heatmaps of gene expression (top) or gene scores for the top 1,000 differentially expressed genes (bottom) (selected from scRNA-seq) across all cell aggregates for PBMCs (**e**) or bone marrow cells (**f**). Color bars to the left of each heatmap represent the PBMC or bone marrow cell cluster derived from scRNA-seq data.

**Extended Data Fig. 6:**
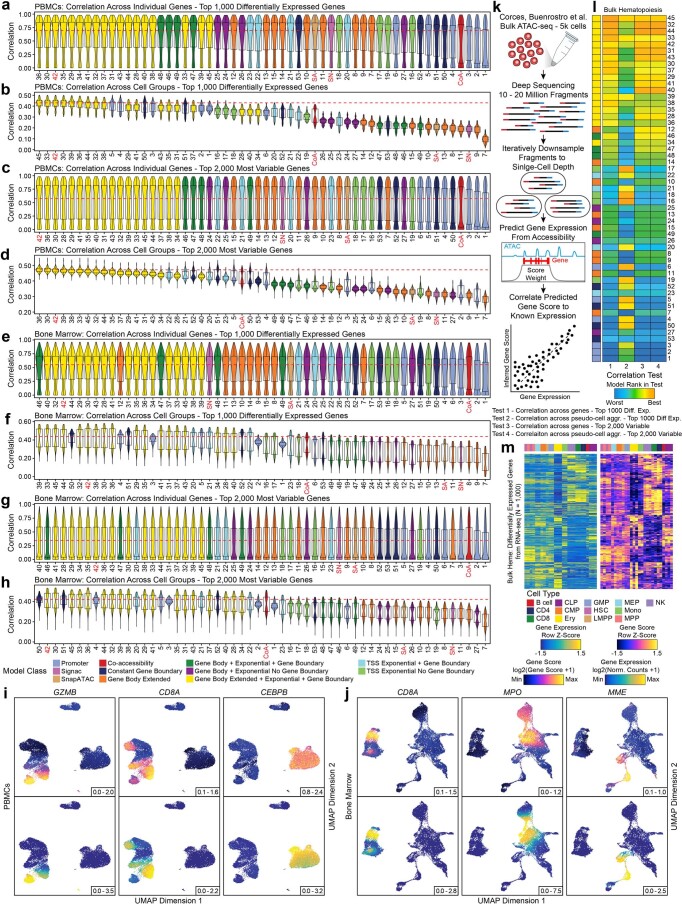
Assessment of gene score models. **a-h**, Distribution of Pearson correlations of inferred gene score and aligned gene expression for (**a,c,e,g**) each gene or (**b,d,f,h**) each cell group across groups of 100 cells (N = 500 groups). Distributions are either presented for (**a,b,e,f**) the top 1,000 differentially expressed genes or (**c,d,g,h**) the top 2,000 most variable genes for each of the 56 gene score models. The red dotted line represents the median value of the best-performing model. Violin plots represent the smoothed density of the distribution of the data. In box plots, the lower whisker is the lowest value greater than the 25% quantile minus 1.5 times the interquartile range, the lower hinge is the 25% quantile, the middle is the median, the upper hinge is the 75% quantile and the upper whisker is the largest value less than the 75% quantile plus 1.5 times the interquartile range. SA, SnapATAC; SN, Signac; CoA, Co-accessibility. **i-j**, UMAPs of scATAC-seq data from (**i**) cells from the PBMCs dataset (N = 27,845 cells) or (j) cells from the bone marrow cell dataset (N = 26,748 cells) colored by (top) inferred gene scores or (bottom) gene expression for several marker genes. **k**, Schematic illustrating the methodology used to assess the accuracy of inferred gene scores. **l**, Heatmaps summarizing the accuracy (Pearson correlation) across all models for both the top 1,000 differentially expressed and top 2,000 variable genes for bulk ATAC-seq and RNA-seq from hematopoietic cell types. Each entry is colored by the model rank in the given test as described below the heatmap. The model class is indicated to the left. SA, SnapATAC; SN, Signac; CoA, Co-accessibility. **m**, Heatmaps of (left) gene expression or (right) gene scores for the top 1,000 differentially expressed genes (selected from bulk RNA-seq) across all cell types from the matched bulk ATAC-seq and RNA-seq data.

**Extended Data Fig. 7:**
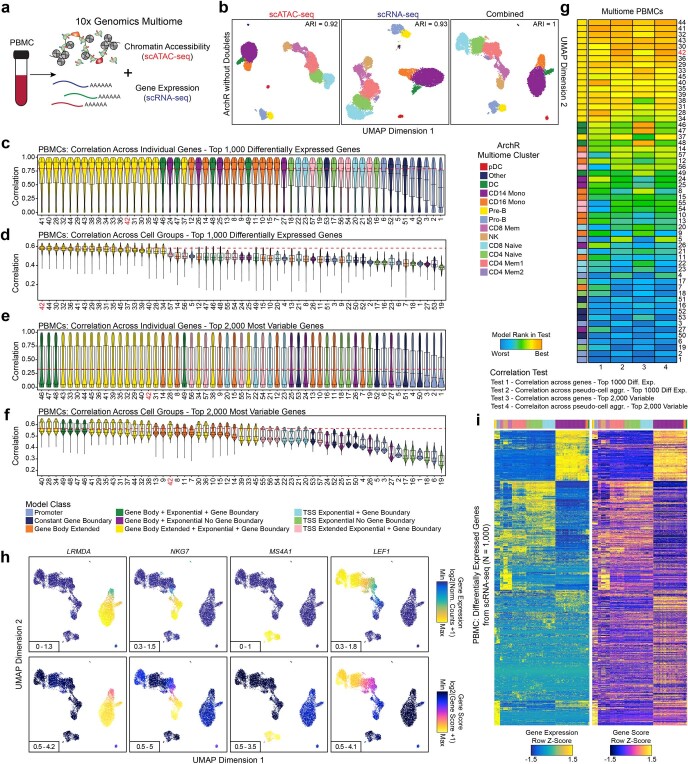
Confirmation of gene score model performance using paired scATAC-seq and scRNA-seq data from the same single cells. **a**, Schematic of 10x Genomics Multiome profiling of scATAC-seq and scRNA-seq in the same single cells. **b**, UMAPs of Multiome data from PBMCs (N = 9,702 cells) with removal of ArchR-identified doublets using (left) iterative LSI of scATAC-seq data, (middle) iterative LSI of scRNA-seq data, and (right) iterative LSI of combined scATAC-seq and scRNA-seq. Cells are colored by clusters identified from the combined analysis. Adjusted Rand Index (ARI) of clusters identified from (left) scATAC-seq and (middle) scRNA-seq compared to the combination are shown in the top right of each plot. **c-f**, Distribution of Pearson correlations of inferred gene score and aligned gene expression for (**c,e**) each gene or (**d,f**) each cell group across groups of 100 cells (N = 500 groups). Distributions are either presented for (**c,d**) the top 1,000 differentially expressed genes or (**e,f**) the top 2,000 most variable genes for each of the 57 gene score models tested. See Extended Data Fig. 6 for further details. **g**, Heatmaps summarizing the accuracy (measured by Pearson correlation) across all models for both the top 1,000 differentially expressed and top 2,000 variable genes for paired scATAC-seq and scRNA-seq data. Each entry is colored by the model rank in the given test as described below the heatmap. The model class is indicated to the left of each heatmap. **h**, UMAPs of scATAC-seq data from the Multiome PBMCs dataset (N = 9,702 cells) colored by (bottom) inferred gene scores or (top) gene expression for several marker genes. **i**, Heatmaps of (left) gene expression or (right) gene scores for the top 1,000 differentially expressed genes (selected from scRNA-seq) across all cell types from the paired scATAC-seq and scRNA-seq data.

After clustering, investigators aim to annotate the biological state related to each cluster. Methods for inferring gene expression from scATAC-seq data can generate ‘gene scores’ of key marker genes that can enable accurate cluster annotation^5–8,18^. However, the methods for converting chromatin accessibility signal to these gene score predictions were not extensively optimized. To this end, we used ArchR to benchmark 56 different models for inferring gene expression from scATAC-seq data using matched scATAC-seq and scRNA-seq data from PBMCs^12^ and bone marrow cells^7^ (Fig. 2a and Supplementary Table 3). To assess the performance of each model, we used canonical correlation analysis to integrate scATAC-seq and scRNA-seq data from the same sample types and then compared the linked gene expression from scRNA-seq to the inferred gene scores from scATAC-seq^7,12^. To establish this linkage, we used the canonical correlation analysis-based integration implemented in Seurat^12^ in both the PBMC and bone marrow datasets and labeled cells based on previously identified clusters^7,12^ (Fig. 2a). We then tested the 56 gene score models, which varied by the regions included, the sizes of those regions and the weights (based on genomic distance) applied to each region, using four different tests (Fig. 2b and Extended Data Fig. 6a–h). These tests assessed how the models performed in predicting differential gene expression across sets of genes or groups of cells (Fig. 2b). Although unweighted in our comparisons, the most informative of these tests assesses model performance in predicting gene expression changes among differentially expressed or highly variable genes, as these are likely to be cell-type-specific marker genes used in cluster annotation. Models that incorporated ATAC-seq signal from the gene body were more accurate than models that incorporated signal only from the promoter, likely due to the moderate increase in accessibility that occurs during active transcription. Moreover, incorporation of distal regulatory elements, weighted by distance, while accounting for the presence of neighboring genes (‘Gene Score Matrix’ in the Supplementary Information) improved the gene score inference in all cases (Extended Data Fig. 6a–h). The most accurate model across both datasets was model 42, a model within the ‘Gene Body Extended + Exponential Decay + Gene Boundary’ class of models (Fig. 2b), which integrates signal from the entire gene body and scales signal with bi-directional exponential decays from the gene TSS (extended upstream by 5 kb) and the gene transcription termination site while accounting for neighboring gene boundaries (Fig. 2c). This model yielded more accurate genome-wide gene score predictions in both PBMC and bone marrow cell datasets than did other models (Fig. 2d–f and Extended Data Fig. 6i,j). We additionally confirmed the efficacy of this class of gene score models using previously published matched bulk ATAC-seq and RNA-seq data from hematopoietic cells (Extended Data Fig. 6k–m)^32^, as well as paired single-cell data from PBMCs acquired with the 10x Genomics Multiome platform (Extended Data Fig. 7). Given this analysis, we implemented this class of gene score models (via model 42) for all downstream analyses involving inferred gene expression in ArchR.

### ArchR enables comprehensive analysis of massive-scale scATAC-seq data

**Fig. 3:**
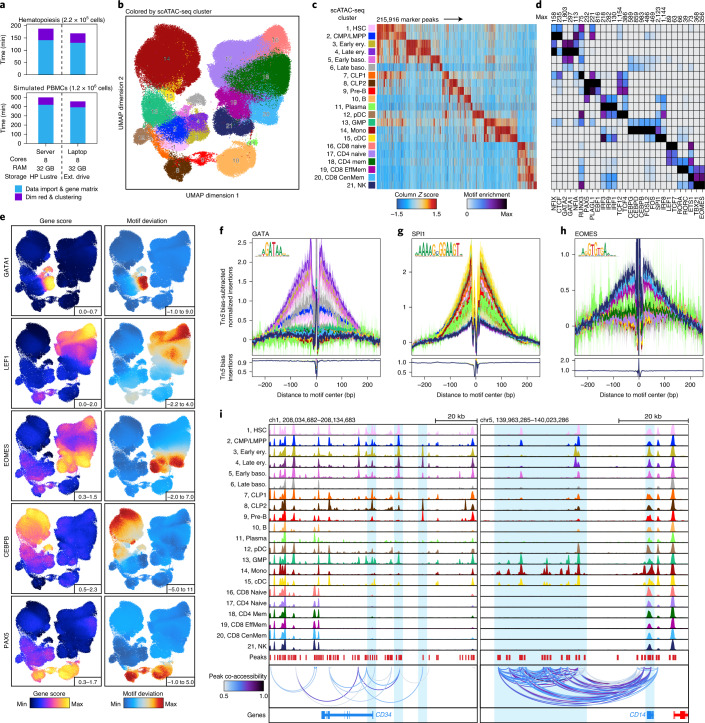
ArchR enables comprehensive analysis of massive-scale scATAC-seq data. **a**, Runtimes for ArchR-based analysis of over 220,000 and 1,200,000 single cells, respectively, using a small-cluster-based computational environment (32 GB of RAM and eight cores with HP Lustre storage) and a personal MacBook Pro laptop (32 GB of RAM and eight cores with an external (ext.) USB hard drive). Color indicates the relevant analytical step. **b**, UMAP of the hematopoiesis dataset colored by the 21 hematopoietic clusters. UMAP was constructed using LSI estimation with 25,000 landmark cells. **c**, Heatmap of 215,916 ATAC-seq marker peaks across all hematopoietic clusters identified with bias-matched differential testing. Color indicates the column *Z* score of normalized accessibility. **d**, Heatmap of motif hypergeometric enrichment-adjusted *P* values within the marker peaks of each hematopoietic cluster. Color indicates the motif enrichment (−log_10_ (*P* value)) based on the hypergeometric test. **e**, Side-by-side UMAPs of gene scores (left) and motif deviation scores for ArchR-identified TFs (right), for which the inferred gene expression is positively correlated with the chromVAR TF deviation across hematopoiesis. **f**–**h**, Tn*5* bias-adjusted TF footprints for GATA, proto-oncogene SPI1 and EOMES motifs, representing positive TF regulators of hematopoiesis. Lines are colored by the 21 clusters shown in **c**. **i**, Genome accessibility track visualization of marker genes with peak co-accessibility. Left, *CD34* genome track (chromosome (chr)1, 208,034,682–208,134,683) showing greater accessibility in earlier hematopoietic clusters (1–5, 7–8 and 12–13). Right, *CD14* genome track (chr5, 139,963,285–140,023,286) showing greater accessibility in earlier monocytic clusters (13–15).

ArchR is designed to handle datasets that are substantially larger (>1,000,000 cells) than those generated to date with modest computational resources. To illustrate this, we collected a compendium of published scATAC-seq data from hematopoietic cells generated with the 10x Chromium system and the Fluidigm C1 system (49 samples, ~220,000 cells; Supplementary Fig. 8a–d). Using both a small-scale server infrastructure (eight cores, 32 GB RAM, with a Hewlett-Packard (HP) Lustre file system) and a personal laptop (MacBook Pro laptop; eight cores, 32 GB RAM, with an external universal serial bus (USB) hard drive), ArchR performed data import, dimensionality reduction and clustering on ~220,000 cells in less than 3 h (Fig. 3a and Supplementary Fig. 8e). We next used ArchR to analyze a simulated set of over 1.2 million PBMCs, split into 200 individual samples. Under the same computational constraints, ArchR performed data import, dimensionality reduction and clustering of more than 1.2 million cells in under 8 h (Fig. 3a and Supplementary Fig. 8e). Using this dataset, we benchmarked the runtime and memory usage performance of ArchR across various cell numbers and total fragments to facilitate interpretation of end-user system requirements for datasets of different sizes (Supplementary Fig. 8f).

**Extended Data Fig. 8:**
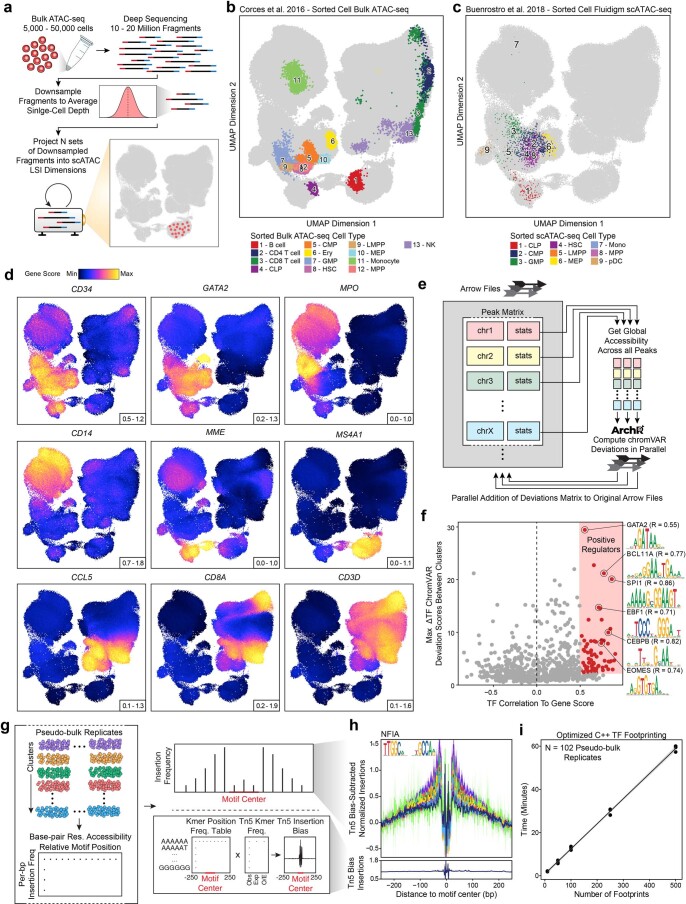
Analysis of the large hematopoiesis scATAC-seq dataset. **a**, Schematic for the projection of bulk ATAC-seq data into an existing single-cell embedding using LSI projection. Bulk ATAC-seq data (10–20 million fragments) is down sampled to a fragment number corresponding to the average single-cell experiment, and LSI-projected into the single-cell subspace. **b**, LSI projection of bulk ATAC-seq data from diverse hematopoietic cell types into the scATAC-seq embedding of the hematopoiesis dataset. **c-d**, UMAP of scATAC-seq data from the hematopoiesis dataset (N = 215,031 cells) colored by (**c**) sorted cells processed with the Fluidigm C1 system or (**d**) inferred gene scores for marker genes of hematopoietic cells. **e**, Schematic of the scalable chromVAR method implemented in ArchR. ArchR computes global accessibility within each peak and then computes chromVAR deviations for each sample independently. **f**, Dot plot showing the identification of positive TF regulators through correlation of chromVAR TF deviation scores and inferred gene scores in cell groups (Correlation > 0.5 and Deviation Difference in the top 50^th^ percentile). These TFs were additionally filtered by the maximum observed deviation score difference observed across each cluster average to remove TFs that are correlated but do not have large accessibility changes in hematopoiesis. **g**, Schematic of TF footprinting with Tn5 bias correction in ArchR. Base-pair resolution insertion coverage files from sample-aware pseudo-bulk replicates are used to compute the insertion frequency around each motif for each replicate. For each motif, the total observed k-mers relative to the motif center per bp are identified. This k-mer position frequency table can then be multiplied by the individual sample Tn5 k-mer frequencies to compute the Tn5 insertion bias per replicate. **h**, TF footprint for the NFIA motif. Lines are colored by cluster identity from the hematopoiesis dataset shown in Fig. 3b. **i**, Benchmarking of run time for TF footprinting with ArchR for the 102 sample-aware pseudo-bulk replicates from the hematopoiesis dataset.

Beyond these straightforward analyses, ArchR also provides an extensive suite of tools for more comprehensive analysis of scATAC-seq data. Estimated LSI of this ~220,000-cell hematopoiesis dataset recapitulated the overall structure of the data with as few as 500 landmark cells (Supplementary Fig. 8g). Inspection of the resultant clusters using uniform manifold approximation and projection (UMAP)^33^ led us to use the 25,000-cell landmark set (~10% of total cells). This dimensionality reduction illustrated the utility of estimated LSI in minimizing some batch effects, with minimal bias observed, considering that the ~220,000-cell dataset was collected from multiple laboratories and technological platforms (Fig. 3b and Supplementary Fig. 8h–j). We identified 21 clusters spanning the hematopoietic hierarchy, calling clusters for even rare cell types, such as plasma cells, which comprise ~0.1% (265 cells) of the total population. To generate a universal peak set from cluster-specific peaks, ArchR creates sample-aware pseudo-bulk replicates that recapitulate the biological variability within each cluster (Supplementary Fig. 9a). A non-overlapping peak set was then identified from these pseudo-bulk replicates using an iterative overlap-merging procedure^34^ (Supplementary Fig. 9b). We identified 396,642 total reproducible peaks (Supplementary Fig. 9c), of which 215,916 were classified as differentially accessible peaks across the 21 clusters after differential testing (Fig. 3c and ‘Marker Peak Identification’ in the Supplementary Information). Motif enrichment within these peaks revealed known TF regulators of hematopoiesis, such as the transcription factor GATA1 in erythroid populations, CCAAT enhancer-binding protein β (CEBPB) in monocytes and paired box (PAX)5 in B cell differentiation (Fig. 3d). ArchR can additionally calculate peak overlap enrichment with a compendium of previously published ATAC-seq datasets^32,34–39^, identifying enrichment of peaks consistent with the cell type of each cluster (Supplementary Fig. 9d). To further characterize clusters, ArchR enables the projection of bulk ATAC-seq data into the single-cell-derived UMAP embedding^7^ (Extended Data Fig. 8a). This allows for the identification of the hematopoietic clusters based on well-validated bulk ATAC-seq profiles^4,32^ and aligns with inferred gene scores for canonical hematopoietic marker genes (Extended Data Fig. 8b–d).

ArchR also implements a scalable improvement of the chromVAR^16^ method for determining TF deviations (Extended Data Fig. 8e). TFs for which the expression is highly correlated with motif accessibility can therefore be identified based on the correlation of the inferred gene score to the chromVAR motif deviation. This analysis identifies known drivers of hematopoietic differentiation, such as GATA1 in erythroid populations, lymphoid enhancer-binding factor (LEF)1 in naive T cell populations and eomesodermin (EOMES) in natural killer and/or memory T cell populations. (Fig. 3e, Extended Data Fig. 8f and Supplementary Table 4). ArchR also enables rapid footprinting of TF regulators within clustered subsets while accounting for Tn*5* biases^34^ using an improved C++ implementation (Fig. 3f–h and Extended Data Fig. 8g–i). Finally, ArchR identifies links between regulatory elements and target genes based on the co-accessibility of pairs of loci across single cells^1,18^ (Fig. 3i).

### The interactive ArchR genome browser

In addition to these ATAC-seq analysis paradigms, ArchR provides a fully integrated and interactive genome browser (Supplementary Fig. 10a). The interactive nature of the browser is enabled by the optimized storage format within each Arrow file, providing support for dynamic cell grouping, track resolution, coloration, layout and more. Launched by a single command, the ArchR browser enables cell cluster investigations of marker genes, such as *CD34* for early hematopoietic stem and progenitor cells and *CD14* for monocytic populations (Fig. 3i and Supplementary Fig. 10b–e), mitigating the need for external software.

### ArchR enables integration of matched scRNA-seq and scATAC-seq datasets

**Fig. 4:**
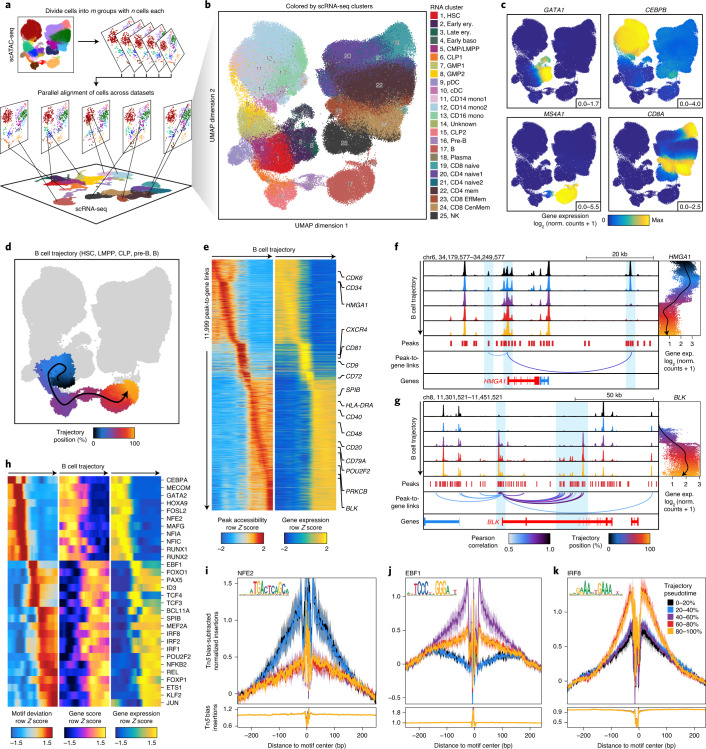
Integration of scATAC-seq and scRNA-seq data by ArchR identifies gene regulatory trajectories of hematopoietic differentiation. **a**, Schematic of scATAC-seq alignment with scRNA-seq data in *m* slices of *n* single cells. These slices are independently aligned to a reference scRNA-seq dataset and then the results are combined for downstream analysis. This integrative design facilitates rapid large-scale integration with low memory requirements. **b**–**d**, UMAPs of scATAC-seq data from the hematopoiesis dataset colored by alignment to previously published hematopoietic scRNA-seq-derived clusters (**b**), integrated scRNA-seq gene expression for key marker TFs and genes (**c**) or cell alignment to the ArchR-defined B cell trajectory (**d**). In **d**, the smoothed arrow represents a visualization of the interpreted trajectory (determined in the LSI subspace) in the UMAP embedding. **e**, Heatmap of 11,999 peak-to-gene links identified across the B cell trajectory with ArchR. **f**,**g**, Genome track visualization of the *HMGA1* locus (chr6, 34,179,577–34,249,577) (**f**) and the *BLK* locus (chr8, 11,301,521–11,451,521) (**g**). Single-cell gene expression (exp.) across pseudotime in the B cell trajectory is shown to the right. Inferred peak-to-gene links for distal regulatory elements across the hematopoiesis dataset are shown below. **h**, Heatmap of positive TF regulators for which gene expression is positively correlated with chromVAR TF deviation across the B cell trajectory. **i**–**k**, Tn*5* bias-adjusted TF footprints for nuclear factor, erythroid (NFE)2 (**i**), early B cell factor (EBF)1 (**j**) and interferon regulatory factor (IRF)8 (**k**) motifs, representing positive TF regulators across the B cell trajectory. Lines are colored by the position in pseudotime of B cell differentiation.

**Extended Data Fig. 9:**
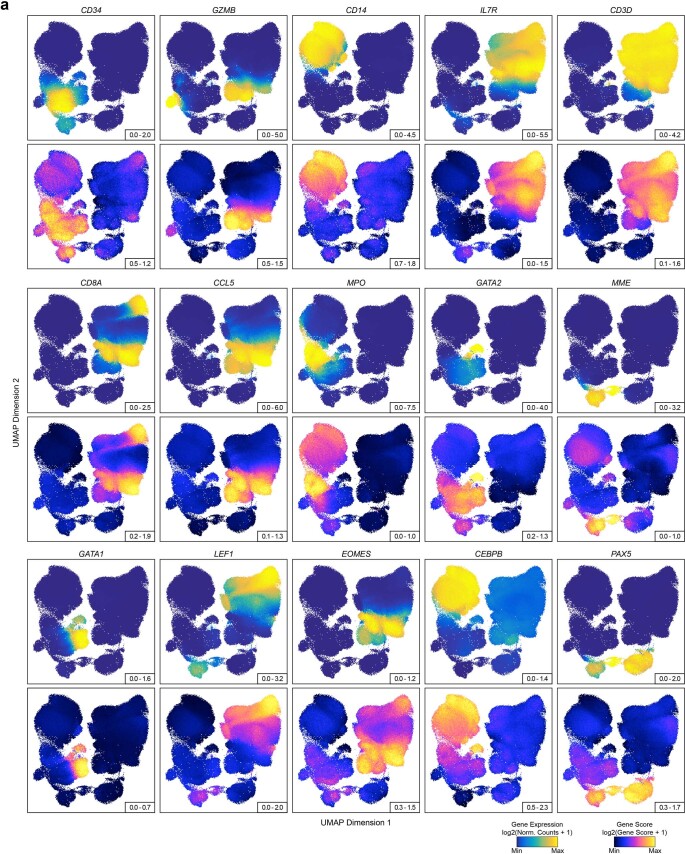
Cross-platform integration in ArchR enables linkage of gene expression and chromatin accessibility in cell type-specific marker genes. **a**, Side-by-side UMAPs for the hematopoiesis dataset cells colored by (top) gene expression (log2(Normalized Counts +1)) from scRNA-seq alignment or (bottom) inferred gene scores (log2(Gene Score +1)) from gene score Model 42 (see Fig. 2c) for key immune marker genes.

**Extended Data Fig. 10:**
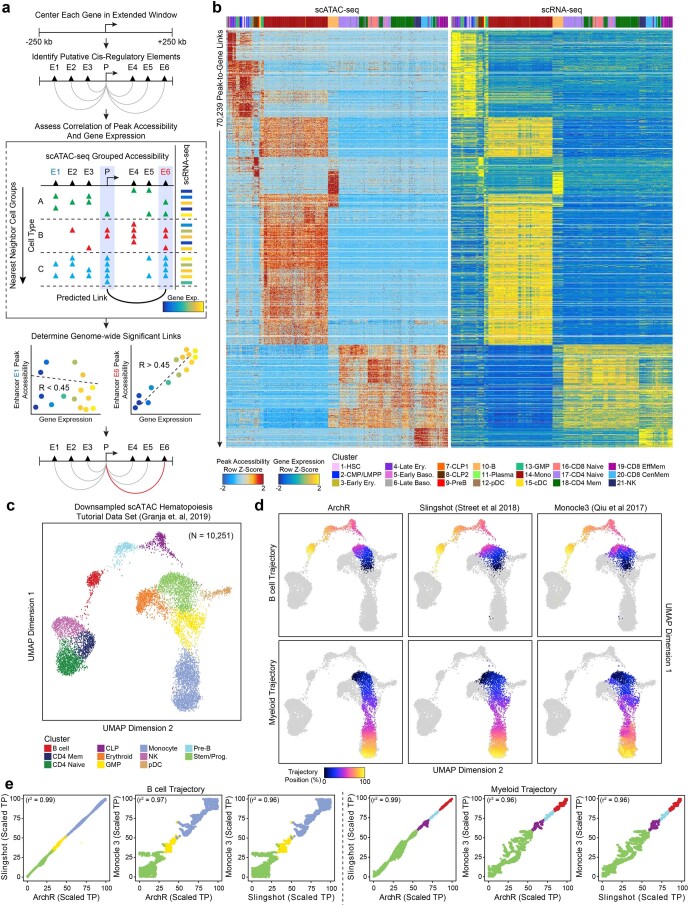
Peak-to-gene linkage and trajectory analysis in the large hematopoiesis dataset. **a**, Schematic of identification of peak-to-gene links with ArchR. First, all combinations of peak-to-gene linkages are identified. Second, the peak accessibility and gene expression for cell groups are calculated. Finally, all potential peak-to-gene linkages are tested and significant links (R > 0.45 and FDR < 0.1) are kept. **b**, Heatmap of 70,239 peak-to-gene links identified across the hematopoiesis dataset with ArchR. **c**, UMAP of scATAC-seq data from a subset of cells derived from Granja et al. 2019^7^. This data is the same as the hematopoietic tutorial data set (N = 10,251) used in the ArchR user manual. Cells are colored by ArchR identified clusters. **d**, UMAP as shown in Extended Data Fig. 10c but colored by trajectory position along the (top) B cell trajectory and (bottom) Myeloid trajectory for (left) ArchR, (middle) Slingshot, and (right) Monocle3. **e**, One-to-one comparisons of ArchR, Slingshot and Monocle3 scaled trajectory positions (Scaled TP) across the (left) B cell trajectory and (right) Myleoid trajectory.

ArchR also provides functionality to integrate scATAC-seq and scRNA-seq data using Seurat’s infrastructure, matching the heterogeneous chromatin accessibility profiles and RNA expression^12^. Single-cell epigenome-to-transcriptome integration is essential for understanding dynamic gene regulatory processes, and we anticipate this sort of analysis will become even more prevalent with the advent of platforms for simultaneous scATAC-seq and scRNA-seq. ArchR performs this cross-data alignment in parallel using slices of the scATAC-seq data (Fig. 4a). When performed on the hematopoiesis dataset, this integration enabled scRNA-seq alignment for >220,000 cells in less than 1 h (Fig. 4b). We note that this dataset represents a diverse collection of experiments from different laboratories and technological platforms that is not ideal for high-resolution integration because the large intersample heterogeneity obscures the accuracy of the cross-platform alignment. The alignment showed high concordance between linked gene expression and inferred gene scores for common hematopoietic marker genes (Fig. 4c and Extended Data Fig. 9a). Using this cross-platform alignment, ArchR also provides methods to identify putative *cis*-regulatory elements based on correlated peak accessibility and gene expression, identifying 70,239 significant peak-to-gene linkages across the hematopoietic hierarchy^7,34^ (Extended Data Fig. 10a,b and Supplementary Table 5).

Finally, ArchR facilitates cellular trajectory analysis to identify the predicted path of gene regulatory changes from one set of cells to another, a unique type of insight enabled by single-cell data. In addition to implementing both Slingshot^40^ and Monocle 3 (refs. ^41–43^), two scRNA-seq trajectory algorithms, ArchR also provides its own supervised trajectory analysis. To do this analysis, ArchR initially creates a cellular trajectory based on the average positions (within a lower *n*-dimensional subspace) of a sequence of user-supplied clusters or groups. ArchR then aligns individual cells to this trajectory by computing the nearest cell-to-trajectory distance^6^. We benchmarked the performance of trajectory analysis in ArchR compared to those in Slingshot and Monocle 3 using a miniaturized version of the hematopoiesis dataset (*n* = 10,251) (Extended Data Fig. 10c). We compared the learned trajectories from stem and progenitor cells through differentiated B cells or monocytes and found that the inferred trajectories were highly similar (*r*^2^ > 0.96) (Extended Data Fig. 10d,e). The implementation of all three trajectory algorithms allows end users to select the implementation that best suits their analysis, as each trajectory method has distinct advantages. To demonstrate trajectory analysis in ArchR on the full hematopoiesis dataset, we again focused on the B cell lineage as an example (Fig. 4d). ArchR traces cells along the B cell differentiation trajectory and identifies 11,999 peak-to-gene links that have correlated regulatory dynamics (*r* > 0.5) across the B cell differentiation trajectory (Fig. 4e). Sequencing tracks of the *HMGA1* gene locus, active in stem and progenitor cells, and the *BLK* locus, active in differentiated B cells, demonstrate how accessibility at linked peaks correlates with longitudinal changes in gene expression across pseudotime (Fig. 4f,g). ArchR can then identify TF motifs for which accessibility is positively correlated with the gene expression of the corresponding TF gene (*r* > 0.5) across the same B cell trajectory (‘Large Hematopoiesis 220K Cells’ in the Supplementary Information) (Fig. 4h). TF footprinting of a subset of these TFs further illustrates the dynamics in local accessibility at the binding sites of these lineage-defining TFs across B cell differentiation pseudotime (Fig. 4i–k).

## Discussion

Chromatin accessibility data provides a lens through which we can observe the gene regulatory programs that underlie cellular state and identity. The highly cell-type-specific nature of *cis*-regulatory elements makes profiling of single-cell chromatin accessibility an attractive method to understand cellular heterogeneity and the molecular processes underlying complex control of gene expression. With the advent of methods to profile chromatin accessibility across thousands of single cells, scATAC-seq quickly became a method of choice for many single-cell applications. However, compared to scRNA-seq, for which tools such as Seurat^12^, Monocle^41^ and Scanpy^44^ have gained widespread use, the analysis of scATAC-seq data is a comparatively newer field. Many existing tools facilitate certain aspects of the scATAC-seq workflow^11–19^ but are not suited for the scale of current data generation efforts (>80,000 cells) or do not support the breadth of analytical functionalities that would facilitate wider adoption of this technique.

To address this need, we developed ArchR, an end-to-end software solution that will expedite single-cell chromatin analysis for any biologist. Low memory usage, parallelized operations and an extensive tool suite make ArchR an ideal platform for scATAC-seq data analysis. In contrast to currently available software packages, ArchR is designed to handle millions of cells using commonly available computational resources, such as a laptop running a Unix-based operating system. As such, ArchR provides the analytical support necessary for the massive scale of ongoing efforts to catalog the compendium of diverse cell types at single-cell resolution^45^. In addition to the dramatic improvements in runtime, memory efficiency and scale, ArchR supports state-of-the-art chromatin-based analyses, including genome-wide inference of gene activity, TF footprinting and data integration with matched scRNA-seq, enabling statistical linkage of *cis*- and *trans*-acting regulatory factors to gene expression profiles. Moreover, the improvements from ArchR enable interactive data analysis by which end users can iteratively adjust analytical parameters and thus optimize identification of biologically meaningful results. This is especially important in the context of single-cell data for which a one-size-fits-all analytical pipeline is not relevant or desirable. Supervised identification of clusters, resolution of subtle batch effects and biology-driven data exploration are intrinsically necessary for a successful scATAC-seq analysis, and ArchR supports these efforts by enabling rapid analytical processes. ArchR provides an open-source analysis platform with the flexibility, speed and power to support the rapidly increasing efforts to understand complex tissues, organisms and ecosystems at the resolution of individual cells.

## Methods

### Genome and transcriptome annotations

All analyses were performed with the hg19 genome (except for the Mouse Atlas, with mm9). R-based analysis used the BSgenome package with ‘BSgenome.Hsapiens.UCSC.hg19’ (‘BSgenome.Mmusculus.UCSC.mm9’ for Mouse Atlas) for genomic coordinates and the TxDb package with ‘TxDb.Hsapiens.UCSC.hg19.knownGene’ (‘TxDb.Mmusculus.UCSC.mm9.knownGene’ for Mouse Atlas) gene annotations unless otherwise stated.

### Cell type abbreviations

In many of the figure legends, abbreviations are used for cell types of the hematopoietic system. HSC, hematopoietic stem cell; LMPP, lymphoid-primed multipotent progenitor cell; B, B cell; B cell prog., B cell progenitor; CMP, common myeloid progenitor; CLP, common lymphoid progenitor; GMP, granulocyte macrophage progenitor; CD4 mem, CD4 memory T cell; CD4 naive, CD4 naive T cell; CD8 naive, CD8 naive T cell; CD8 eff, CD8 effector T cell; CD8 EffMem, CD8 effector memory T cell; CD8 CenMem, CD8 central memory T cell; DN T cell, double-negative T cell; mono, monocyte; plasma, plasma cell; pDC, plasmacytoid dendritic cell; pre-B; pre-B cell; NK, natural killer cell; ery, erythroid; baso, basophil.

### scATAC-seq data generation: cell lines

With the exception of MCF10A, all cell lines were cultured in the designated medium containing 10% FBS and penicillin–streptomycin. Jurkat, THP-1 and K562 cell lines were ordered from ATCC and cultured in RPMI 1640. GM12878 cells were ordered from Coriell and cultured in RPMI 1640. HeLa, HEK293T and HT1080 cell lines were ordered from ATCC and cultured in DMEM. T24 cells were ordered from ATCC and cultured in McCoy’s 5A medium. MCF7 cells were ordered from ATCC and cultured in EMEM containing 0.01 mg ml^−1^ human insulin (MilliporeSigma, 91077C). MCF10A cells were ordered from ATCC and cultured in DMEM/F12 medium containing 5% horse serum (Thermo Fisher, 16050130), 0.02 µg ml^−1^ human EGF (PeproTech, AF-100-15), 0.5 µg ml^−1^ hydrocortisone (MilliporeSigma, H0888), 0.1 µg ml^−1^ cholera toxin (MilliporeSigma, C8052), 10 µg ml^−1^ insulin from bovine pancreas (MilliporeSigma, I6634) and penicillin–streptomycin. Cultured cells were viably cryopreserved in aliquots of 100,000 cells using 100 µl BAMBANKER freezing medium (Wako Chemicals, 302-14681) so that scATAC-seq could be performed on all cells at the same time. For each cell line, cells were thawed with the addition of 1 ml ice-cold resuspension buffer (RSB) (10 mM Tris-HCl, pH 7.4, 10 mM NaCl, 3 mM MgCl_2_) containing 0.1% Tween-20 (RSB-T). Cells were pelleted in a fixed-angle rotor at 300 r.c.f. for 5 min at 4 °C. The supernatant was removed, and the pellet was resuspended in 100 µl ice-cold lysis buffer (RSB-T containing 0.1% NP-40 and 0.01% digitonin) and incubated on ice for 3 min. To dilute the lysis reaction, 1 ml chilled RSB-T was added to each tube, and the cells were pelleted as before. The supernatant was removed, and the pelleted nuclei were resuspended in Diluted Nuclei Buffer (10x Genomics). The nuclei stock concentration was determined for each cell line using trypan blue, and a total of 5,000 nuclei from each cell line were pooled together and loaded into the 10x Genomics scATAC-seq (version 1) transposition reaction. The remainder of the scATAC-seq library preparation was performed as recommended by the manufacturer. Resultant libraries were sequenced on an Illumina NovaSeq 6000 using an S4 flow cell and paired-end 99-bp reads. In addition to this pooled scATAC-seq library, each cell line was used to generate bulk ATAC-seq libraries as described previously^39^. Bulk ATAC-seq libraries were pooled and purified by polyacrylamide gel electrophoresis before sequencing on an Illumina HiSeq 4000 using paired-end 75-bp reads.

### scATAC-seq processing: cell line mixing

Raw sequencing data was converted to FastQ format using the ‘cellranger-atac mkfastq’ pipeline (10x Genomics, version 1.0.0). scATAC-seq reads were aligned to the hg19 reference genome (https://support.10xgenomics.com/single-cell-atac/software/downloads/latest) and quantified using the ‘cellranger-count’ pipeline (10x Genomics, version 1.0.0). Genotypes used to perform demuxlet were determined as follows for each cell line: bulk ATAC-seq FastQ files were processed and aligned using PEPATAC (http://code.databio.org/PEPATAC/) as described previously^34^. Peaks were identified using MACS2, and a union set of variable-width accessible regions was identified using bedtools merge (version 2.26.0). These accessible regions were genotyped across all samples using SAMtools mpileup (version 1.5) and VarScan mpileup2snp (version 2.4.3) with the following parameters: ‘--min-coverage 5 --min-reads2 2 --min-var-freq 0.1 --strand-filter 1 --output-vcf 1’. All positions containing a single-nucleotide variant were compiled into a master set, and then each cell line was genotyped at those specific single-base locations using SAMtools mpileup. The allelic depth at each position was converted into a quaternary genotype (homozygous A, heterozygous AB, homozygous B or insufficient data to generate a confident call). Next, for each cell line, inferred genotype probabilities were created based on those quaternary genotypes, and a VCF file was created for input to demuxlet using recommended parameters. Demuxlet was used to identify the cell line of origin for individual cells and to identify doublets based on mixed genotypes.

### Reporting Summary

Further information on research design is available in the Nature Research Reporting Summary linked to this article.

## Online content

Any methods, additional references, Nature Research reporting summaries, source data, extended data, supplementary information, acknowledgements, peer review information; details of author contributions and competing interests; and statements of data and code availability are available at 10.1038/s41588-021-00790-6.

## Supplementary information

Supplementary Information Supplementary Figs. 1–11 and Methods

Reporting Summary

Supplementary Table 1 Supplementary Tables 1–5

## Data Availability

Bulk and scATAC-seq data from the cell line-mixing experiment are available under GEO accession number GSE162690. All other scATAC-seq data used were from publicly available sources as outlined in Supplementary Table 1. We additionally made other analysis files available on our publication page at https://github.com/GreenleafLab/ArchR_2020.

## Code availability

Extensive documentation and a full user manual are available at https://www.archrproject.com/. The software is open source, and all code can be found on GitHub at https://github.com/GreenleafLab/ArchR. Additionally, code for reproducing the majority of analyses from this paper is available at the publication page https://github.com/GreenleafLab/ArchR_2020.
