# Author Correction: ArchR is a scalable software package for integrative single-cell chromatin accessibility analysis

**DOI:** 10.1038/s41588-021-00850-x

**Published:** 2021-03-31

**Authors:** Jeffrey M. Granja, M. Ryan Corces, Sarah E. Pierce, S. Tansu Bagdatli, Hani Choudhry, Howard Y. Chang, William J. Greenleaf

**Affiliations:** 1 Department of Genetics, Stanford University School of Medicine, Stanford, CA USA; 2 Program in Biophysics, Stanford University, Stanford, CA USA; 3 Center for Personal Dynamic Regulomes, Stanford University, Stanford, CA USA; 4 Department of Pathology, Stanford University School of Medicine, Stanford, CA USA; 5 Gladstone Institute of Neurological Disease, Gladstone Institute of Data Science and Biotechnology, San Francisco, CA USA; 6 Department of Neurology, University of California San Francisco, San Francisco, CA USA; 7 Program in Cancer Biology, Stanford University School of Medicine, Stanford, CA USA; 8 Department of Biochemistry, Faculty of Science, Cancer and Mutagenesis Unit, King Fahd Center for Medical Research, King Abdulaziz University, Jeddah, Saudi Arabia; 9 Howard Hughes Medical Institute, Stanford University, Stanford, CA USA; 10 Department of Applied Physics, Stanford University, Stanford, CA USA; 11 Chan Zuckerberg Biohub, San Francisco, CA USA

**Keywords:** Epigenetics, Gene regulation, Epigenomics

Correction to: *Nature Genetics* 10.1038/s41588-021-00790-6, published nline 25 February 2021.

This paper was originally published without open access. As of the date of this correction, the paper is available online as an open-access paper under a Creative Commons Attribution 4.0 International License. The Acknowledgements have additionally been updated to specify the funding agency for NCI Cancer Moonshot grant U2CCA233311.

