# Supplementary material for: ArchR is a scalable software package for integrative single-cell chromatin accessibility analysis
Source: Nat Genet. 2021 Feb 25;53(3):403–11. doi: 10.1038/s41588-021-00790-6 (PMC8012210; doi:10.1038/s41588-021-00790-6)
Supplement: Supplementary file 1 — Supplementary Figs. 1–11 and Methods [file 41588_2021_790_MOESM1_ESM.pdf]

nature portfolio

https://doi.org/10.1038/s41588-021-00790-6

Supplementary information

ArchR is a scalable software package for integrative single-cell chromatin accessibility analysis

In the format provided by the authors and unedited

SUPPLEMENTARY INFORMATION FOR:

ArchR is a scalable software package for integrative single-cell chromatin accessibility analysis

Jeffrey M. Granja*,✉, M. Ryan Corces*, Sarah E. Pierce, S. Tansu Bagdatli, Hani Choudhry, Howard Y. Chang✉, William J. Greenleaf✉

✉Correspondence should be addressed to J.M.G (jgranja.stanford@gmail.com), H.Y.C. (howchang@stanford.edu), or W.J.G (wjg@stanford.edu).

This document contains the following:

Page Numbers	Content
2-21	Supplementary Figures and Legends 1-11
22-43	Supplementary Methods
44	Supplementary References Cited

SUPPLEMENTARY FIGURES AND LEGENDS

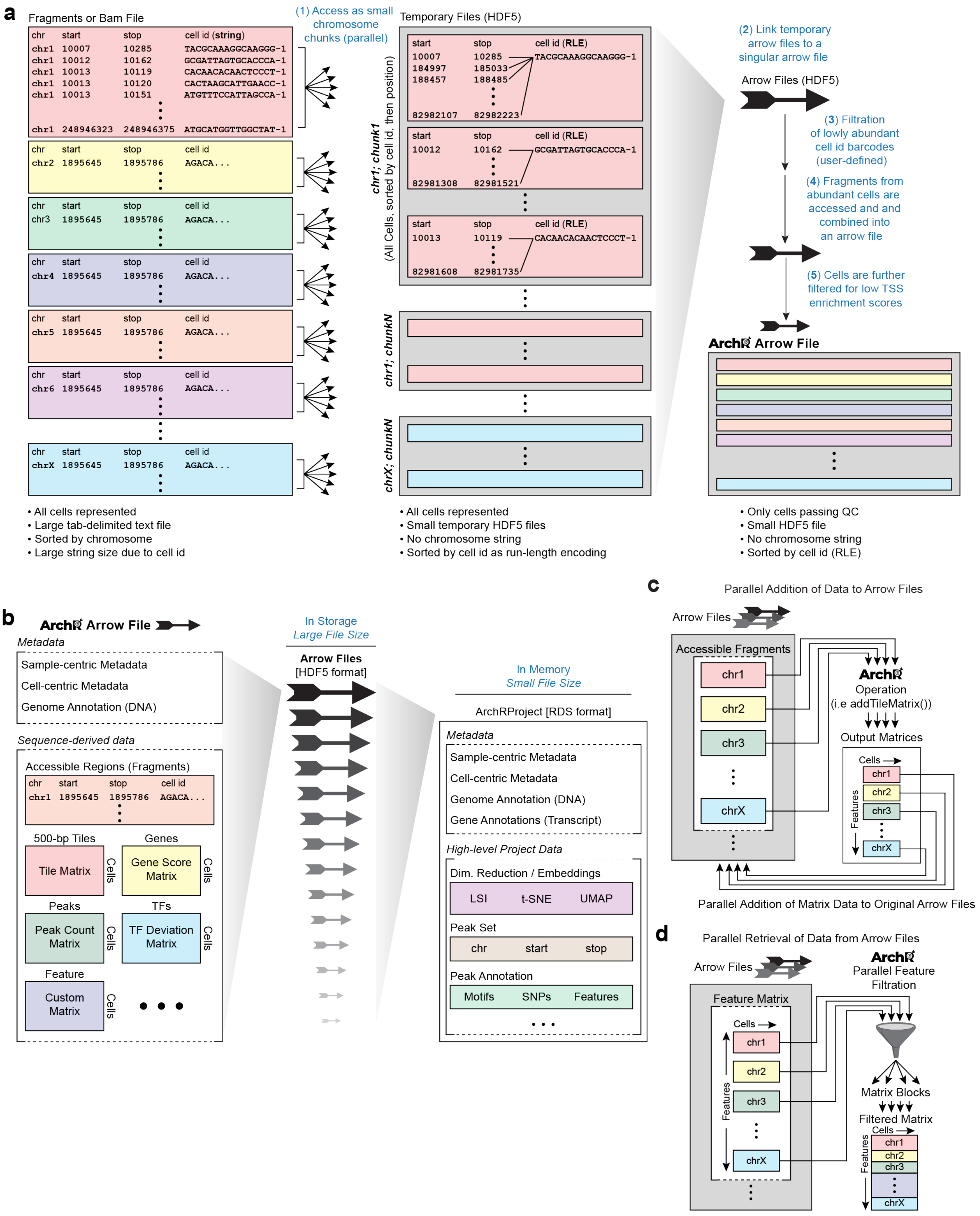

Supplementary Fig. 1 – Schematics of file infrastructure and information access in ArchR
a. Schematic of ArchR Arrow file creation from fragments or BAM file. First, the input file is accessed in parallel chromosome chunks and stored as temporary HDF5 files. These temporary Arrow files are then linked to a singular Arrow file. This linkage does not require reading and writing. Next, lowly-abundant barcodes are identified and fragments corresponding to the cells with more fragments than a user-defined minimum are read and written to a new Arrow file. Lastly, cells that have TSS enrichment scores below a user-defined threshold are discarded, resulting in a final efficiently organized Arrow file. RLE = run length encoding.
b. (Left) Schematic of the ArchR Arrow file format where accessible reads and arrays are organized within. Arrow files can then be used as input for an ArchRProject (Right). The ArchRProject stores the locations of these Arrow files and extracts their cell-centric metadata. All analysis with ArchR operates through this ArchRProject which can readily access data from Arrow files stored on disk.
c. Schematic demonstrating how ArchR operations that involve using Arrow fragments (i.e. addTileMatrix) operate on each chromosome independently in parallel for many Arrow files and then add the resulting matrix back to the corresponding Arrow files again in parallel.
d. Schematic demonstrating how ArchR operations that use Arrow matrices (i.e. addIterativeLSI) access a subset of each chromosome's matrix from each Arrow file in parallel that are then merged to create a filtered matrix for subsequent analysis.

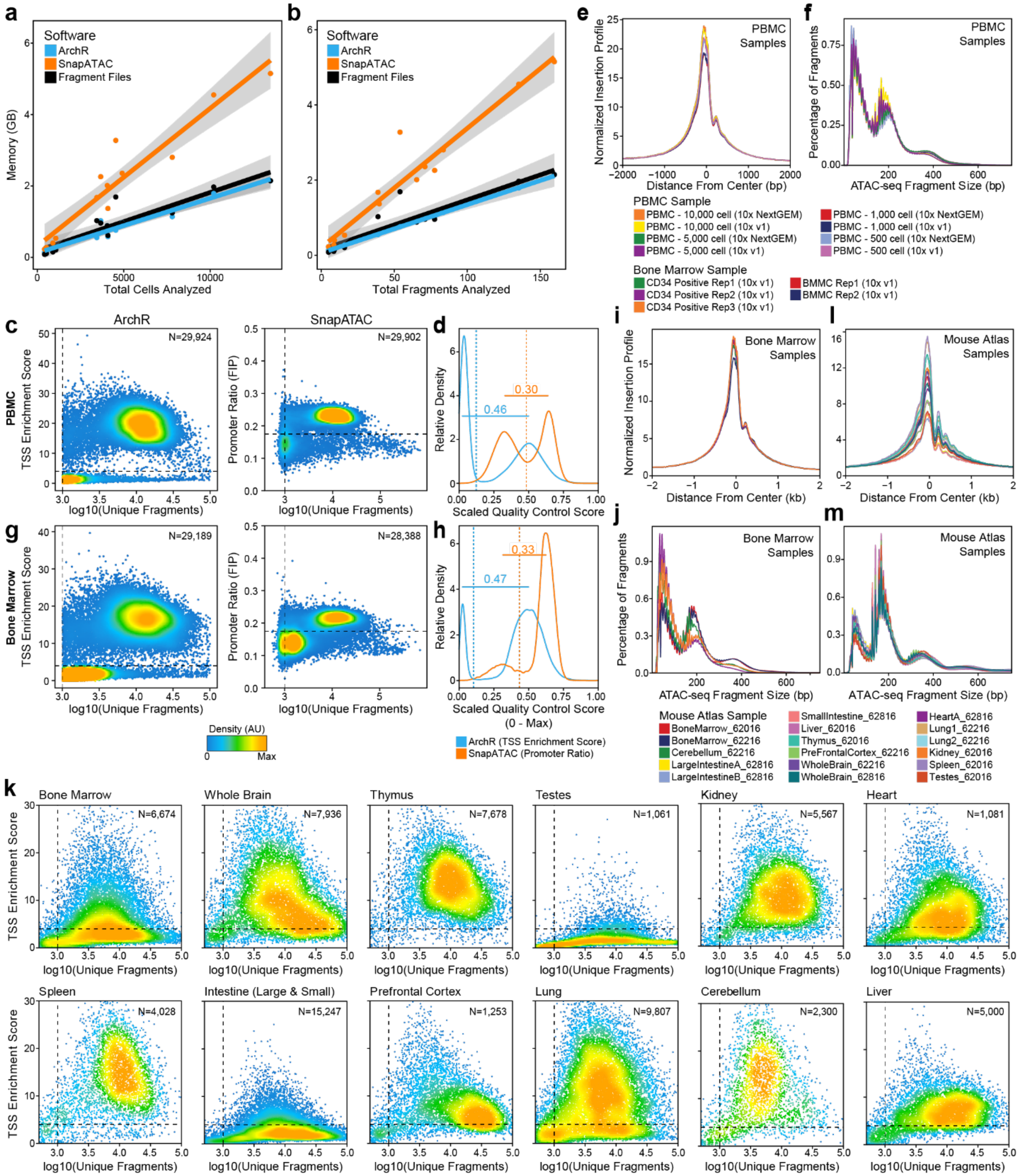

Supplementary Fig. 2 – Quality control metrics for PBMC, bone marrow, and mouse atlas datasets.

a-b. File sizes of storage formats (for both accessible fragments and counts matrix) for ArchR and SnapATAC compared to (**a**) the total number of cells they represent or (**b**) the total number of fragments corresponding to the cells represented in each file. Line colors represent the different software used or the original fragment files (shading represents 95% confidence interval).

c,g. QC filtering plots for the (**c**) PBMCs dataset or (**g**) bone marrow dataset from (left) ArchR, showing the TSS enrichment score vs unique nuclear fragments per cell, or (right) SnapATAC, showing the promoter ratio / fraction of reads in promoters (FIP) vs unique nuclear fragments per cell. Dot color represents the density in arbitrary units of points in the plot.
d,h. Scaled QC scores for ArchR (TSS enrichment score) and SnapATAC (promoter ratio) from the (**d**) PBMC dataset and (**h**) bone marrow dataset. The dotted line represents the cutoff for pass-filter cells. Horizontal labeled lines indicate the scaled difference between the center points of the pass-filter and failed cells, illustrating the differences in separation between these two QC metrics.
e-f, i-j, l-m. Aggregate (**e,i,l**) TSS insertion profiles centered at all TSS regions or (**f,j,m**) fragment size distributions for the cells passing ArchR QC thresholds for each sample in the (**e-f**) PBMCs dataset, (**i-j**) the bone marrow dataset, or (**l-m**) the mouse atlas dataset. Line color represents the sample from the dataset as indicated below or above the plot.
k. QC filtering plots from ArchR for each individual organ type from the mouse atlas dataset showing the TSS enrichment score vs unique nuclear fragments per cell. Dot color represents the density in arbitrary units of points in the plot.

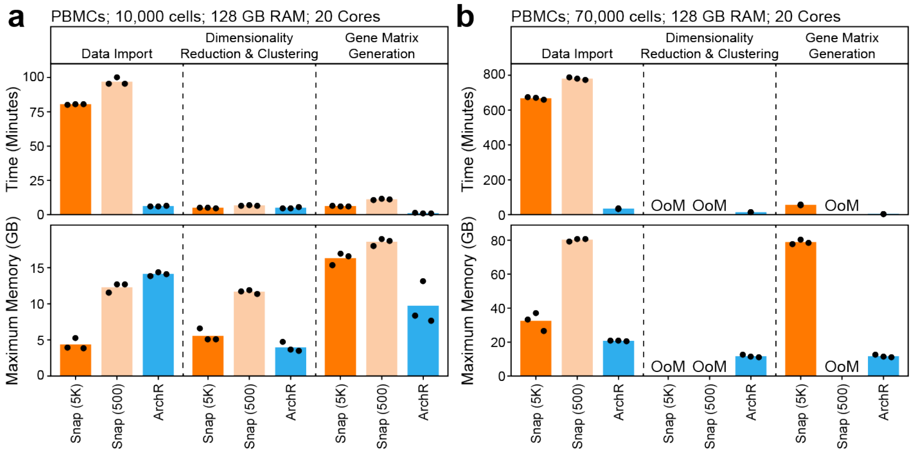

Supplementary Fig. 3 – Benchmarking of SnapATAC performance using higher-resolution genome-wide tile matrices.

a-b. Comparison of ArchR, SnapATAC with a 5-Kbp tile matrix, and SnapATAC with a 500-bp tile matrix for run time and peak memory usage for the analysis of (**a**) ~10,000 cells from the PBMCs dataset using 128 GB of RAM and 20 cores and (**b**) ~70,000 cells from the PBMCs dataset using 128 GB of RAM and 20 cores. Dots represent individual replicates of benchmarking analysis (N = 3). OoM = out of memory.

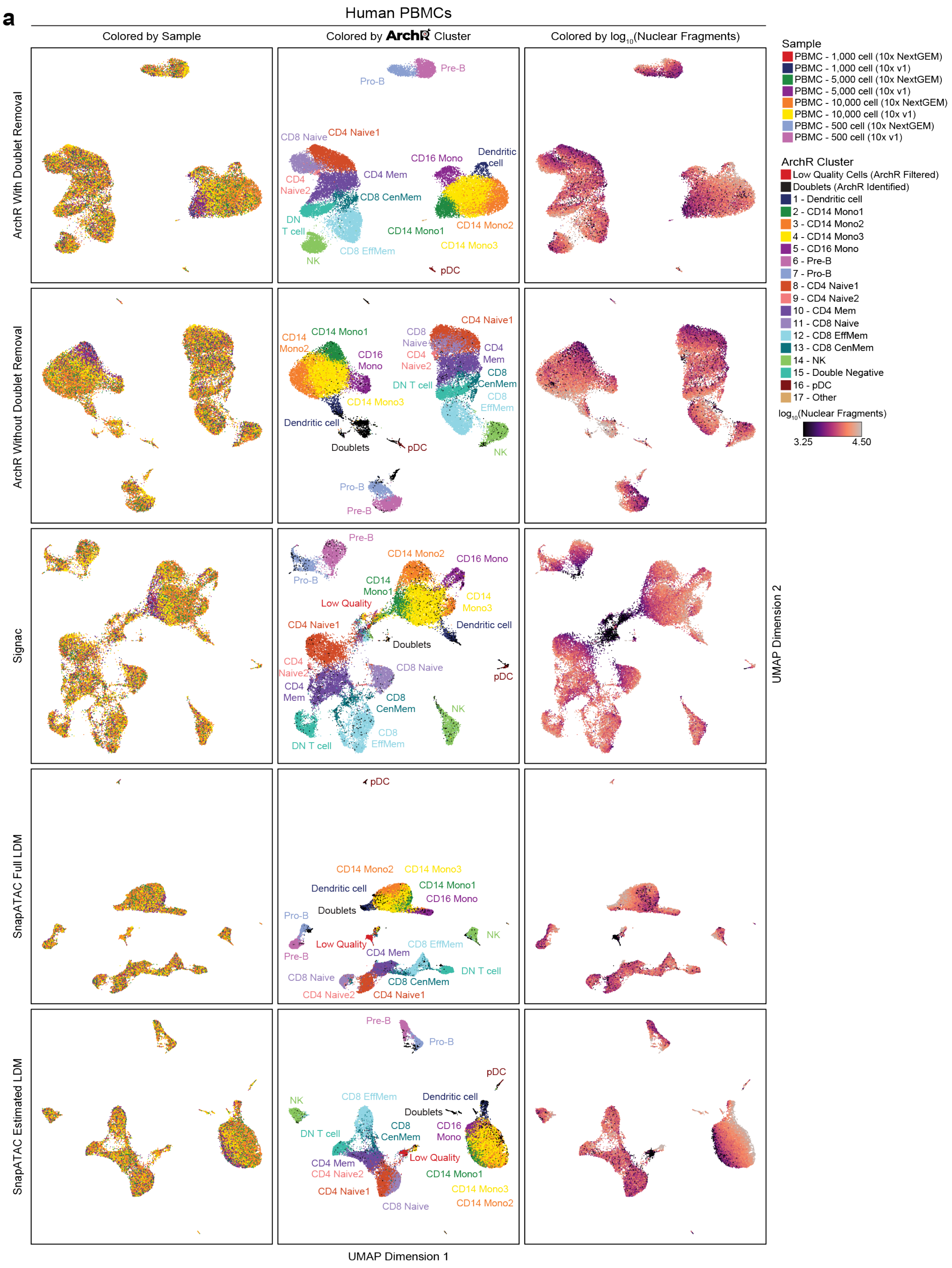
a
Human PBMCs
Colored by Sample
Colored by ArchR Cluster
Colored by log10(Nuclear Fragments)
ArchR With Doublet Removal
ArchR Without Doublet Removal
Signac
SnapATAC Full LDM
SnapATAC Estimated LDM
UMAP Dimension 1
UMAP Dimension 2
Pre-B
Pro-B
CD8 Naive
CD4 Naive1
CD4 Naive2
CD4 Mem
CD8 CenMem
CD8 EffMem
DN T cell
NK
CD16 Mono
Dendritic cell
CD14 Mono1
CD14 Mono2
CD14 Mono3
pDC
Doublets
Low Quality
Sample
PBMC - 1,000 cell (10x NextGEM)
PBMC - 1,000 cell (10x v1)
PBMC - 5,000 cell (10x NextGEM)
PBMC - 5,000 cell (10x v1)
PBMC - 10,000 cell (10x NextGEM)
PBMC - 10,000 cell (10x v1)
PBMC - 500 cell (10x NextGEM)
PBMC - 500 cell (10x v1)
ArchR Cluster
Low Quality Cells (ArchR Filtered)
Doublets (ArchR Identified)
1 - Dendritic cell
2 - CD14 Mono1
3 - CD14 Mono2
4 - CD14 Mono3
5 - CD16 Mono
6 - Pre-B
7 - Pro-B
8 - CD4 Naive1
9 - CD4 Naive2
10 - CD4 Mem
11 - CD8 Naive
12 - CD8 EffMem
13 - CD8 CenMem
14 - NK
15 - Double Negative
16 - pDC
17 - Other
log10(Nuclear Fragments)
3.25
4.50

Supplementary Fig. 4 – Comparison of clustering results in scATAC-seq data derived from PBMCs.

a. UMAPs of scATAC-seq data from ~30,000 cells from the PBMCs dataset to compare clustering results across ArchR with doublet removal, ArchR without doublet removal, Signac, SnapATAC, and SnapATAC with estimated LDM. Each UMAP is colored by (left) sample, (middle) clusters as defined by ArchR with doublet removal, and (right) the number of unique nuclear fragments.

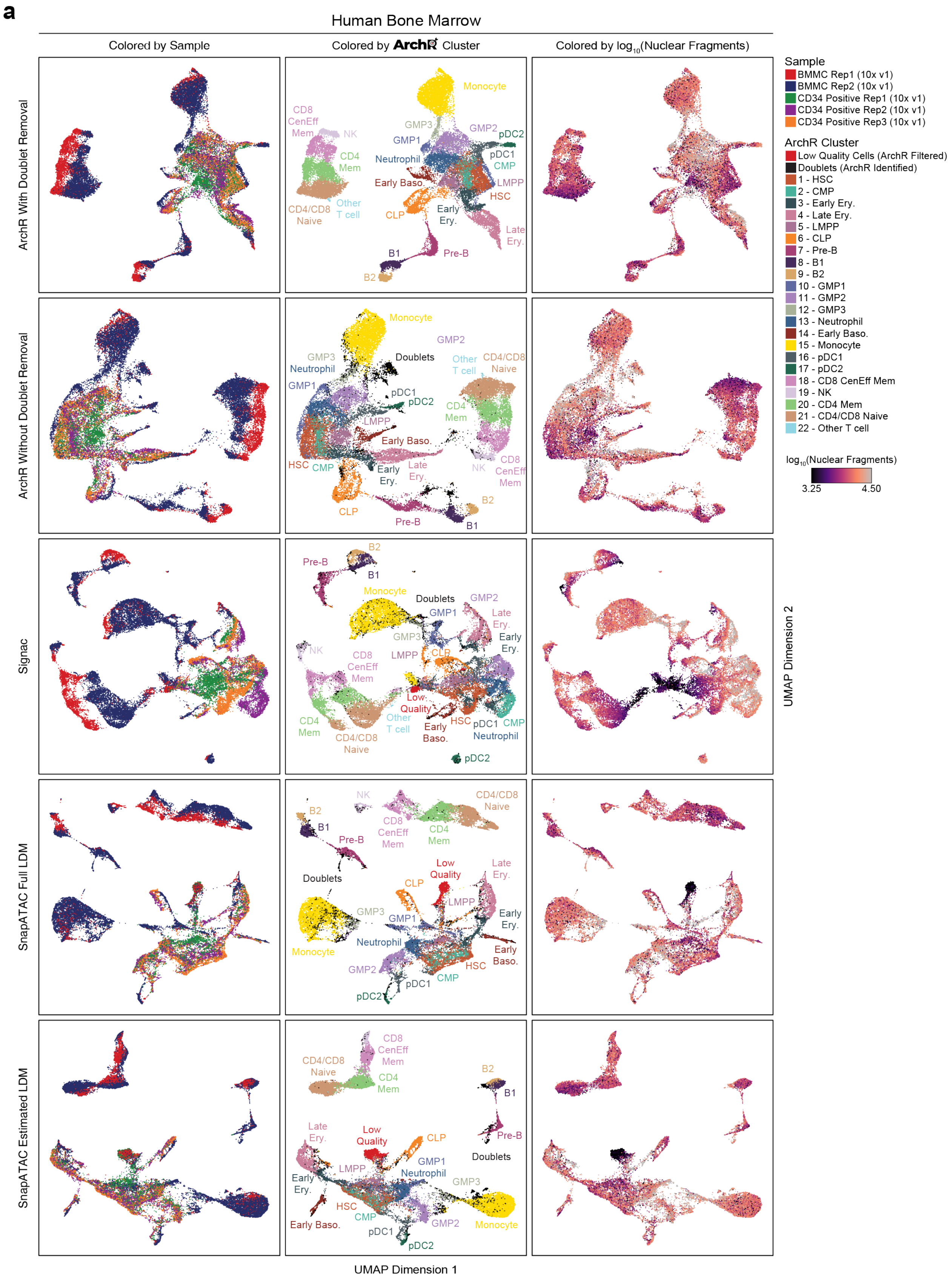
a
Human Bone Marrow
Colored by Sample
Colored by ArchR Cluster
Colored by log10(Nuclear Fragments)
ArchR With Doublet Removal
ArchR Without Doublet Removal
Signac
SnapATAC Full LDM
SnapATAC Estimated LDM
UMAP Dimension 1
UMAP Dimension 2
Monocyte
CD8 CenEff Mem
NK
CD4 Mem
CD4/CD8 Naive
Other T cell
GMP1
GMP2
GMP3
Neutrophil
Early Baso.
pDC1
pDC2
CMP
LMPP
HSC
CLP
Early Ery.
Late Ery.
B1
B2
Pre-B
Doublets
Low Quality
Sample
BMMC Rep1 (10x v1)
BMMC Rep2 (10x v1)
CD34 Positive Rep1 (10x v1)
CD34 Positive Rep2 (10x v1)
CD34 Positive Rep3 (10x v1)
ArchR Cluster
Low Quality Cells (ArchR Filtered)
Doublets (ArchR Identified)
1 - HSC
2 - CMP
3 - Early Ery.
4 - Late Ery.
5 - LMPP
6 - CLP
7 - Pre-B
8 - B1
9 - B2
10 - GMP1
11 - GMP2
12 - GMP3
13 - Neutrophil
14 - Early Baso.
15 - Monocyte
16 - pDC1
17 - pDC2
18 - CD8 CenEff Mem
19 - NK
20 - CD4 Mem
21 - CD4/CD8 Naive
22 - Other T cell
log10(Nuclear Fragments)
3.25
4.50

Supplementary Fig. 5 – Comparison of clustering results in scATAC-seq data derived from bone marrow cells.
a. UMAPs of scATAC-seq data from ~30,000 cells from the bone marrow dataset to compare clustering results across ArchR with doublet removal, ArchR without doublet removal, Signac, SnapATAC, and SnapATAC with estimated LDM. Each UMAP is colored by (left) sample, (middle) clusters as defined by ArchR with doublet removal, and (right) the number of unique nuclear fragments.

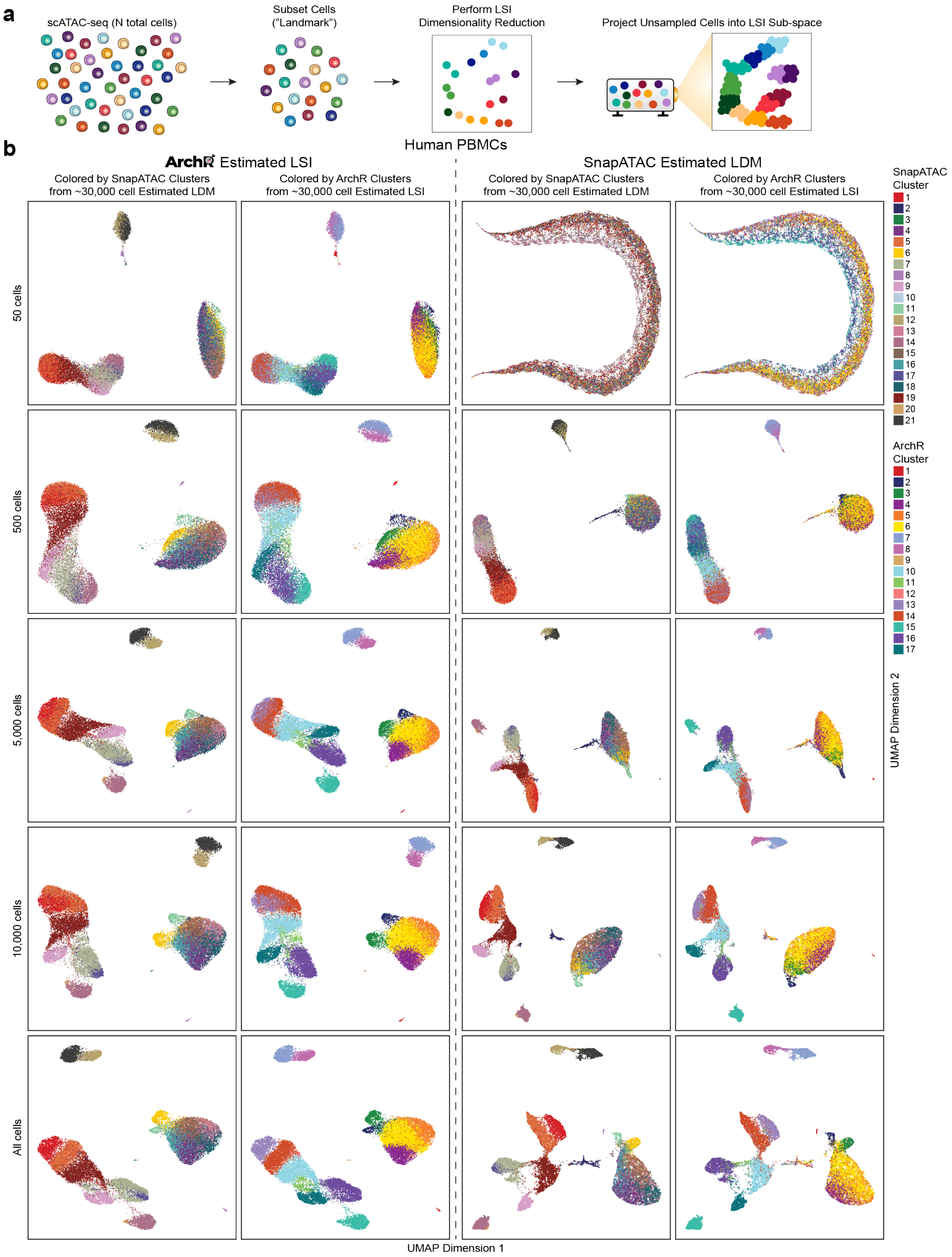
a
scATAC-seq (N total cells)
Subset Cells ("Landmark")
Perform LSI Dimensionality Reduction
Project Unsampled Cells into LSI Sub-space
b
Human PBMCs
ArchR Estimated LSI
SnapATAC Estimated LDM
Colored by SnapATAC Clusters from ~30,000 cell Estimated LDM
Colored by ArchR Clusters from ~30,000 cell Estimated LSI
Colored by SnapATAC Clusters from ~30,000 cell Estimated LDM
Colored by ArchR Clusters from ~30,000 cell Estimated LSI
50 cells
500 cells
5,000 cells
10,000 cells
All cells
SnapATAC Cluster
1 2 3 4 5 6 7 8 9 10 11 12 13 14 15 16 17 18 19 20 21
ArchR Cluster
1 2 3 4 5 6 7 8 9 10 11 12 13 14 15 16 17
UMAP Dimension 2
UMAP Dimension 1

Supplementary Fig. 6 – Comparison of estimated LSI in ArchR and estimated LDM in SnapATAC in PBMCs.

a. Schematic of the estimated LSI framework implemented by ArchR. Briefly, a subset of cells, referred to as "landmark" cells, are used for LSI dimensionality reduction. The remaining cells are then linearly projected with LSI projection into this landmark-defined LSI subspace. This method enables massive-scale analysis of scATAC-seq data with ArchR.

b. UMAPs of scATAC-seq data from ~30,000 cells from the PBMCs dataset showing the results of dimensionality reduction from (left) estimated LSI with ArchR after doublet removal or (right) estimated LDM with SnapATAC. For each analytical case, a range of cell numbers is used for the landmark cell subset (top to bottom). Within each analytical case, two UMAPs are presented, colored by the clusters identified without estimation from (left) ArchR or (right) SnapATAC.

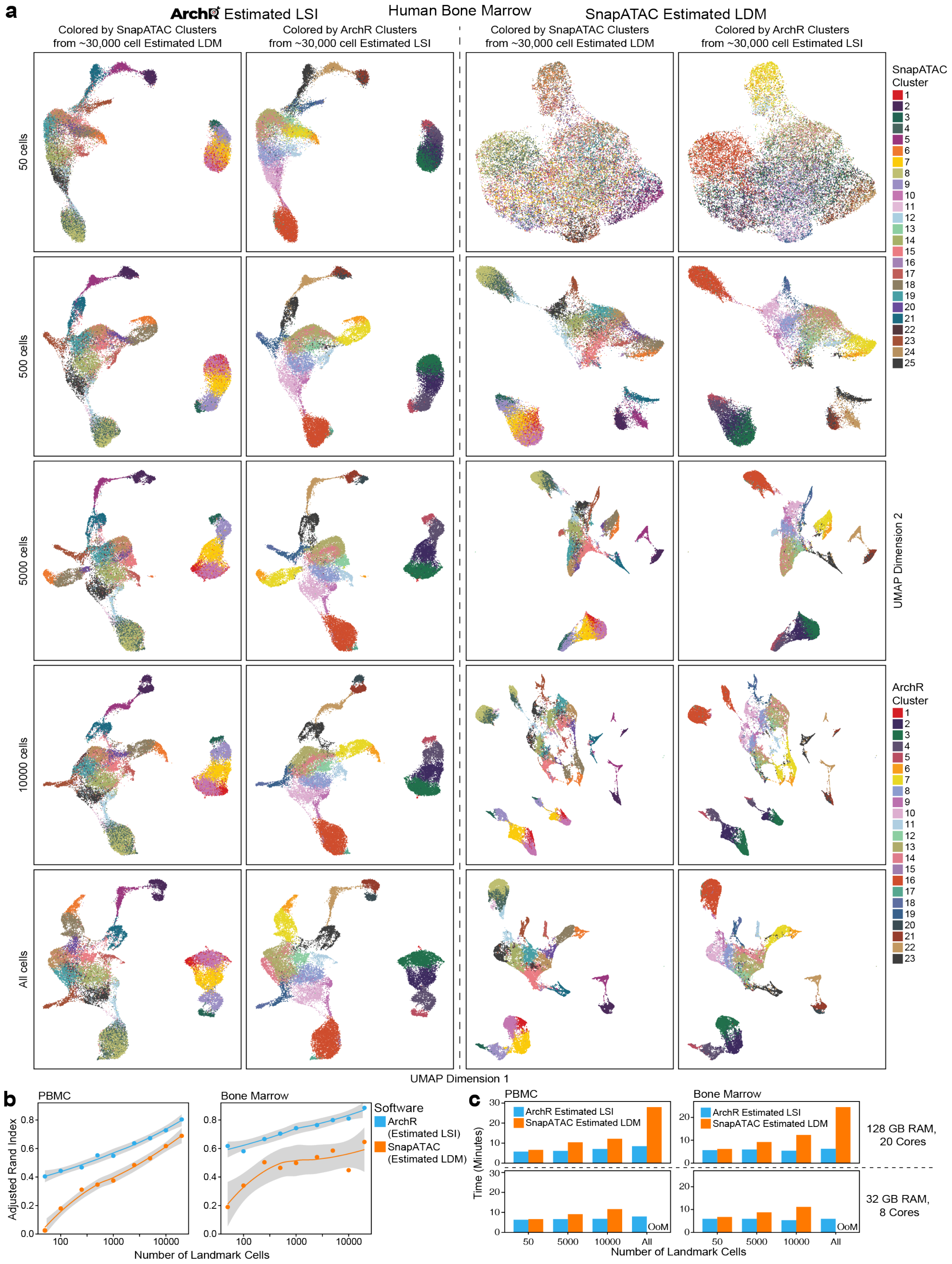
a
ArchR Estimated LSI
Human Bone Marrow
SnapATAC Estimated LDM
Colored by SnapATAC Clusters from ~30,000 cell Estimated LDM
Colored by ArchR Clusters from ~30,000 cell Estimated LSI
Colored by SnapATAC Clusters from ~30,000 cell Estimated LDM
Colored by ArchR Clusters from ~30,000 cell Estimated LSI
50 cells
500 cells
5000 cells
10000 cells
All cells
SnapATAC Cluster
ArchR Cluster
UMAP Dimension 2
UMAP Dimension 1
b
PBMC
Bone Marrow
Adjusted Rand Index
Number of Landmark Cells
Software
ArchR (Estimated LSI)
SnapATAC (Estimated LDM)
c
PBMC
Bone Marrow
ArchR Estimated LSI
SnapATAC Estimated LDM
Time (Minutes)
OoM
128 GB RAM, 20 Cores
32 GB RAM, 8 Cores
Number of Landmark Cells

Supplementary Fig. 7 – Comparison of estimated LSI in ArchR and estimated LDM in SnapATAC in bone marrow cells.
a. UMAPs of scATAC-seq data from ~30,000 cells from the bone marrow cell dataset showing the results of dimensionality reduction from (left) estimated LSI with ArchR after doublet removal or (right) estimated LDM with SnapATAC. For each analytical case, a range of cell numbers is used for the landmark cell subset (top to bottom). Within each analytical case, two UMAPs are presented, colored by the clusters identified without estimation from (left) ArchR or (right) SnapATAC.
b. Comparison of clustering fidelity based on adjusted Rand index in ArchR by estimated LSI or in SnapATAC by estimated LDM across multiple landmark subset sizes. The smooth line represents a LOESS fit (shading represents 95% confidence interval).
c. Benchmarking of run time for ArchR estimated LSI and SnapATAC estimated LDM for ~30,000 cells from (left) the PBMCs dataset and (right) the bone marrow cell dataset for (top) 128 GB of RAM with 20 cores and (bottom) 32 GB of RAM with 8 cores.

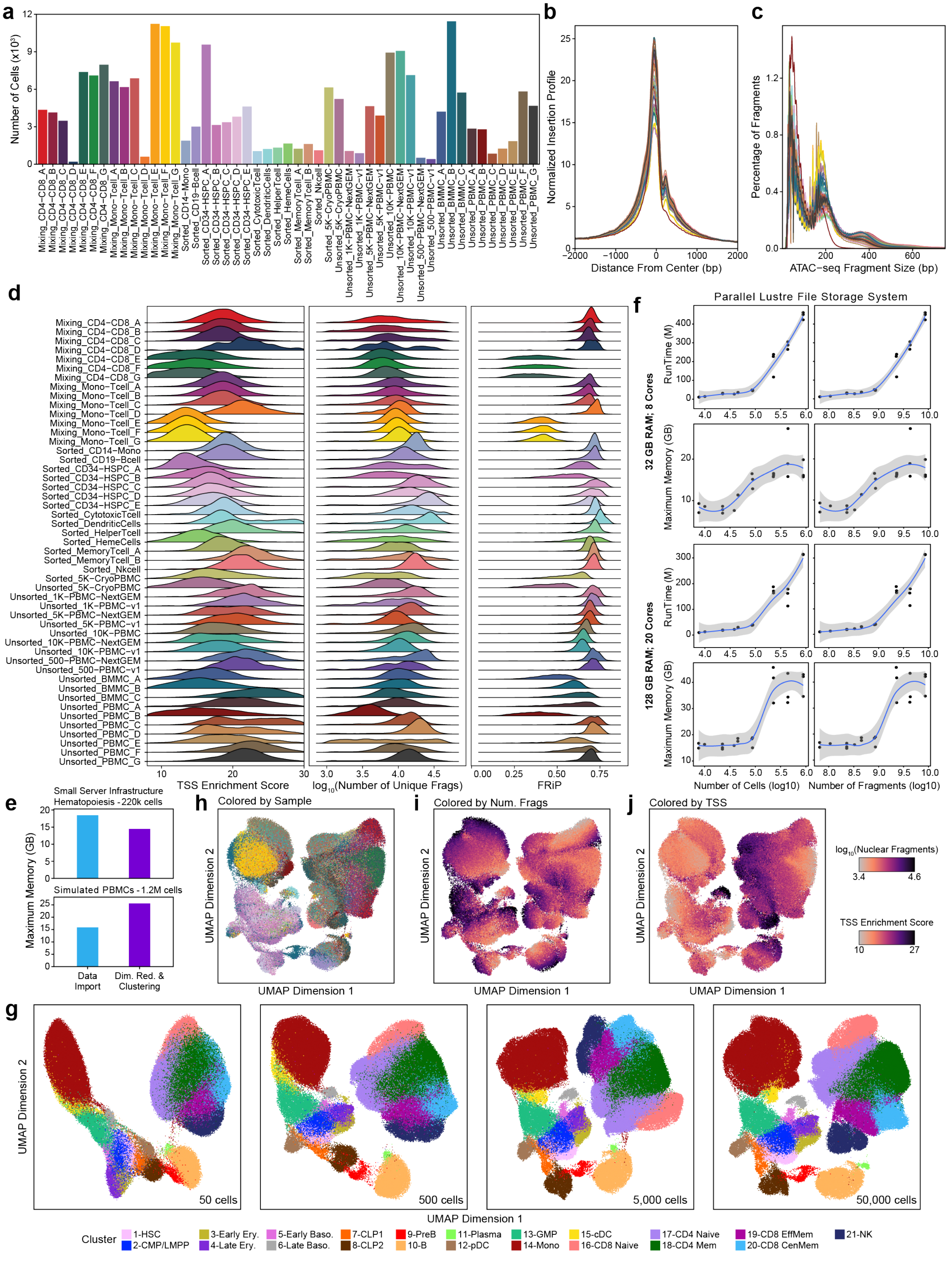
a
Number of Cells (x10³)
b
Normalized Insertion Profile
Distance From Center (bp)
c
Percentage of Fragments
ATAC−seq Fragment Size (bp)
d
TSS Enrichment Score
log10(Number of Unique Frags)
FRiP
f
Parallel Lustre File Storage System
32 GB RAM; 8 Cores
128 GB RAM; 20 Cores
RunTime (M)
Maximum Memory (GB)
Number of Cells (log10)
Number of Fragments (log10)
e
Small Server Infrastructure
Hematopoiesis - 220k cells
Simulated PBMCs - 1.2M cells
Maximum Memory (GB)
Data Import
Dim. Red. & Clustering
h
Colored by Sample
i
Colored by Num. Frags
j
Colored by TSS
UMAP Dimension 2
UMAP Dimension 1
log10(Nuclear Fragments)
TSS Enrichment Score
g
50 cells
500 cells
5,000 cells
50,000 cells
Cluster
1-HSC
2-CMP/LMPP
3-Early Ery.
4-Late Ery.
5-Early Baso.
6-Late Baso.
7-CLP1
8-CLP2
9-PreB
10-B
11-Plasma
12-pDC
13-GMP
14-Mono
15-cDC
16-CD8 Naive
17-CD4 Naive
18-CD4 Mem
19-CD8 EffMem
20-CD8 CenMem
21-NK

Supplementary Fig. 8 – Quality control of the large hematopoiesis dataset.
a. Bar plot showing the number of cells passing ArchR QC thresholds from each of the immune cell scATAC-seq datasets used for the ~220k cell hematopoiesis dataset.
b-c. Aggregate (**b**) TSS insertion profiles centered at all TSS regions or (**c**) fragment size distributions for the cells passing ArchR QC thresholds for each sample in the hematopoiesis dataset. Line color represents the sample from the dataset as indicated in **Supplementary Fig. 8a**.
d. Summary of quality control information for each cell from the hematopoiesis dataset. The distribution of (left) TSS enrichment scores, (middle) the number of unique nuclear fragments, and (right) the fraction of reads in peak regions (FRiP) are shown for each single cell passing filter.
e. Benchmarking of peak memory usage for analysis of (top) the ~220,000 cells from the hematopoiesis dataset and (bottom) ~1,200,000 simulated PBMCs using a computational infrastructure with 32 GB of RAM and 8 cores with an HP Lustre file storage system.
f. Benchmarking of (top) run time in minutes and (bottom) peak memory usage in GB for datasets ranging from (left) 10,000 to 1,000,000 cells or (right) 100,000,000 to 10,000,000,000 fragments. This analysis was performed using a computational infrastructure with (top) 32 GB of RAM and 8 cores and (bottom) 128 GB and 20 cores with a parallel Lustre file storage system. The smooth line represents a LOESS fit (shading represents 95% confidence interval).
g. UMAPs of scATAC-seq data derived from estimated LSI of the hematopoiesis dataset using different numbers of landmark cells. These UMAPs are colored by the clusters identified from the 25,000-cell estimated LSI shown in **Figure 3b**.
h-j. UMAPs of scATAC-seq data as shown in **Figure 3b**, colored by (**h**) the different experimental samples (as shown in **Supplementary Fig. 8a**), (**i**) the number of unique nuclear fragments, or (**j**) the per-cell TSS enrichment score.

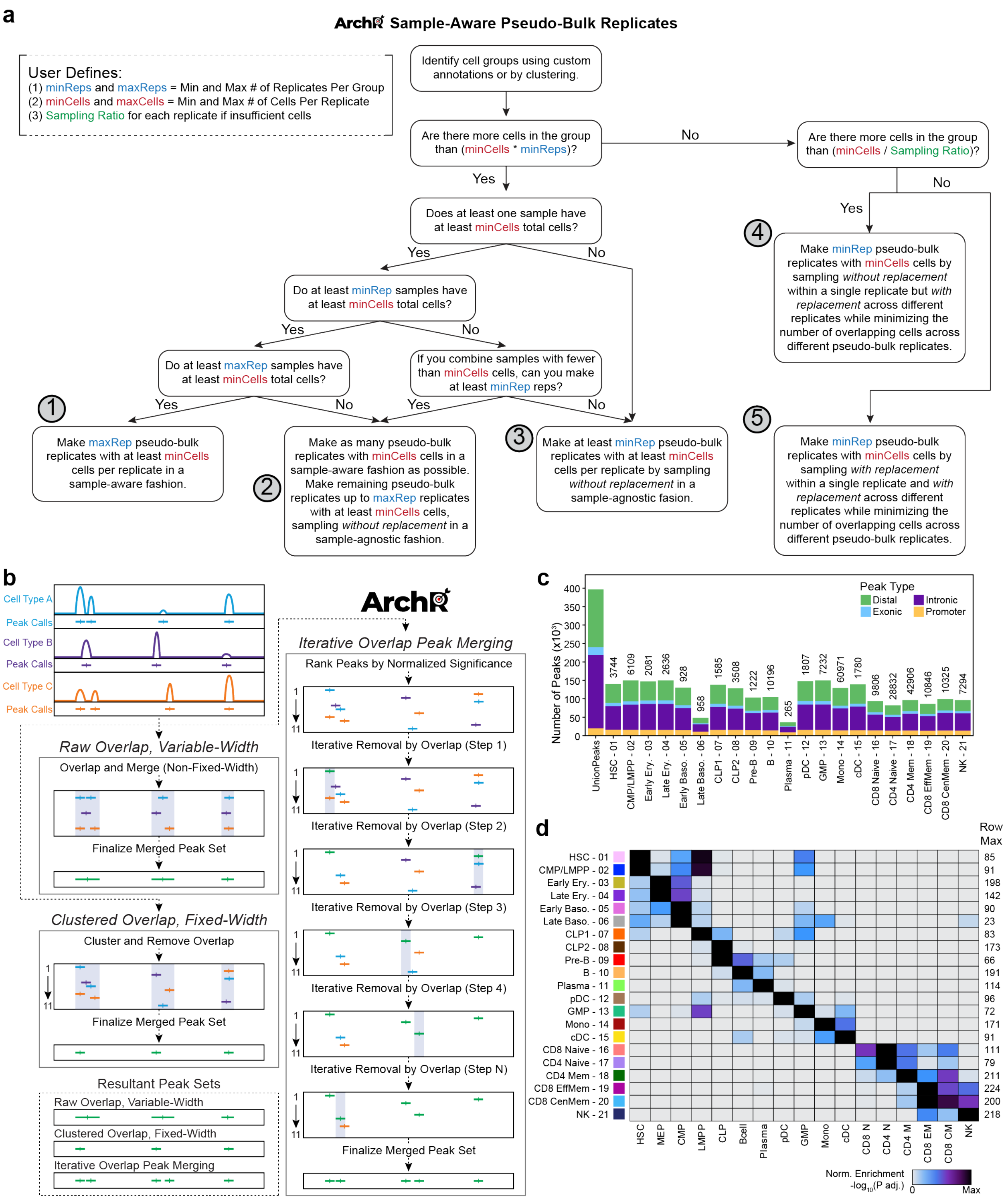

Supplementary Fig. 9 – Pseudo-bulk replicate generation and peak calling in ArchR.
a. Schematic for the generation of sample-aware pseudo-bulk replicates in ArchR for downstream analyses. Briefly, for each cell grouping (in most cases identified by clusters), cells are split per sample of origin. Next, for each cell grouping these sample-aware cell groups are tested for being larger than a specified minimum number of cells to create a specified minimum number of sample-

aware replicates. If these requirements are not met with a simple splitting, ArchR accounts for each different case by using sub-sampling approaches.

b. Schematic for iterative overlap peak merging in ArchR to identify non-overlapping fixed-width peaks. Briefly, peaks (peak summits that are extended to yield fixed-width peaks) are called per sample and then ranked by significance. Next, for all peaks across multiple samples, the peak with the highest significance is kept. Peaks directly overlapping this most-significant peak are discarded and then this procedure is repeated until all peaks have either been kept or discarded, thus converging upon a non-overlapping fixed-width peak set.

c. Bar plot showing the number of final peaks identified across all clusters ("Union Peaks") and within each cluster from the hematopoiesis dataset. Bars are colored by peak annotation relative to a supplied gene set.

d. Heatmap of hypergeometric enrichment testing the overlap of curated cell type-specific peak sets (peaks with higher chromatin accessibility vs the average accessibility of the other bulk ATAC-seq profiles) with the marker peak sets identified for each cluster in the hematopoiesis dataset in **Figure 3c**.

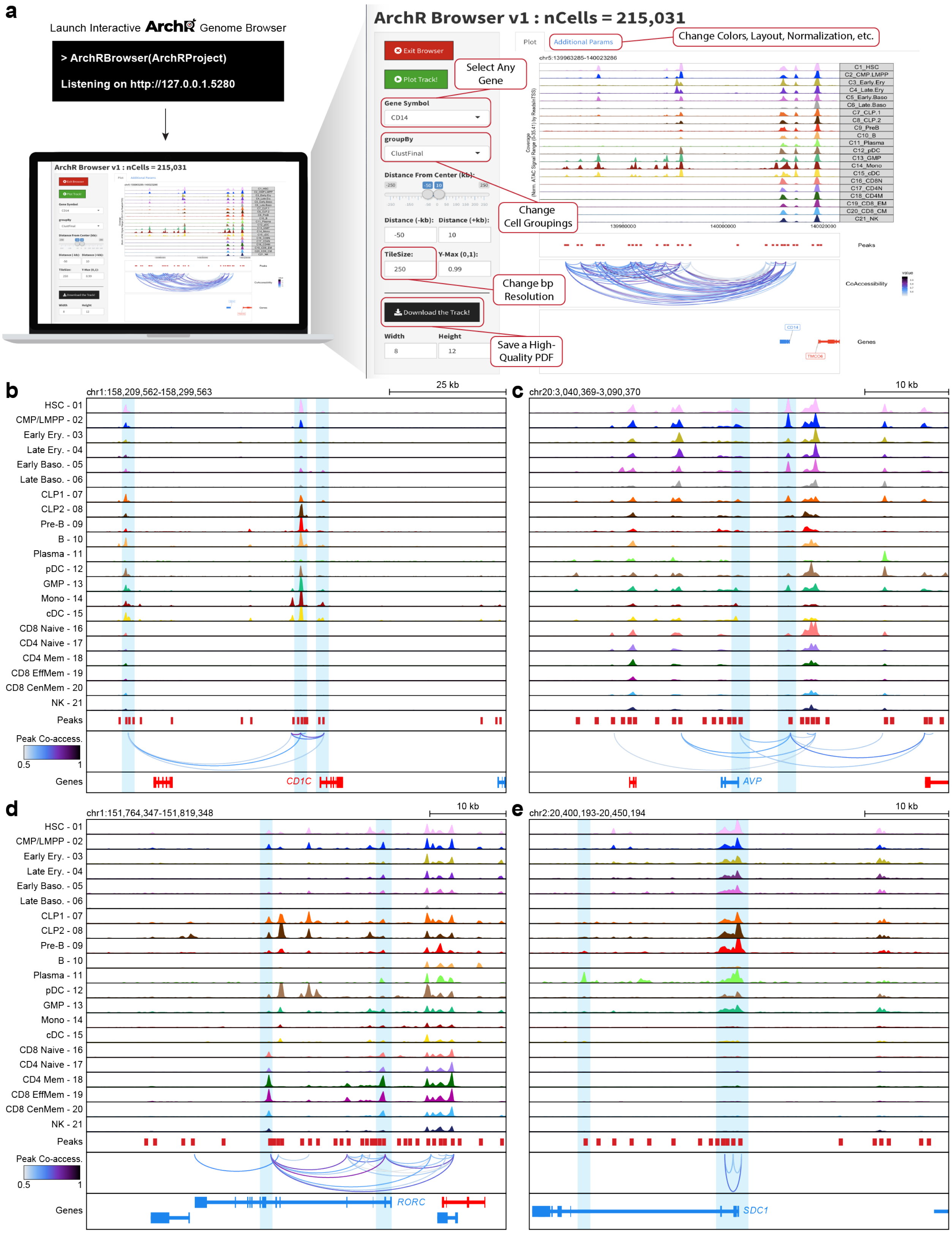
a
Launch Interactive ArchR Genome Browser
> ArchRBrowser(ArchRProject)
Listening on http://127.0.0.1.5280
ArchR Browser v1 : nCells = 215,031
Exit Browser
Plot Track!
Select Any Gene
Gene Symbol
CD14
groupBy
ClustFinal
Distance From Center (kb):
Distance (-kb):
Distance (+kb):
TileSize:
Y-Max (0,1):
Change bp Resolution
Download the Track!
Width
Height
Save a High-Quality PDF
Plot
Additional Params
Change Colors, Layout, Normalization, etc.
Change Cell Groupings
Peaks
CoAccessibility
Genes
b
chr1:158,209,562-158,299,563
25 kb
HSC - 01
CMP/LMPP - 02
Early Ery. - 03
Late Ery. - 04
Early Baso. - 05
Late Baso. - 06
CLP1 - 07
CLP2 - 08
Pre-B - 09
B - 10
Plasma - 11
pDC - 12
GMP - 13
Mono - 14
cDC - 15
CD8 Naive - 16
CD4 Naive - 17
CD4 Mem - 18
CD8 EffMem - 19
CD8 CenMem - 20
NK - 21
Peaks
Peak Co-access.
0.5
1
Genes
CD1C
c
chr20:3,040,369-3,090,370
10 kb
AVP
d
chr1:151,764,347-151,819,348
10 kb
RORC
e
chr2:20,400,193-20,450,194
10 kb
SDC1

Supplementary Fig. 10 – The interactive ArchR genome browser facilitates visualization of track-level data in real time.

a. Schematic of the ArchR integrative genome browser. Briefly, the ArchR integrative browser is launched with a single command into an interactive Shiny session. From there, users can select any gene to visualize the accessibility genome track. Additionally, users can change cell groupings, resolution, layout and more with an intuitive user interface. Lastly, users can supply custom feature regions (such as peak sets) or looping/linkage sets (such as peak co-accessibility).

b-e. Genome accessibility track visualization of marker genes with peak co-accessibility for (**b**) the *CD1C* locus (chr1:158,209,562-158,299,563), (**c**) the *AVP* locus (chr20:3,040,369-3,090,370), (**d**) the *RORC* locus (chr1:151,764,347-151,819,348), and (**e**) the *SDC1* locus (chr2:20,400,193-20,450,194).

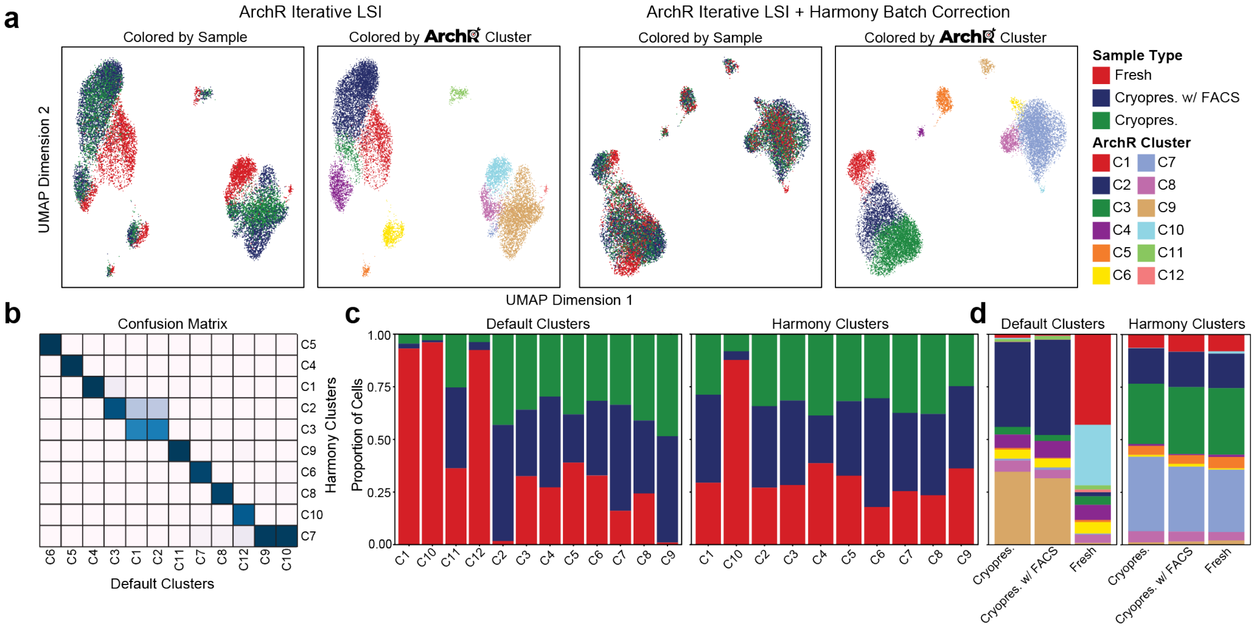

Supplementary Fig. 11 – Harmony-based batch correction reduces biases across scATAC-seq data from fresh and cryopreserved PBMCs

a. (left) UMAP of ArchR iterative LSI and (right) ArchR iterative LSI with Harmony-based batch correction for PBMCs that were assayed fresh (Fresh), after cryopreservation (Cryopres.), or after cryopreservation and subsequent fluorescence-based sorting for viable cells (Cryopres. w/ FACS).

b. Confusion matrix of clusters identified by default and with Harmony-based batch correction.

c. Barplot showing the proportion of cells belonging to each sample within each cluster for clusters identified by (left) default and (right) with Harmony-based batch correction.

d. Barplot showing the proportion of cells belonging to each cluster within each sample for clusters identified by (left) default and (right) with Harmony-based batch correction.

SUPPLEMENTARY METHODS

ArchR Methods – Preface

All ArchR features were carefully designed and optimized to enable analysis of 250,000 cells or greater on a minimal computing environment in R. All ArchR HDF5-formatted processing was performed with the Bioconductor[46] package "rhdf5" (https://www.bioconductor.org/packages/release/bioc/html/rhdf5.html). All ArchR genomic coordinate operations were performed with the Bioconductor package "GenomicRanges" (https://bioconductor.org/packages/release/bioc/html/GenomicRanges.html) and "IRanges" (https://bioconductor.org/packages/release/bioc/html/IRanges.html).

ArchR Methods – scATAC Definitions

Fragments – In ATAC-seq data analysis, a "fragment" refers to a sequenceable DNA molecule created by two transposition events. Each end of that fragment is sequenced using paired-end sequencing. The inferred single-base position of the start and end of the fragment is adjusted based on the insertion offset of Tn5. As reported previously[47], Tn5 transposase binds to DNA as a homodimer with 9-bp of DNA between the two Tn5 molecules. Because of this interaction, each Tn5 homodimer binding event creates two insertions, separated by 9 bp. Thus, the actual central point of the "accessible" site is in the very center of the Tn5 dimer, not the location of each Tn5 insertion. To account for this, we apply an offset to the individual Tn5 insertions, adjusting plus-stranded insertion events by +4 bp and minus-stranded insertion events by -5 bp. This offset is consistent with the convention put forth during the original description of ATAC-seq[48]. Thus, in ArchR, "fragments" refers to a table or Genomic Ranges object containing the chromosome, offset-adjusted chromosome start position, offset-adjusted chromosome end position, and unique cellular barcode ID corresponding to each sequenced fragment.

Tn5 insertions – In ArchR, "insertions" refers to the offset-adjusted single-base position of Tn5 insertion on either end of the fragment. Insertion positions are accessed in ArchR primarily using resize(fragments, 1, "start") and resize(fragments, 1, "end"). See the description of "fragments" above for a detailed description of Tn5 insertion offsets.

Counting Accessibility – In ArchR, "counting accessibility" refers to counting the number of Tn5 insertions observed within each described feature.

TSS enrichment score – In ArchR, the "TSS enrichment" refers to the relative enrichment of Tn5 insertions at gene TSS sites genome-wide compared to a local background. This represents a measure of signal-to-background in ATAC-seq data. See below for how TSS enrichment is calculated in ArchR. In this work, the TSS enrichment score from ArchR is based on the TSS regions defined by the TxDb.Hsapiens.UCSC.hg19.knownGene (or TxDb.Mmusculus.UCSC.mm9.knownGene for the Mouse Atlas) transcript database object.

ArchR Methods – Arrow Files and ArchRProject

The base unit of an analytical project in ArchR is called an "Arrow file". Each Arrow file stores all of the data associated with an individual sample (i.e. metadata, accessible fragments, and data matrices). Here, an "individual sample" would be the most detailed unit of analysis desired (for

ex. a single replicate of a particular condition). During creation and as additional analyses are performed, ArchR updates and edits each Arrow file to contain additional layers of information. It is worth noting that, to ArchR, an Arrow file is actually just a path to an external file stored on disk. More explicitly, an Arrow file is not an R-language object that is stored in memory but rather an HDF5-format file stored on disk. Because of this, we use an “ArchRProject” object to associate these Arrow files together into a single analytical framework that can be rapidly accessed in R. This ArchRProject object is small in size and is stored in memory.

Certain actions can be taken directly on Arrow files while other actions are taken on an ArchRProject which in turn updates each associated Arrow file. Because Arrow files are stored as large HDF5-format files, "get-er" functions in ArchR retrieve data by interacting with the ArchRProject while "add-er" functions either (i) add data directly to Arrow files, (ii) add data directly to an ArchRProject, or (iii) add data to Arrow files by interacting with an ArchRProject.

ArchR Methods – Reading Input Data into an Arrow File

ArchR can utilize multiple input formats of scATAC-seq data which is most frequently in the format of fragment files and BAM files. Fragment files are tabix-sorted text files containing each scATAC-seq fragment and the corresponding cell ID, one fragment per line. BAM files are binarized tabix-sorted files that contain each scATAC-seq fragment, raw sequence, cellular barcode id and other information. The input format used will depend on the pre-processing pipeline used. At the time of publication, ArchR does not provide support for alignment of raw sequence data in the format of fastq files. Thus, pre-processing / alignment is required prior to using ArchR. However, once aligned, ArchR supports both fragment files and BAM files as input. If you generate scATAC-seq data using the 10x Genomics Chromium platform, we recommend using the Cell Ranger software for alignment and generation of fragment files which can be used directly as input to ArchR. If you generate scATAC-seq data using the Bio-Rad SureCell platform, we recommend bap (https://github.com/caleblareau/bap)[8]. If you generated scATAC-seq data using sci-ATAC-seq, we recommend either bap (https://github.com/caleblareau/bap) or the pipeline associated with the fly scATAC-seq atlas (https://github.com/shendurelab/fly-atac)[3]. Given a specified genome annotation (ArchR has pre-loaded genome annotations for mm9, mm10, hg19, and hg38 and additional genomes can be added manually), ArchR reads these input files in sub-chromosomal chunks using Rsamtools. ArchR uses “scanTabix” to read fragment files and “scanBam” to read BAM files. During this input process, each input chunk is converted into a compressed table-based representation of fragments containing each fragment chromosome, offset-adjusted chromosome start position, offset-adjusted chromosome end position and cellular barcode ID. These chunk-wise fragments are then stored in a temporary HDF5-formatted file to preserve memory usage while maintaining rapid access to each chunk. Finally, all chunks associated with each chromosome are read, organized, and re-written to an “Arrow file” within a single HDF5 group called “fragments”. This pre-chunking procedure enables ArchR to process extremely large input files efficiently and with low memory usage, enabling full utilization of parallel processing (**Supplementary Fig. 1a**).

ArchR Methods – QC Based on TSS Enrichment and Unique Nuclear Fragments

Strict quality control (QC) of scATAC-seq data is essential to remove the contribution of low-quality cells. In ArchR, one characteristic of “low-quality” is a low signal-to-background ratio, which is often attributed to dead or dying cells which have de-chromatinzed DNA which allows for random transposition genome-wide. Traditional bulk ATAC-seq analysis has used the TSS

enrichment score as part of a standard workflow (https://www.encodeproject.org/atac-seq/) for determination of signal-to-background. We and others have found the TSS enrichment to be representative across the majority of cell types tested in both bulk ATAC-seq and scATAC-seq. The idea behind the TSS enrichment score metric is that ATAC-seq data is universally enriched at gene TSS regions compared to other genomic regions. By looking at per-base-pair accessibility centered at these TSS regions, we see a local enrichment relative to flanking regions (1900-2000 bp distal in both directions). The ratio between the peak of this enrichment (centered at the TSS) relative to these flanking regions represents the TSS enrichment score. Traditionally, the per-base-pair accessibility is computed for each bulk ATAC-seq sample and then this profile is used to determine the TSS enrichment score. Performing this operation on a per-cell basis in scATAC-seq is relatively slow and computationally expensive. To accurately approximate the TSS enrichment score per single cell, we count the average accessibility within a 50-bp region centered at each single-base TSS position and divide this by the average accessibility of the TSS flanking positions (+/- 1900 – 2000 bp). This approximation was highly correlated ($R > 0.99$) with the original method and values were extremely close in magnitude. By default in ArchR (and throughout this manuscript), pass-filter cells are identified as those cells having a TSS enrichment score greater than 4 and more than 1000 unique nuclear fragments (i.e those fragments that do not map to chrM). We require pass-filter cells to have at least 1000 unique nuclear fragments to ensure that each single cell has enough associated data to perform meaningful computation. Moreover, droplets with fewer than 1000 unique nuclear fragments may not have contained full cells but rather fragmented chromosomes or free-floating DNA.

ArchR Methods – Tile Matrix

Traditional bulk ATAC-seq analysis relies on the creation of a peak matrix from a peak-set encompassing the precise accessible regions across all samples. This peak set, and thus the resulting peak matrix, is specific to the samples used in the analysis and must be re-generated when new samples are added. Moreover, identification of peaks from scATAC-seq data would optimally be performed after clusters were identified to ensure that cluster-specific peaks are captured. Thus, the optimal solution for scATAC-seq would be to identify an unbiased and consistent way to perform analysis prior to cluster identification, without the need for calling peaks. To circumvent the requirement for calling peaks prior to cluster identification, others have tiled the genome into fixed non-overlapping tiled windows. This method additionally benefits from being stable across samples and the tiled regions do not change based on inclusion of additional samples. However, these tiled windows are usually greater than or equal to 5 kb in length, which is more than 10-fold greater than the size of typical accessible regions containing TF binding sites[24–26]. For this reason, ArchR uses 500-bp genome-wide tiled windows for all analysis upstream of cluster identification. To create a tile matrix, ArchR reads in the scATAC-seq fragments for a chromosome and converts these to insertions. ArchR then floors these insertions to the nearest tile region with floor(insertion / tileSize) + 1. The tile regions and cell barcode id (as an integer) are then used as input for Matrix::sparseMatrix which tallies the number of input rows (tiles, denoted as i) and columns (cells, denoted as j) and creates a sparseMatrix. Blacklisted regions, regions that have "anomalous, unstructured, or high signal in next-generation sequencing experiments independent of cell line or experiment"[49], are excluded from this tile matrix. This analysis is performed for each chromosome and stored in the corresponding Arrow file. This fast and efficient conversion of scATAC-seq fragments to a tile matrix, without computing genomic overlaps, facilitates efficient construction of 500-bp tile matrices for analyses.

ArchR Methods – Gene Score Matrix

ArchR facilitates the inference of gene expression from chromatin accessibility (called "gene scores") by using custom distance-weighted accessibility models. For each chromosome, ArchR creates a tile matrix (user-defined tile size that is not pre-computed, default is 500 bp), overlaps these tiles with the gene window (user-defined, default is 100 kb), and then computes the distance from each tile (start or end) to the gene body (with optional extensions upstream or downstream) or gene start. We have found that the best predictor of gene expression is the local accessibility of the gene region which includes the promoter and gene body (**Extended Data Fig. 6-7**). To properly account for distal accessibility, for each gene ArchR identifies the subset of tiles that are within the gene window and do not cross another gene region. This filtering allows for inclusion of distal regulatory elements that could improve the accuracy of predicting gene expression values but excludes regulatory elements more likely to be associated with another gene (for ex. the promoter of a nearby gene). The distance from each tile to the gene is then converted to a distance weight using a user-defined accessibility model (default is $e^{(-abs(distance)/5000)} + e^{-1}$). When the gene body is included in the gene region (where the distance-based weight is the maximum weight possible), we found that extremely large genes can bias the overall gene scores. In these cases, the total gene scores can vary substantially due to the inclusion of insertions in both introns and exons. To help adjust for these large differences in gene size, ArchR applies a separate weight for the inverse of the gene size (1 / gene size) and scales this inverse weight linearly from 1 to a hard max (which can be user-defined, with a default of 5). Smaller genes thus receive larger relative weights, partially normalizing this length effect. The corresponding distance and gene size weights are then multiplied by the number of Tn5 insertions within each tile and summed across all tiles within the gene window (while still accounting for nearby gene regions as described above). This summed accessibility is a "gene score" and is depth normalized across all genes to a constant (user-defined, default of 10,000). Computed gene scores are then stored in the corresponding Arrow file for downstream analyses.

In this manuscript, we describe substantial efforts to identify optimal models to infer gene expression from chromatin accessibility. It is important to note that all of these models were assessed using data from hematopoietic cell types and thus may not apply equivalently across all cell types. This is part of the justification for enabling custom user-defined models.

ArchR Methods – Paired scRNA-seq Gene Expression Matrix

ArchR can perform paired analyses on multiomic scATAC-seq and scRNA-seq data by reading in a gene expression matrix that has been converted into a SummarizedExperiment object in R. For the 10x Multiome data, ArchR can read the 10x scRNA-seq matrix into R using the "feature_bc_matrix.h5" file and the "import10xFeatureMatrix" function with featureType = "Gene Expression". This SummarizedExperiment can then be added to the corresponding Arrow file using "addGeneExpressionMatrix" (from either the Arrow file or ArchRProject as input) which will (1) subset by cells overlapping the Arrow file, (2) record the number of genes detected per cell (nGenes), (3) record the number of unique molecular identifiers per cell (nUMI), (4) filter out genes that are not within your standard chromosomes, (5) depth normalize and (6) add the matrix to the Arrow file. For cells that have a scATAC-seq measurement but not paired scRNA-seq nUMIs and nGenes will be recorded as "NA" for both. We then filter these cells without paired measurements when constructing an ArchR project.

ArchR Methods – Iterative LSI Procedure

The default LSI implementation in ArchR is conceptually similar to the method introduced in Signac (https://satijalab.org/signac/), which, for a cell x features matrix (typically tiles or peaks), uses a term frequency (column sums) that has been depth normalized to a constant (10,000) followed by normalization with the inverse document frequency (1 / row sums) and then log-transformed (aka log(TF-IDF)). This normalized matrix is then factorized by singular value decomposition (SVD) and then standardized across the reduced dimensions for each cell via z-score. To control for strong biases related to the number of fragments ArchR enables outlier removal (user-defined, default bottom 2% and top 98%), where outlier cells are withheld from the LSI dimensionality reduction and later projected into this subspace. We have found this procedure reduces spurious small clusters of aggregates, often biased by technical covariates such as number of fragments per cell, while maintaining lowly represented biological clusters. ArchR additionally allows for the use of alternative LSI implementations based on previously published scATAC-seq papers[5–7]. As mentioned above, the input to LSI-based dimensionality reduction is the genome-wide 500-bp tile matrix.

In scRNA-seq, identifying variable genes is a common way to compute dimensionality reduction (such as PCA), as these highly variable genes are more likely to be biologically important, and focusing on these genes likely reduces low-level contributions of variance potentially due to experimental noise. ScATAC-seq data is binary, precluding the possibility of identifying variable peaks for dimensionality reduction. Therefore, rather than identifying the most variable peaks, we initially tried using the most accessible features as input to LSI; however, the results when running multiple samples exhibited a high degree of noise and low reproducibility. We therefore moved to our previously described "iterative LSI" approach[6,7]. This approach computes an initial LSI transformation on the most accessible tiles and identifies lower resolution clusters that are driven by clear biological differences. For example, when performed on peripheral blood mononuclear cells, this approach will identify clusters corresponding to the major cell types (T cells, B cells, and monocytes). Then ArchR computes the average accessibility for each of these clusters across all features creating "pseudo-bulks". ArchR then identifies the most variable peaks across these pseudo-bulks to use as features for the second round of LSI. In this second iteration, the selected variable peaks correspond more similarly to the variable genes used in scRNA-seq LSI implementations, insofar as they are highly variable across biologically meaningful clusters. We have found this approach can also effectively minimize batch effects and allows operations on a more reasonably sized feature matrix. Additionally, we observe that this procedure still allows the identification of rare cell types, such as plasma cells in the bone marrow cell dataset that exist at ~0.1% prevalence. For larger batch effects, ArchR enables Harmony-based batch correction on the LSI-reduced coordinates[50].

ArchR Methods – Estimated LSI Procedure

For extremely large datasets, ArchR can estimate the LSI dimensionality reduction with LSI projection. This procedure is similar to the iterative LSI workflow, however the LSI procedure differs. First, a subset of randomly selected “landmark” cells is used for LSI dimensionality reduction. Second, the remaining cells are TF-IDF normalized using the inverse document frequency determined from the landmark cells. Third, these normalized cells are projected into the SVD subspace defined by the landmark cells. This leads to an LSI transformation based on a small set of cells used as landmarks for the projection of the remaining cells. This estimated LSI procedure is efficient with ArchR because, when projecting the new cells into the landmark cells

LSI, ArchR iteratively reads in the cells from each sample and LSI projects them without storing them all in memory. This optimization leads to minimal memory usage and further increases the scalability for extremely large datasets. Even with comparatively small landmark cell subsets (500-5000 cells), we find that this procedure is able to maintain the global structure and recapitulates the clusters well; however, the required landmark set size is dependent on the proportion of different cells within the dataset.

ArchR Methods – Iterative LSI Procedure (Paired scRNA-seq)
When working with paired gene expression data, the iterative LSI procedure can be used directly on the gene expression matrix by adjusting some of the default parameters (though we note that ArchR does not currently support solely scRNA-seq analysis). First, instead of first identifying the highest expressed genes (like for scATAC-seq), ArchR identifies the top variable genes for LSI dimensionality reduction. Second, when controlling biases, the user may use the number of unique molecular identifiers (nUMI) for outlier removal as described above. The estimated iterative LSI procedure can be similarly used with these minimal changes.

ArchR Methods – Handling Strong Batch Effects
It is important to be mindful of batch effects when working with multiple samples that were assayed at different times, prepared with different protocols, or profiled using different platforms. The first step to assaying batch effects is to create a single-cell embedding (such as UMAP) and visualize this embedding with the cells colored by sample type. If one observes a strong batch effect (with the iterative LSI procedure default parameters) we first recommend trying to minimize this effect by (1) reducing the maximum number of clusters identified in the first iteration, (2) decreasing the number of variable features that may be associated with the batch effect and (3) increasing the number of iterations. If these three main steps do not removed the observed batch effects, then we recommend trying Harmony-based batch correction[50], which can be implemented directly in ArchR. It is important to note that we have found Harmony to perform better for more discrete clusters than continuous clusters. For example, when analyzing scATAC-seq data from PBMCs[6] that were either (i) assayed fresh, (ii) cryopreserved, thawed, and then assayed, or (iii) cryopreserved, thawed, sorted for viable cells, and then assayed, we found significant batch effects that required Harmony-based batch correction (**Supplementary Fig. 11a-d**). Once these noticeable batch effects are attenuated, we recommend increasing the "reproducibility" in "addReproduciblePeakSet" to ensure peaks identified for each cluster are reproducible across all sample-aware pseudobulk replicates. This reproducibility will minimize peaks that are batch related, improving the fidelity of downstream analyses. For the fresh and cryopreserved PBMCs, identifying the peaks that are consistent across both fresh and frozen conditions will minimize this batch effect in downstream analysis.

ArchR Methods – Combining Independent Dimensionality Reductions
ArchR allows for combining dimensionality reduction for paired multi-modal assays. First, the iterative LSI dimensionality reduction procedure (or other reduction) is performed on each assay independently. Second, these reductions are then scaled by the variance across all dimensions (1 / sqrt(sum(colVars(rD)), where rD is the reduced dimensions). Third, these independent reductions can be weighted (user-defined) by multiplying the scaled reductions by scalar weights. Fourth, these reductions are concatenated into a new combined reduction that can be used for downstream analyses.

ArchR Methods – Identification of Doublets

Single-cell data generated on essentially any platform are susceptible to the presence of doublets. A doublet refers to a single nano-reaction (i.e. a droplet) that received a single barcoded bead and more than one cell/nucleus. This causes the reads from more than one cell to appear as a single cell. For 10x Genomics applications, the percentage of total "cells" that are actually doublets is proportional to the number of cells loaded into the reaction. Even at lower cell loadings as recommended by standard kit use, more than 5% of the data may come from doublets, and this spurious data exerts substantial effects on clustering. This issue becomes particularly problematic in the context of developmental/trajectory data because doublets can look like a mixture between two cell types and this can be confounded with intermediate cell types or cell states.

To predict which "cells" are actually doublets in ArchR, we synthesize in silico doublets from the data by mixing the reads from thousands of combinations of individual cells. Next, we perform iterative LSI followed by UMAP for each individual sample. We then LSI project the synthetic doublets into the LSI subspace followed by UMAP projection. ArchR identifies the *k*-nearest neighbors (user-defined, default 10) to each simulated projected doublet. By iterating this procedure N times (user-defined, default 3 times the total number of cells), we can compute binomial enrichment statistics (assuming every cell could be a doublet with equal probability) for each single cell based on the presence of nearby simulated projected doublets (in the LSI or UMAP subspace defined by the user). This approach is similar to previous approaches[21,22], but differs in that LSI is used for dimensionality reduction and UMAP projection is used for identification. The number of doublets to remove is then determined based on either the number of cells that pass QC or for the approximate number of cells loaded as defined by the user. While we have optimized these parameters for general use, users should sensibly check their results with and without doublet removal. It is important to note that ArchR is not designed to identify “multiplets” – droplets with more than 2 cells/nuclei. Previous work has predicted that, at standard cell loading concentrations (~5k cells), doublets make up greater than 97% of all multi-cell droplets[22].

To evaluate the accuracy of doublet prediction in ArchR, we generated a scATAC-seq data set of 10 admixed cell lines (**Figure 1d-I, Extended Data Fig. 3a-h and 4a-d**). Doublets were identified with “addDoubletScores” and filtered with “filterDoublets”. To benchmark doublet calling in ArchR for scATAC-seq doublets we used Scrublet[22] (default parameters) on the 10x feature matrix (output from cell-ranger atac). ArchR’s doublet enrichment values and Scrublet’s doublet scores were then used to identify the Receiving Operating Characteristic (ROC, computed with the R package “PRROC”) and Precision Recall (PR, computed with the R package “PRROC”) area under the curves (AUC) respectively. To further benchmark ArchR’s doublet calling procedure, we utilized the scRNA-seq PBMC mixing data from Kang et al. 2017[23]. We identified doublet scores from Scrublet (n_prin_comps=30, mean_center=True, normalize_variance=True) and from ArchR, by using a custom function for computing doublet scores from a matrix (see publication page github, variableFeatures=3,000, binarize=FALSE, firstSelection=”variable”). We calculated the ROC and PR AUC’s respectively for both Scrublet doublet scores and ArchR doublet enrichment values. For the doublet score analysis related to the 10x Multiome PBMC data, please refer to the 10x Genomics Multiome methods section (see **ArchR Analysis – 10x Genomics Multiome PBMCs**).

ArchR Methods – Identification of Clusters

ArchR uses established scRNA-seq clustering methods that use graph clustering on the LSI dimensionality reduction coordinates to resolve clusters. By default, ArchR uses Seurat's graph clustering with "Seurat::FindClusters" for identifying high fidelity clusters[12]. ArchR additionally supports scran[51] for single-cell clustering. Lastly, ArchR can merge clusters to a desired number (user-defined) by using "hclust" and "cutree" on the distance between the average positions (of the initial clustering) in the LSI subspace.

ArchR Methods – t-SNE and UMAP Embeddings

ArchR supports both t-distributed stochastic neighbor embedding (t-SNE) and uniform manifold approximation and projection (UMAP) single-cell embedding methodologies. ArchR uses previously determined reduced dimensions as input for these embeddings. t-SNE analysis is performed using the "Rtsne" package in R by default. UMAP analysis is performed using the "uwot" package in R by default. The results are stored within an ArchRProject and then used for plotting and subsequent analyses.

ArchR Methods – Sample-Aware Pseudo-Bulk Replicate Generation

Because of the sparsity of scATAC-seq data, operations are often performed on aggregated groups of single cells. Most frequently, these groups are defined by clustering, and it is assumed that each local cluster represents a relatively homogeneous cell type or cell state. This process of combining data from multiple individual cells creates "pseudo-bulk" data, because it resembles the data derived from a bulk ATAC-seq experiment.

A feature unique to ArchR is the creation of sample-aware pseudo-bulk replicates from each cell group to use for performing statistical tests (such as reproducible peak identification or TF footprinting). ArchR does this via a complex decision tree which is dependent upon a user-specified desired number of replicates and number of cells per replicate as presented in **Supplementary Fig. 9a**. Briefly, ArchR attempts to create pseudo-bulk replicates in a sample-aware fashion. This means that each individual pseudo-bulk replicate only contains cells from a single biological sample. This feature enables the preservation of variability associated with biological replicates. If the desired number of replicates cannot be created in this fashion, ArchR uses progressively less stringent requirements to create the pseudo-bulk replicates. First, ArchR attempts to create as many pseudo-bulk replicates in a sample-aware fashion as possible and then create the remaining pseudo-bulk replicates in a sample-agnostic fashion by sampling without replacement. If this is not possible, ArchR attempts to create the desired number of pseudo-bulk replicates in a sample-agnostic fashion by sample without replacement across all samples. If this is not possible, ArchR attempts the same procedure by sampling without replacement within a single replicate but with replacement across different replicates without exceeding a user-specified sampling ratio. If all of these attempts fail, ArchR will create the specified number of pseudo-bulk replicates by sampling with replacement within a single replicate and with replacement across different replicates. The fragments from all cells within a pseudo-bulk replicate are converted to insertions and to a run-length encoding (RLE) coverage object using the "coverage" function in R. This insertion coverage object (similar to a bigwig) is then written to a separate HDF5-formatted coverage file. ArchR next identifies single-base resolution Tn5 insertion sites for each pseudo-bulk replicate, resizes these 1-bp sites to k-bp (user-defined, default is 6) windows (-k/2 and + (k/2 - 1) bp from insertion), and then creates a k-mer frequency table using the "oligonucleotidefrequency(w=k, simplify.as="collapse")" function from the Biostrings package. ArchR then calculates the expected k-mers genome-wide using the same function with the

BSGenome-associated genome file. These Tn5 k-mer values represent the Tn5 bias genome-wide and are then stored in the pseudo-bulk replicate HDF5 coverage file. This coverage file contains similar information to a bigwig file with Tn5 insertion bias but in a fast-access HDF5 format. This coverage file can be used for peak-calling and TF footprinting with Tn5 bias correction.

ArchR Methods – Peak Calling

In ArchR, peak calling is performed on the HDF5-format pseudo-bulk-derived coverage files described above. By default, ArchR calls peak summits with MACS2 using single-base insertion positions derived from the coverage files (written to a bed file with data.table) with user-specified values for MACS2 parameters including gsize, shift (default -75), and extsize (default 150) along with the "nomodel" and "nolambda" flags. These single-base peak summit locations are extended to a 501-bp width. We use 501-bp fixed-width peaks because they make downstream computation easier as peak length does not need to be normalized. Moreover, the vast majority of peaks in ATAC-seq are less than 501-bp wide. Using variable-width peaks also makes it difficult to merge peak calls from multiple samples without creating extremely large peaks that create confounding biases.

To create a merged non-overlapping fixed-width union peak set, ArchR implements an iterative overlap removal procedure that we introduced previously[34]. Briefly, peaks are first ranked by their significance, then the most significant peak is retained and any peak that directly overlaps with the most significant peak is removed from further analysis. This process is repeated with the remaining peaks until no more peaks exist. This procedure avoids daisy-chaining and still allows for use of fixed-width peaks. We use a normalized metric for determining the significance of peaks because the reported MACS2 significance is proportional to the sequencing depth. This process is outlined in **Supplementary Fig. 9b**. In ArchR, peaks that overlap with blacklist regions are excluded during peak calling.

ArchR Methods – Interactive Genome Browser

One challenge inherent to scATAC-seq data analysis is genome-track level visualizations of chromatin accessibility observed within groups of cells. Traditionally, track visualization requires grouping the scATAC-seq fragments, creating a genome coverage bigwig, and normalizing this track for quantitative visualization. Typically, end-users use a genome browser such as the WashU Epigenome Browser, the UCSC Genome Browser, or the IGV browser to visualize these sequencing tracks. This process involves using multiple software and any change to the cellular groups or addition of more samples requires re-generation of bigwig files etc., which can become time consuming. For this reason, ArchR has a Shiny-based interactive genome browser that can be launched with a simple line of code "ArchRBrowser(ArchRProj)". The data storage strategy implemented in Arrow files allows this interactive browser to dynamically change the cell groupings, resolution, and normalization, enabling real-time track-level visualizations. The ArchR Genome Browser also creates high-quality vectorized images in PDF format for publication or distribution. Additionally, the browser accepts user-supplied input files such as BED files or GenomicRanges to display features or genomic interaction files that define co-accessibility, peak-to-gene linkages, or loops from chromatin conformation data.

To facilitate this interactive browser, ArchR utilizes the same optimizations described above for creating a genome-wide TileMatrix to create a TileMatrix for the chosen resolution specified within the plotting window. Cells corresponding to the same group are summed per tile and the resulting group matrix represents the accessibility in tiles across the specified window.

This matrix can then be normalized by either the total number of reads in TSS/peak regions, the total number of cells, or the total number of unique nuclear fragments. By default, ArchR uses the reads in TSS regions, because this value is computed upon the creation of an Arrow file and is stable across analyses, unlike the peak regions. Because fragments in Arrow files are split per chromosome, the low memory cost and high speed of this process enables interactive visualization of hundreds of thousands of cells in seconds. Additionally, ArchR can plot tracks without the genome browser using the ArchRBrowserTrack function. ArchR also enables direct export of group normalized bigwig files using "export.bw" from Rtracklayer that can be directly used in conventional genome browsers.

ArchR Methods - Peak Matrix

Once a peak set has been created (see ArchR Methods – Peak Calling), a cell x peak matrix can readily be made with ArchR. For each Arrow file, ArchR reads in scATAC-seq fragments from each chromosome and then computes overlaps with the peaks from the same chromosome. A sparse matrix cell x peak matrix is created for these peaks. The matrix is then added to the corresponding Arrow file. This procedure is iterated across each chromosome.

ArchR Methods – Creation of Low-Overlapping Aggregates of Cells for Linkage Analysis

ArchR facilitates many integrative analyses that involve correlation of features. Performing these calculations with sparse single-cell data can lead to substantial noise in these correlative analyses. To circumvent this challenge, we adopted an approach introduced by Cicero[18] to create low-overlapping aggregates of single cells prior to these analyses. We filter aggregates with greater than 80% overlap with any other aggregate in order to reduce bias. To improve the speed of this approach, we developed an implementation of an optimized iterative overlap checking routine and a implementation of fast feature correlations in C++ using the "Rcpp" package. These optimized methods are used in ArchR for calculating peak co-accessibility, peak-to-gene linkage, and for other linkage analyses.

ArchR Methods – Peak Co-Accessibility

Co-accessibility analyses have been shown to be useful in downstream applications such as identifying groups of peaks that are all correlated forming "co-accessible networks"[18]. ArchR can rapidly compute peak co-accessibility from a peak matrix. These co-accessibility links can optionally be visualized using the ArchRBrowser. First, ArchR identifies 500+ low-overlapping cell aggregates (see Creation of Low-Overlapping Aggregates of Cells for Linkage Analysis). Second, for each chromosome (independently stored within an Arrow file), ArchR reads in the peak matrix and then creates the cell aggregate x peak matrix. ArchR next identifies all possible peak-to-peak combinations within a given window (by default 250 kb) and then computes the Pearson correlation of the log2-normalized cell aggregate x peak matrix. In this procedure, column sums across all chromosomes are used for depth normalization. ArchR iterates through all chromosomes and then combines the genome-wide results and stores them within the ArchRProject. These can be readily accessed for downstream applications. Additionally, ArchR enables users to lower the resolution of these interactions to better visualize the main interactors (keeping the highest correlation value observed in each window).

ArchR Methods – Motif Annotations

ArchR enables rapid, fine-grained motif analyses. To carry out these analyses, ArchR must first identify the locations of all motifs in peak regions. ArchR natively supports access to motif sets curated from chromVAR[16] and JASPAR[52] to be used for these motif analyses. Additionally, ArchR makes possible the usage of multiple motif databases independently. ArchR first identifies motifs in peak regions using the matchMotifs function from the "motifmatchr" package (https://greenleaflab.github.io/motifmatchr/) with output being the motif positions within peaks. ArchR then creates a boolean motif overlap sparse matrix for each motif-peak combination that can be used for downstream applications such as enrichment testing and chromVAR. The motif positions and motif overlap matrix are stored on disk as an RDS file for later access, which minimizes the total memory of the ArchRProject, freeing memory for other analyses.

ArchR Methods – Feature Annotations

ArchR allows for peak overlap analyses with defined feature sets. These feature sets could be ENCODE ChIP-seq/ATAC-seq peak sets or anything that can be specified as a GenomicRanges object. To facilitate this operation, we have curated a compendium of previously published ATAC-seq peak sets[32,34–36,39], ENCODE ChIP-seq peak sets, and other custom feature sets for end-users[53]. We believe these custom feature sets will help users better annotate and describe cell types identified with scATAC-seq. These feature sets are overlapped with the ArchRProject peak set and then stored as a boolean feature overlap sparse matrix for each feature-peak combination that can be used for downstream applications such as enrichment testing and chromVAR. This feature overlap matrix is then stored on disk as an RDS file for later access, which minimizes the total memory of the ArchRProject, freeing memory for other analyses.

ArchR Methods – Marker Peak Identification

ArchR allows for identification of features that are highly specific to a given group/cluster to elucidate cluster-specific biology. ArchR can identify these features for any of the matrices that are created with ArchR (stored in the Arrow files). ArchR identifies marker features while accounting for user-defined known biases that might confound the analysis (defaults are the TSS enrichment score and the number of unique nuclear fragments). For each group/cluster, ArchR identifies a set of background cells that match for the user-defined known biases and weights each equivalently using quantile normalization. Additionally, when selecting these bias-matched cells ArchR will match the distribution of the other user-defined groups. For example, if there were 4 equally represented clusters, ArchR will match the biases for a cluster to the remaining 3 clusters while selecting cells from the remaining 3 groups equally. By selecting a group of bias-matched cells, ArchR can directly minimize these confounding variables during differential testing rather than using modeling-based approaches. ArchR allows for binomial testing, Wilcoxon testing (via presto, https://github.com/immunogenomics/presto/), and two-sided t-testing for comparing the group to the bias-matched cells. These p-values are then adjusted for multiple hypothesis testing and organized across all group/clusters. This table of differential results can then be used to identify marker features based on user-defined log2(Fold Change) and FDR cutoffs.

ArchR Methods – chromVAR Deviations Matrix

ArchR facilitates chromVAR analysis to identify deviation of accessibility within peak annotations (i.e. motif overlaps) compared to a controlled background set of bias-matched peaks. A challenge in using the published version of the chromVAR software is that it requires the full cell x peak matrix to be loaded into memory in order to compute these deviations. This can lead to dramatic

increases in run time and memory usage for moderately sized datasets (~50,000 cells). To circumvent these limitations, ArchR implements the same chromVAR analysis workflow by analyzing sample sub-matrices independently (see **Extended Data Fig. 8e**). First, ArchR reads in the global accessibility per peak across all cells. Second, for each peak, ArchR identifies a set of background peaks that are matched by GC-content and accessibility. Third, ArchR uses this background set of peaks and global accessibility to compute bias-corrected deviations with chromVAR for each sample independently. This implementation requires data from only 5,000-10,000 cells to be loaded into memory at any given time, minimizing the memory requirements, enabling scalable analysis with chromVAR, and improving run-time performance.

ArchR Methods – Identification of Positive TF-Regulators

ATAC-seq allows for the unbiased identification of TFs that exhibit large changes in chromatin accessibility at sites containing their DNA binding motifs. However, families of TFs (for ex. GATA factors) share similar features in their binding motifs when looking in aggregate through position weight matrices (PWMs). This motif similarity makes it challenging to identify the specific TFs that might be driving observed changes in chromatin accessibility at their predicted binding sites. To circumvent this challenge, we have previously used gene expression to identify TFs whose gene expression is positively correlated to changes in the accessibility of their corresponding motif[34]. We term these TFs "positive regulators". However, this analysis relies on matched gene expression data which may not be readily available in all experiments. To overcome this dependency, ArchR can identify TFs whose inferred gene scores are correlated to their chromVAR TF deviation scores. To achieve this, ArchR correlates chromVAR deviation scores of TF motifs with gene activity scores of TF genes from the low-overlapping cell aggregates (see above). When using scRNA-seq integration with ArchR, gene expression of the TF can be used instead of inferred gene activity score.

ArchR Methods – TF Footprinting

ATAC-seq enables profiling of TF occupancy at base-pair resolution with TF footprinting. TF binding to DNA protects the protein-DNA binding site from transposition while the displacement or depletion of one or more adjacent nucleosomes creates increased DNA accessibility in the immediate flanking sequence. Collectively, these phenomena are referred to as the TF footprint. To accurately profile TF footprints, a large number of reads are required. Therefore, cells are grouped to create pseudo-bulk ATAC-seq profiles that can be then used for TF footprinting.

One major challenge with TF footprinting using ATAC-seq data is the insertion sequence bias of the Tn5 transposase[34,54,55] which can lead to misclassification of TF footprints. To account for Tn5 insertion bias ArchR identifies the k-mer (user-defined length, default length 6) sequences surrounding each Tn5 insertion site. To do this analysis, ArchR identifies single-base resolution Tn5 insertion sites for each pseudo-bulk (see above Sample-Aware Pseudo-Bulk Replicate Generation), resizes these 1-bp sites to k-bp windows (-k/2 and + (k/2 - 1) bp from insertion), and then creates a k-mer frequency table using the "oligonucleotidefrequency(w=k, simplify.as="collapse")" function from the Biostrings package. ArchR then calculates the expected k-mers genome-wide using the same function with the BSgenome-associated genome file. To calculate the insertion bias for a pseudo-bulk footprint, ArchR creates a k-mer frequency matrix that is represented as all possible k-mers across a window +/- N bp (user-defined, default 250 bp) from the motif center. Then, iterating over each motif site, ArchR fills in the positioned k-mers into the k-mer frequency matrix. This is then calculated for each motif position genome-

wide. Using the sample's k-mer frequency table, ArchR can then compute the expected Tn5 insertions by multiplying the k-mer position frequency table by the observed/expected Tn5 k-mer frequency. For default TF footprinting with ArchR, motif positions (stored in the ArchRProject) are extended +/- 250 bp centered at the motif binding site. The pseudo-bulk replicates (stored as a HDF5-format coverage files) are then read into R as a coverage run-length encoding. For each individual motif, ArchR iterates over the chromosomes, computing a "Views" object using "Views(coverage,positions)". ArchR uses an optimized C++ function to compute the sum per position in the Views object. This implementation enables fast and efficient footprinting (**Extended Data Fig. 8g-i**).

ArchR Methods – Bulk ATAC-seq LSI projection

ArchR allows for projection of bulk ATAC-seq data into a scATAC-seq subspace as previously described[7]. ArchR first takes as input a bulk ATAC-seq sample x peak matrix and then identifies which peaks overlap the features used in the scATAC-seq dimensionality reduction. If there is sufficient overlap, ArchR estimates a scATAC-seq pseudo-cell x feature matrix within the features identified to overlap. These pseudo-cells (N = 250) per sample are sampled to be at 0.5x, 1x, 1.5x and 2x the average accessibility of the cell x feature matrix used. This step prevents unwanted sampling depth bias for this bulk projection analysis. The pseudo-cell x feature matrix is then normalized with the term-frequency x inverse document frequency (TF-IDF) method, using the same inverse document frequency obtained during the scATAC-seq dimensionality reduction. This normalized pseudo-cell x feature matrix is then projected with singular value decomposition "t(TF_IDF) %*% SVD$u %*% diag(1/SVD$d)" where TF_IDF is the transformed matrix and SVD is the previous SVD run using irlba in R. This reduced pseudo-cell x dim matrix can then be input to "uwot::umap_transform" which uses the previous scATAC-seq UMAP embedding to project the pseudo-cells into this embedding.

ArchR Methods – Data Imputation with MAGIC

ArchR allows for using features such as gene scores and chromVAR deviation scores to assist in cluster annotation. However, features such as gene scores suffer from dropout noise in single-cell data. For scRNA-seq there have been many imputation methods developed to remedy this dropout noise. We have found that an effective method for imputation with scATAC-seq data is with Markov affinity-based graph imputation of cells (MAGIC)[56]. ArchR implements MAGIC for diffusing single-cell features across similar cells to smooth a single-cell matrix while simultaneously accounting for drop-out biases. MAGIC creates and stores a cell x cell diffusion matrix of weights that is then used to smooth the feature matrix with matrix multiplication. However, this diffusion matrix is dense and scales quadratically with the number of cells. To circumvent this limitation, ArchR creates equally sized blocks of cells (user-defined, default is 10,000) and then computes the partial diffusion matrix for these cells. These partial diffusion matrices are then combined to create a blocked diffusion matrix. This blocked diffusion matrix scales linearly in size leading to more memory efficiency but leads to lower resolution diffusion of data. To increase the resolution of this blocked diffusion matrix ArchR creates multiple replicates of the diffusion matrix to independently smooth the data matrix and then takes the average of the resulting smoothed matrices. ArchR additionally stores these blocked diffusion matrix replicates on-disk in HDF5-formatted files where each block is stored as its own group for direct access to specific parts of the matrix. ArchR's MAGIC implementation shifts the memory usage to on-disk storage and thus enables data diffusion of extremely large datasets (N > 200,000)

with minimal computing requirements. We note that MAGIC imputation is only used for improving interpretation of the data and is not used for dimensionality reduction, clustering or differential testing.

ArchR Methods – scATAC and scRNA Alignment

ArchR allows for efficient integration with scRNA-seq data utilizing Seurat's integration infrastructure[12]. When performing this cross-platform alignment across large numbers of cells, we have found that the required memory and run time increase substantially. Moreover, constraining this alignment into smaller biologically relevant parts minimizes the alignment space into smaller alignment "sub-spaces"[7]. Thus, to increase alignment accuracy and improve runtime performance, ArchR enables the alignment of scATAC-seq and scRNA-seq to be constrained by user-defined groups of cells from both datasets that define smaller alignment sub-spaces. Within these sub-spaces, ArchR splits the scATAC-seq cells into equivalent slices of N cells (user-defined, default is 10,000 cells) and performs alignment with the scRNA-seq cells. This alignment procedure begins with the identification of the top variable genes (user-defined, default is 2,000 genes defined from scRNA-seq) using "Seurat::FindVariableFeatures". Next, ArchR reads in the cell x gene scores matrix from the Arrow file for these cells. Then, ArchR imputes these gene scores using MAGIC and stores this imputed gene score matrix into a Seurat object for integration. ArchR then uses "Seurat::FindTransferAnchors" with CCA to align this sub-space of cells efficiently. Next, ArchR extracts the aligned scRNA-seq cell, group, and gene expression profile with "Seurat::TransferData". These gene expression profiles are stored in the corresponding Arrow files (stored as "GeneIntegrationMatrix") for downstream analyses.

ArchR Methods – scRNA Peak-To-Gene Linkage

We have previously used ATAC-seq peak-to-gene linkages to link putative enhancers and GWAS risk loci to their predicted target genes[7,34]. ArchR can rapidly compute peak-to-gene links from a peak matrix and gene expression matrix (see above). These peak-to-gene links can optionally be visualized using the ArchRBrowser. First, ArchR identifies 500+ low-overlapping cell aggregates (see Creation of Low-Overlapping Aggregates of Cells for Linkage Analysis). Second, ArchR reads in the peak matrix and then creates the cell aggregate x peak matrix. Third, ArchR reads in the gene expression matrix and then creates the cell aggregate x gene matrix. ArchR then identifies all possible peak-to-gene combinations within a given window of the gene start (user-defined, default is 250 kb) and then computes the Pearson correlation of the log2-normalized cell aggregate x peak matrix and cell aggregate x gene matrix across all cell aggregates. ArchR computes these peak-to-gene links genome-wide and stores them within the ArchRProject, which can then be accessed for downstream applications. Additionally, ArchR enables users to lower the resolution of these interactions to better visualize the main interactors (keeping only the highest correlation value observed in each window).

ArchR Methods – Cellular Trajectory Analysis

To order cells in pseudo-time, ArchR creates cellular trajectories that order cells across a lower N-dimensional subspace within an ArchRProject. Previously, we have performed this ordering in the 2-dimensional UMAP subspace[6] but ArchR has improved upon this methodology to enable alignment within an N-dimensional subspace (i.e. LSI). First, ArchR requires a user-defined trajectory backbone that provides a rough ordering of cell groups/clusters. For example, given user-determined cluster identities, one might provide the cluster IDs for a stem cell cluster, then a

progenitor cell cluster, and then a differentiated cell cluster that correspond to a known or presumed biologically relevant cellular trajectory (i.e. providing the cluster IDs for HSC, to MPP, to CMP, to Monocyte). Next, for each cluster, ArchR calculates the mean coordinates for each cell group/cluster in N-dimensions and retains cells whose Euclidean distance to those mean coordinates is in the top 5% of all cells. Next, ArchR computes the distance for each cell from $cluster_i$ to the mean coordinates of $cluster_{i+1}$ along the trajectory and computes a pseudo-time vector based on these distances for each iteration of i. This allows ArchR to determine an N-dimensional coordinate and a pseudo-time value for each of the cells retained as part of the trajectory based on the Euclidean distance to the cell group/cluster mean coordinates. Next, ArchR fits a continuous trajectory to each N-dimensional coordinate based on the pseudo-time value using the "smooth.spline" function with df = 250 (degrees of freedom) and spar = 1 (smoothing parameter). Then, ArchR aligns all cells to the trajectory based on their Euclidean distance to the nearest point along the manifold. ArchR then scales this alignment to 100 and stores this pseudo-time in the ArchRProject for downstream analyses. ArchR additionally, enables alternative trajectory analysis with both Slingshot[40] ("addSlingShotTrajectories") and Monocle3[41–43] ("getMonocleTrajectories" and "addMonocleTrajectory").

ArchR can create matrices that convey pseudo-time trends across features stored within the Arrow files. For example, ArchR can analyze changes in TF deviations, gene scores, or integrated gene expression across pseudo-time to identify regulators or regulatory elements that are dynamic throughout the cellular trajectory. First, ArchR groups cells in small user-defined quantile increments (default = 1/100) across the cellular trajectory. ArchR then smooths this matrix per feature using a user-defined smoothing window (default = 9/100) using the "data.table::frollmean" function. ArchR then returns this smoothed pseudo-time x feature matrix as a SummarizedExperiment for downstream analyses. ArchR additionally can correlate two of these smoothed pseudo-time x feature matrices using name matching (i.e. positive regulators with chromVAR TF deviations and gene score/integration profiles) or by genomic position overlap methods (i.e. peak-to-gene linkages) using low-overlapping cellular aggregates as described in previous sections. Thus, ArchR facilitates integrative analyses across cellular trajectories, revealing correlated regulatory dynamics across multi-modal data.

Assessment of features present in other scATAC-seq analysis software

When designing ArchR we identified a diverse list of important scATAC-seq software features. Many existing scATAC-seq software packages perform certain aspects of scATAC-seq analysis but we note that there are fewer packages that enable comprehensive analysis of scATAC-seq data from start to finish. We compiled a list of existing software based on Chen et al. 2019[10] and identified (to the best of our ability based on the software, version shown in **Extended Data Fig. 1a**, website, and associated analysis tutorials) features that were supported amongst our list. Chen et al. 2019[10] rigorously benchmarked most of these scATAC-seq software using smaller datasets (<10,000 cells) noting that only SnapATAC was capable of analyzing datasets of >80,000 cells, indicating that these other tools were not designed to handle the scale of current data generation efforts. We opted to quantitatively benchmark SnapATAC and Signac which we felt were objectively the 2 software with the most comparable feature sets. We note that we initially tested CisTopic in this quantitative benchmarking but found that analysis of a 10,000-cell PBMC dataset exceeded the memory limits in a computational environment with 8 cores and 32GB of RAM.

Benchmarking Analysis – Preface

For benchmarking analyses, we used one of two computational environments: (1) a MacBook Pro laptop containing 32 GB of RAM and a 2.3GHz 8-core Intel Core i9 processor (16 threads) with data stored on an external USB hard drive; (2) a large-memory node on a high-performance cluster with 128 GB of RAM and two 2.40 GHz 10-core Intel Xeon E5-2640 V4 processors (20 threads). For benchmarking analyses using more limited compute resources (32 GB and 8 cores) we used the same large-memory node configuration but limited the available cores and memories using Slurm job submission properties. The main difference between the computational environment of the MacBook Pro and the server is the ability of each core on the MacBook Pro to use 2 threads whereas hyper-threading is disabled on the server and each core is effectively a single thread.

We downloaded scATAC-seq data from previously published and publicly available locations. We downloaded the immune cell data fragment files from Satpathy et al. 2019 (GSE129785), Granja et al. 2019 (GSE139369), and from the 10x Genomics website (https://www.10xgenomics.com/solutions/single-cell-atac/). For the mouse sci-ATAC-seq data, we downloaded the BAM files from http://atlas.gs.washington.edu/mouse-atac/. No additional steps were used prior to benchmarking analysis. We chose to focus our benchmarking tests versus Signac and SnapATAC based on the performance of LSI and LDM shown previously[10]. We ran all analyses in triplicate using snakemake via a slurm job submission engine on a high-performance cluster to accurately limit the available memory and cores. In the case of job failure, we allowed for multiple job attempts to ensure that analyses were reproducible. After each failed job attempt, the number of parallel threads for each software was lowered to attempt to complete the analysis without exceeding the available memory. Unless otherwise stated all analyses were run with default parameters for scATAC-seq benchmarking. We provide R markdown html files on our publication page https://github.com/GreenleafLab/ArchR_2020 detailing the exact procedures used for all benchmarking analyses.

Benchmarking Analysis – Signac

Signac (version 0.2.2) (https://github.com/timoast/signac) requires a predetermined peak set, thus we downloaded the previously published bulk hematopoiesis peak set from Corces et al. (ftp://ftp.ncbi.nlm.nih.gov/geo/series/GSE74nnn/GSE74912/suppl/GSE74912_ATACseq_All_Counts.txt.gz) for all analyses. We first determined which cellular barcodes had more than 1,000 fragments by using "data.table::fread". For each individual sample, we created a cell x peak matrix with the "FeatureMatrix" function using the fragment files and abundant cell barcodes as input. Then, we created a Seurat object from this cell x peak matrix with "CreateSeuratObject". We then determined TSS enrichment scores for each cell across the first 3 chromosomes with the "TSSEnrichment" function. Default behavior for the TSSEnrichment function uses the first 2,000 TSSs; however, we increased this number (to include all TSSs on chr1-3) in order to stabilize the TSS enrichment scores for more consistent high-quality cell determination while still minimizing the run time. We then kept cells with a TSS enrichment score greater than 2 as high-quality cells passing filter. This TSS score cutoff differs from that of ArchR due to differences in the formula used for calculating TSS enrichment scores and differences in the gene annotation reference used by Signac. We then merged these individual Seurat objects (corresponding to each sample) and then performed TF-IDF normalization with "RunTFIDF" and "RunSVD" for LSI dimensionality reduction. We used the top 25% of features (ranked by accessibility) for LSI to reduce memory usage. The first 30 components were used by default for downstream analyses. Clusters were identified using "FindClusters" with default parameters. The scATAC-seq embeddings were determined using "RunUMAP" for UMAP and "RunTSNE" for tSNE respectively. Lastly, the

gene score matrix was created using "FeatureMatrix" on the gene start and end coordinates (provided from ArchR) extended upstream by 2 kb for each sample and combined afterwards followed by log-normalization.

Benchmarking Analysis - SnapATAC

SnapATAC (version 1.0.0) (https://github.com/r3fang/SnapATAC) requires additional preprocessing steps prior to creation of a Snap file that can be used for downstream analyses. First, fragment files were sorted by their cell barcode with Unix "sort". Next, these sorted fragment files were converted to Snap files by using SnapTools "snap-pre" with parameters "--min-mapq=30 --min-flen=50 --max-flen=1000 --keep-chrm=FALSE --keep-single=FALSE --keep-secondary=FALSE --overwrite=TRUE --min-cov=1000 --max-num=20000 --verbose=TRUE" as described on the GitHub page. A genome-wide tile/bin matrix was then added using "snap-add-bmat" with parameters "--bin-size-list 5000" (for 500 bp analysis this parameter was set to "--bin-size-list 500") for a 5-kb matrix. To identify high-quality cells, SnapATAC computes a promoter ratio score for the fraction of accessible fragments that overlap promoter regions. We read in the 5-kb bin matrix into a Snap object using "addBmatToSnap" and then created a promoter Genomic Ranges object from the provided transcript annotation file (http://renlab.sdsc.edu/r3fang/share/github/reference/hg19/gencode.v30.annotation.gtf.gz) and then extending the gene start upstream by 2 kb. Next, we overlapped these regions using "findOverlaps" and then computed the summed accessibility within these overlapping regions vs the total accessibility across all 5-kb bins. We chose a cutoff for promoter ratio as 0.175 by manually inspecting the benchmarking dataset total accessibility vs promoter ratio plot as described in the GitHub. These high-quality cells were kept for downstream analyses. For dimensionality reduction, we first filtered bins that were greater than the 95th percentile of non-zero bins. Next, we ran "runDiffusionMaps" with 30 eigenvectors to be computed (similar in the benchmarking analysis of all 3 methods). Clustering was performed with "runKNN" with the first 20 eigenvectors for a k-nn nearest neighbor search followed by "runCluster" with louvain.lib = "R-igraph". The scATAC-seq embeddings were determined using "runViz" with method = "umap" for UMAP and method = "Rtsne" for tSNE for the top 20 eigenvectors. Lastly, the gene score matrix was determined by using the gene start and end coordinates (provided from ArchR) as input to "createGmatFromMat" with the input.mat = "bmat" and scaled with "scaleCountMatrix". For comparing estimated dimensionality reduction in SnapATAC (estimated LDM) to estimated LSI in ArchR, we first sampled N cells (10,000 or the number of cells specified) based on the inverse of their coverage and then computed diffusion maps with "runDiffusionMaps". The remaining cells were projected with "runDiffusionMapsExtension" and the two Snap objects were combined for downstream analysis.

Benchmarking Analysis - ArchR

For analysis with ArchR, we first converted input scATAC-seq data (fragment files or BAM files) to Arrow files with "createArrowFiles" with minFrags = 1000, filterTSS = 4, and addGeneScoreMat = FALSE (addGeneScoreMat was set to false to allow for downstream benchmarking of this individual step). These Arrow files were then used to create an ArchRProject with the appropriate genome annotation. We identified doublet scores for each sample with "addDoubletScores" and "filterDoublets" respectively; however, time and memory used for doublet identification were not included in the benchmarking results because this step is unique to ArchR and would complicate direct comparisons to other software. We then computed the iterative

LSI dimensionality reduction with "addIterativeLSI" (For low-quality (downsampled ATAC-seq): variableFeatures = 100,000, resolution = 2, and maxClusters = 10; For medium and high-quality (downsampled ATAC-seq): variableFeatures = 25,000, resolution = 2, and maxClusters = 10; For scATAC-seq: variableFeatures = 25,000). Clusters were identified using "addClusters" with default parameters. The scATAC-seq embeddings were determined using "addUMAP" for UMAP and "addTSNE" for tSNE. Lastly, the gene score matrix was added by "addGeneScoreMatrix" which stores the depth-normalized cell x gene matrix. For comparison of estimated LSI in ArchR to estimated LDM in SnapATAC, "addIterativeLSI" was run with an additional parameter for sampling (sampleCellsFinal = 10,000 or the number of cells specified).

ArchR Analysis – Comparison of Gene Score Methods

We used ArchR to benchmark 53 models of inferring gene scores to emulate gene expression. All models were tested with the same gene annotation reference for direct comparison. We additionally used Signac, SnapATAC, and co-accessibility to create gene score models for comparison, making a total of 56 models. We used two datasets for evaluation: (1) ~30,000 PBMCs and (2) ~30,000 bone marrow cells. We first created the gene score models that incorporated distance by systematically changing the input parameters for "addGeneScoreMatrix". This parameter sweep included TSS exponential decay functions (useTSS = TRUE) and gene body exponential decay functions (useTSS = FALSE). We tried other decay functions but saw no appreciable difference so we used exponential decay (this is a user-input so any model as a function of relative distance may be inserted). For gene score models that were overlap-based (no distance function), we used "addFeatureMatrix" based on a set of genomic regions corresponding to either an extended gene promoter [resize(genes, 1, "start") followed by resize(2*window + 1, "center")] or an extended gene body [extendGR(genes, upstream, downstream)]. For each model, we created a genome-wide gene score matrix and extracted these matrices from the Arrow files using "getMatrixFromProject". We next created 500 low-overlapping random groupings of 100 cells with ArchR (see above) and took the average gene scores for each of these groupings. Next, we collected the gene scores calculated by Signac and SnapATAC during our benchmarking tests and averaged the gene scores across the same groupings. For co-accessibility, we created gene scores as previously described with Cicero[6,7,18]. We first used Cicero to create 5,000 lowly-overlapping cell groupings of 50 cells with "cicero_cds". Next, we calculated the average accessibility for these groupings across all peaks (with getMatrixFromProject). We correlated all peaks within 250 kb to get peak co-accessibility. We annotated the peaks as promoter if within 2.5 kb from the gene start with "annotate_cds_by_site". Finally, gene scores for the co-accessibility model were identified with "build_gene_activity_matrix" with a co-accessibility cutoff of 0.35 followed by "normalize_gene_activities". For this co-accessibility model, we tested various parameters such as promoter window size, correlation cutoff, and peak-to-peak distance maximums to make sure the results were reproducible.

Having a cell aggregate x gene score matrix for all 56 models, we next created a gene expression matrix to test these models. We integrated our scATAC-seq (from ArchR's results) with previously annotated scRNA-seq datasets (10k PBMC from 10x website and Bone Marrow from Granja et al., 2019) using "Seurat::FindTransferAnchors" and "Seurat::TransferData" with the top 2,000 variable genes from scRNA-seq. This integration was performed for each scATAC-seq sample independently and the scRNA-seq data used for each bone marrow alignment was constrained to match cell sources together (i.e. BMMC scATAC-seq with BMMC scRNA-seq and CD34+ scATAC-seq with CD34+ scRNA-seq)[7]. From this integration, each scATAC-seq cell was

paired to a matched gene expression profile. We averaged the gene expression profiles for each of the 500 lowly-overlapping groups (see above) to create a cell aggregate x gene expression matrix.

To benchmark the performance for each gene score model, we identified 2 gene sets: the top 2,000 variable genes defined by "Seurat::FindVariableGenes" and the top 1,000 differentially expressed genes defined by "Seurat::FindAllMarkers" (ranking the top N genes for each scRNA-seq cluster until 1,000 genes were identified). For these gene sets, we calculated the gene-wise correlation (how well do the gene score and gene expression correlate across all genes) and the aggregate-wise correlation (how well do the gene score and gene expression correlate across all cell aggregates). These 4 measures were then ranked across all models, and the average ranking was used to score the 56 models.

To orthogonally support this result, we downloaded previously published paired bulk ATAC-seq + RNA-seq for hematopoiesis[32]. We then iteratively down-sampled the reads from each dataset to create 100 pseudo-cells with 10,000 fragments from each bulk ATAC-seq sample. We then created a scATAC-seq fragments file for each pseudo-cell. We performed an identical analysis as described above for the 53 ArchR gene score models. For comparing these 53 models, we used 2 gene sets: the top 2,000 variable genes defined by log2-normalized expression-ranked variance across each cell type and the top 1,000 marker genes defined by the top log2(fold change) for each cell type vs the average expression of all cell types. We similarly ranked the gene-wise and aggregate-wise correlation across all models, and used the average ranking to score each model.

ArchR Analysis – 10x Genomics Multiome PBMCs

We used ArchR to analyze a PBMC dataset generated using the new 10x Genomics Multiome kit (paired scATAC-seq and scRNA-seq). First, we converted the fragments file to an Arrow file using "createArrowFiles" with valid barcodes (using "getValidBarcodes") from the 10x summary single cell file. Second, we imported the 10x gene expression matrix using "import10xFeatureMatrix" on the "feature_bc_matrix.h5" file. We next added this matrix to the Arrow file using "addGeneExpressionMatrix". We created an ArchRProject and then filtered cells that had paired gene expression, a TSS enrichment score greater than 6 and more than 2,500 unique fragments. Following filtration, a new ArchRProject was created following doublet removal using "addDoubletScores" and "filterDoublets". For both ArchRProjects (with and without doublets), we computed an iterative LSI reduction for both the scATAC-seq tile matrix (variableFeatures = 25,000) and scRNA-seq gene expression matrix (variableFeatures=2,500, firstSelection = "variable", binarize = FALSE). To combine these reductions, we used "addCombinedDims" equally weighting the scATAC-seq and scRNA-seq LSI reductions. UMAPs for each individual and the combined LSI reductions were created with "addUMAP" (minDist = 0.8). Clusters were determined using "addClusters" (resolution = 0.4).

We additionally, utilized this paired dataset to benchmark the accuracy of doublet calling and gene score models. To evaluate doublet calling for scATAC-seq we first identified Scrublet scores (n_prin_comps=30, mean_center=True, normalize_variance=True) for each cell in the scRNA-seq space, using genes that passed filter as described above (see methods - **ArchR Methods – Paired scRNA-seq Gene Expression Matrix**) by exporting the matrix with "getMatrixFromProject" and saving this matrix with "writeMM". We next accessed the doublet enrichment values obtained from ArchR and created a "data.frame" containing the two doublet metrics for each cell. We determined the Receiving Operating Characteristic (ROC, computed with the R package "PRROC") and Precision Recall (PR, computed with the R package "PRROC")

area under the curves (AUC) respectively for (1) ArchR's doublet enrichment values vs the top 750 cells identified from Scrublet as true doublets and (2) Scrublet's score vs the top 750 cells identified from ArchR as true doublets. To evaluate the gene score models in ArchR with this paired scATAC-seq and scRNA-seq dataset we used the combined LSI dimensionality reduction (without doublets) and computed ~500 non-overlapping groups of 100 cells. We averaged the gene expression matrix over those groups of cells and saved this matrix and the cell groupings for comparing gene score models. We evaluated the gene score models as described above (see methods - **ArchR Analysis – Comparison of Gene Score Methods**) with an additional 4 models for "TSS Extended Exponential + Gene Boundary".

ArchR Analysis – Large Simulated PBMC ~1.2M Cells

To further test ArchR's capability to analyze extremely large datasets (N > 200,000), we simulated ~1.3M single cells contained within 200 fragment files. We used 4 PBMC samples (2 x 5,000 cells and 2 x 10,000 cells from 10x Genomics) for creating this large dataset. We randomly shifted each scATAC-seq fragment with a mean difference of +/- 50-100 bp (randomly sampled) and a standard deviation of +/- 10-20 bp (randomly sampled). We then sampled the fragments by 80% to ensure some differences between simulated cells and then saved these to bg-zipped fragment files. We then used ArchR to convert these fragment files to Arrow files with "createArrowFiles" with minFrags = 1000, filterTSS = 4 and addGeneScoreMat = TRUE. These Arrow files were then assembled into an ArchRProject. We identified doublet scores for each simulated dataset with "addDoubletScores" and "filterDoublets" respectively, retaining ~1.2 million cells after doublet removal. We then computed the estimated iterative LSI dimensionality reduction with "addIterativeLSI" (variableFeatures = 25,000, sampleCellsFinal = 25,000 and 2 iterations). Estimated clusters were identified using "addClusters" with sampleCells = 50,000. This estimation method uses a subset of cells to cluster and then the remaining cells are annotated by their nearest neighbors (the maximum annotation observed). An estimated scATAC-seq UMAP was created using "addUMAP" with sampleCells = 100,000. This estimation method uses a subset of cells to create a UMAP embedding and then the remaining cells are projected into the single-cell embedding using "umap::umap_transform".

We additionally used these fragment files to test ArchR's performance with variable numbers of cells from 10,000 to 1,000,000 cells for both 8 cores 32GB and 20 cores 128GB computational set ups. We randomly created three replicates for each desired cell number size and performed a similar analysis as above. The estimated iterative LSI dimensionality reduction was only used when cell number exceeded 25,000.

ArchR Analysis – Large Hematopoiesis 220K Cells

We wanted to test ArchR's full analysis suite with a large dataset (N > 200,000) comprised of previously published immune cell data[6,7]. We additionally grouped all Fluidigm C1-based scATAC-seq data from Buenrostro et al. 2018[4] into a fragment file. This amounted to a total of 49 scATAC-seq fragment files corresponding to over 200,000 cells. We first used ArchR to convert these fragment files to Arrow files using "createArrowFiles" with minFrags = 1000, filterTSS = 8 and addGeneScoreMat = TRUE. These Arrow files are then used to create an ArchRProject. We identified doublet scores for each simulated dataset with "addDoubletScores" and "filterDoublets" respectively. We then computed the estimated iterative LSI dimensionality reduction with "addIterativeLSI" (variableFeatures = 25,000, sampleCellsFinal = 25,000 and iterations = 2). A scATAC-seq UMAP was then created by using "addUMAP" with minDist = 1 and nNeighbors =

40. Clusters were initially identified using "addClusters" with default parameters. We re-clustered the early progenitor cells (clusters containing CD34+ cells) with a clustering resolution of 0.4 to better resolve these cell clusters. We added MAGIC imputation weights with "addImputationWeights" for imputing single-cell features that are then overlaid on the UMAP embedding. We then manually merged and assigned clusters that correspond to cell types based on known marker gene scores (as shown in **Extended Data Fig. 8d**), observation of sequencing tracks using the ArchRBrowser (as shown in **Figure 3i and Supplementary Fig. 10**), overlap enrichment with hematopoietic cell type specific peaks (**Supplementary Fig. 9d**), visualization of sorted scATAC-seq cells within the data set (**Extended Data Fig. 8c**), and integration with previously published scRNA-seq (**Figure 4b and Extended Data Fig. 9a**).

To identify a union peak set, we created group coverage files, which contain the aggregated accessibility of groups of single cells within a cluster, with "addGroupCoverages". We then created a reproducible peak set with "addReproduciblePeakSet" and a cell x peak matrix with "addPeakMatrix". Next, we determined background peaks that are matched in GC-content and accessibility with "addBgdPeak". For downstream motif-based analyses we added motif overlap annotations with "addMotifAnnotations" for CIS-BP version 1 motifs (version = 1). We computed a ChromVAR deviations matrix with "addDeviationsMatrix". We next identified positive TF regulators with "correlateMatrices" where useMatrix1 = "MotifMatrix" and useMatrix2 = "GeneScoreMatrix". To identify which of these correlated TF regulators had strong differential motif activity differences we calculated the average motif deviation scores with "exportGroupSE" for each cluster and computed the max observed deviation difference between any two clusters. This motif difference and the TF-to-gene score correlation were then used to identify positive regulators (correlation > 0.5 and a maximum deviation score difference > 50th percentile). Differential accessibility for each cluster was determined using "markerFeatures" with maxCells = 1000 and useMatrix = "PeakMatrix". Marker peaks were defined as peaks with a log2(Fold Change) > 1.5 and an FDR < 0.01 (Wilcoxon-test with presto, https://github.com/immunogenomics/presto/). We then determined enriched motifs with "peakAnnoEnrichment" in these marker peaks and plotted the motif enrichment p-values for the positive TF regulators. We then used previously published bulk ATAC-seq datasets from hematopoietic cells[32,35] and computed a sample by peak matrix (counting using "findOverlaps" after reading in fragments from the corresponding bam files) using the scATAC-seq peak set. We normalized this sample by peak matrix by converting the values to counts per million (CPM) and log2 transformation. For each cell type, we computed the average accessibility and then subtracted the average of the other cell types from average accessibility for that cell type. We then ranked the top 7,500 peaks for each cell type. To better distinguish T cell subtypes, we performed this same analysis with only the T cell subtypes and added an additional 2,500 cell type-specific peaks for each T cell subtype. We then used these peaks as input to add "addPeakAnnotations" and computed overlap enrichment with "peakAnnoEnrichment". TF footprints, with Tn5-bias correction, were calculated by "plotFootprints" with motif positions from "getPositions" and normMethod = subtract. Bulk hematopoietic ATAC-seq (GSE74912) was projected into the scATAC-seq subspace using "projectBulkATAC" with N = 250 cells. Peak co-accessibility was computed with "addCoAccessibility" and accessibility tracks were created with the ArchRBrowser.

We next wanted to integrate our scATAC-seq data with previously published hematopoietic scRNA-seq data[7]. To do this analysis, we used "addGeneIntegrationMatrix" with sampleCellsATAC = 10,000, sampleCellsRNA = 10,000, and a groupList specifying to group cells from T/NK clusters and cells from non-T/NK clusters for both scATAC-seq and scRNA-seq prior

to alignment. This constrained integration improved the alignment accuracy and added a matched gene expression profile for each scATAC-seq cell. We overlaid these gene expression profiles on the UMAP embedding with “plotEmbedding”. After this integration analysis, we identified peak-to-gene links with “addPeak2GeneLinks” and visualized them with “peak2GeneHeatmap”.

To create a cellular trajectory across B cell differentiation, we used “addTrajectory” with preFilterQuantile = 0.8, useAll = FALSE, and an initial trajectory of “HSC -> CMP.LMPP -> CLP.1 -> CLP.2 -> PreB -> B”. We next created trajectory matrices for “MotifMatrix”, “GeneScoreMatrix”, “GeneIntegrationMatrix” and “PeakMatrix”. We correlated the deviation score and gene score trajectory matrices with “correlateTrajectories”. Additionally, we correlated the deviation score and gene expression trajectory matrices with “correlateTrajectories”. We kept TFs whose correlation was 0.5 or greater for both of the correlation analyses. We determined these TFs as positive TF regulators across the B cell trajectory. We also used ArchR to identify peak-to-gene links across the B cell trajectory with “correlateTrajectories” with useRanges = TRUE, varCutOff1 = 0.9, and varCutOff2 = 0.9. Lastly, we grouped cells into 5 groups of cells based on pseudo-time across the B cell trajectory for track visualization (with the ArchRBrowser) and TF footprinting of the TF regulators.

SUPPLEMENTARY REFERENCES

46. Huber, W. *et al.* Orchestrating high-throughput genomic analysis with Bioconductor. *Nat. Methods* **12,** 115–121 (2015).
47. Adey, A. *et al.* Rapid, low-input, low-bias construction of shotgun fragment libraries by high-density in vitro transposition. *Genome Biol.* **11,** R119 (2010).
48. Buenrostro, J. D., Giresi, P. G., Zaba, L. C., Chang, H. Y. & Greenleaf, W. J. Transposition of native chromatin for fast and sensitive epigenomic profiling of open chromatin, DNA-binding proteins and nucleosome position. *Nat. Methods* **10,** 1213–1218 (2013).
49. Amemiya, H. M., Kundaje, A. & Boyle, A. P. The ENCODE blacklist: identification of problematic regions of the genome. *Sci. Rep.* **9,** 9354 (2019).
50. Korsunsky, I. *et al.* Fast, sensitive and accurate integration of single-cell data with Harmony. *Nat. Methods* **16,** 1289–1296 (2019).
51. Lun, A. T. L., McCarthy, D. J. & Marioni, J. C. A step-by-step workflow for low-level analysis of single-cell RNA-seq data with Bioconductor. [version 2; peer review: 3 approved, 2 approved with reservations]. *F1000Res.* **5,** 2122 (2016).
52. Fornes, O. *et al.* JASPAR 2020: update of the open-access database of transcription factor binding profiles. *Nucleic Acids Res.* **48,** D87–D92 (2020).
53. Sheffield, N. C. & Bock, C. LOLA: enrichment analysis for genomic region sets and regulatory elements in R and Bioconductor. *Bioinformatics* **32,** 587–589 (2016).
54. He, H. H. *et al.* Refined DNase-seq protocol and data analysis reveals intrinsic bias in transcription factor footprint identification. *Nat. Methods* **11,** 73–78 (2014).
55. Baek, S., Goldstein, I. & Hager, G. L. Bivariate genomic footprinting detects changes in transcription factor activity. *Cell Rep.* **19,** 1710–1722 (2017).
56. van Dijk, D. *et al.* Recovering Gene Interactions from Single-Cell Data Using Data Diffusion. *Cell* **174,** 716–729.e27 (2018).
