# Supplementary material for: ArchR is a scalable software package for integrative single-cell chromatin accessibility analysis
Source: Nat Genet. 2021 Feb 25;53(3):403–11. doi: 10.1038/s41588-021-00790-6 (PMC8012210; doi:10.1038/s41588-021-00790-6)
Supplement: Supplementary file 2 — Reporting Summary [file 41588_2021_790_MOESM2_ESM.pdf]

nature research

Corresponding author(s): Jeffrey M. Granja, Howard Y. Chang, William J. Greenleaf

Last updated by author(s): 12/31/2020

Reporting Summary

Nature Research wishes to improve the reproducibility of the work that we publish. This form provides structure for consistency and transparency in reporting. For further information on Nature Research policies, see Authors & Referees and the Editorial Policy Checklist.

Statistics

For all statistical analyses, confirm that the following items are present in the figure legend, table legend, main text, or Methods section.

n/a	Confirmed	
☐	☒	The exact sample size (n) for each experimental group/condition, given as a discrete number and unit of measurement
☐	☒	A statement on whether measurements were taken from distinct samples or whether the same sample was measured repeatedly
☒	☐	The statistical test(s) used AND whether they are one- or two-sided<br>*Only common tests should be described solely by name; describe more complex techniques in the Methods section.*
☒	☐	A description of all covariates tested
☐	☒	A description of any assumptions or corrections, such as tests of normality and adjustment for multiple comparisons
☐	☒	A full description of the statistical parameters including central tendency (e.g. means) or other basic estimates (e.g. regression coefficient) AND variation (e.g. standard deviation) or associated estimates of uncertainty (e.g. confidence intervals)
☒	☐	For null hypothesis testing, the test statistic (e.g. F, t, r) with confidence intervals, effect sizes, degrees of freedom and P value noted<br>*Give P values as exact values whenever suitable.*
☒	☐	For Bayesian analysis, information on the choice of priors and Markov chain Monte Carlo settings
☒	☐	For hierarchical and complex designs, identification of the appropriate level for tests and full reporting of outcomes
☐	☒	Estimates of effect sizes (e.g. Cohen's d, Pearson's r), indicating how they were calculated

Our web collection on statistics for biologists contains articles on many of the points above.

Software and code

Policy information about availability of computer code

Data collection	Cell Ranger 1.0.0 – Barcode Identification, Alignment, Filter, Deduplication
Data analysis	Code Availability and Documentation<br>Extensive documentation and a full user manual are available at www.ArchRProject.com. The software is open-source and all code can be found on GitHub at https://github.com/GreenleafLab/ArchR. Additionally, code for producing the majority of analyses from this paper is available at the publication page https://github.com/GreenleafLab/ArchR_2020.<br><br>Software Package and Associated Packages Versions<br>macs2 2.1.1.20160309 – Peak Calling<br>R version 3.6.1 – R environment for all custom code<br>ArchR - 0.2.1 - Software for analysis of scATAC-seq data.<br>rhdf5 - 2.30.1 - Software for HDF5 formatted analysis.<br>Irlba 2.3.3 – Running PCA/SVD on large matrices.<br>Rcpp 1.0.4 – Used for writing helpful C++ code to speed up operations.<br>Rtsne 0.15 – Used for t-SNE embeddings.<br>matrixStats 0.56.0 – Used for mathematical operations on large matrices.<br>cicero 1.4.2 – Used for calculating gene activity scores with Co-Accessibility.<br>chromVAR_1.8.0 – Calculating TF deviation scores which can be associated with TF activity.<br>Summarized Experiment 1.16.1 – R Data Class Environment used throughout analyses.<br>Motifmatchr 1.8.0 – Matching TF Motifs within peak regions<br>Seurat_3.1.2 – SNN Graph Clustering Implementation<br>GenomicRanges 1.38.0 - Genomic Ranges Operations used for overlap analyses<br>Matrix 1.2-14 – Sparse Matrix math implementations.<br>BSgenome 1.54.0 – Toolkit used for getting Genomic DNA sequences for motif matching and footprinting.

Rsamtools 2.2.3 – For manipulating BAM files within R.
uwot-0.1.5 - For creating UMAPs in R.

For manuscripts utilizing custom algorithms or software that are central to the research but not yet described in published literature, software must be made available to editors/reviewers. We strongly encourage code deposition in a community repository (e.g. GitHub). See the Nature Research guidelines for submitting code & software for further information.

Data

Policy information about availability of data

All manuscripts must include a data availability statement. This statement should provide the following information, where applicable:

- Accession codes, unique identifiers, or web links for publicly available datasets
- A list of figures that have associated raw data
- A description of any restrictions on data availability

Data Availability
Bulk and scATAC-seq data from the cell line mixing experiment are available through GEO accession number GSE162690. All other scATAC-seq data used were from publicly available sources as outlined in Supplementary Table 1. We additionally have made available other analysis files on our publication page https://github.com/GreenleafLab/ArchR_2020.

Field-specific reporting

Please select the one below that is the best fit for your research. If you are not sure, read the appropriate sections before making your selection.

☒ Life sciences ☐ Behavioural & social sciences ☐ Ecological, evolutionary & environmental sciences

For a reference copy of the document with all sections, see nature.com/documents/nr-reporting-summary-flat.pdf

Life sciences study design

All studies must disclose on these points even when the disclosure is negative.

Sample size	Sample size was set to make sure results were consistently reproducible. For computational benchmarking, we performed each analysis in triplicate. When possible, we included multiple replicates of scATAC-seq data sets to ensure fidelity in the analysis.
Data exclusions	No data were excluded from the manuscript.
Replication	All computational results presented in manuscript were reliably reproduced in triplicate. When possible, we included multiple replicates of scATAC-seq data sets to ensure fidelity in the analysis.
Randomization	No randomization was used because analyses were performed mostly on previously published data sets.
Blinding	No blinding was used because analyses were performed mostly on previously published data sets.

Reporting for specific materials, systems and methods

We require information from authors about some types of materials, experimental systems and methods used in many studies. Here, indicate whether each material, system or method listed is relevant to your study. If you are not sure if a list item applies to your research, read the appropriate section before selecting a response.

Materials & experimental systems

n/a	Involved in the study	
☒	☐	Antibodies
☐	☒	Eukaryotic cell lines
☒	☐	Palaeontology
☒	☐	Animals and other organisms
☒	☐	Human research participants
☒	☐	Clinical data

Methods

n/a	Involved in the study	
☒	☐	ChIP-seq
☒	☐	Flow cytometry
☒	☐	MRI-based neuroimaging

Eukaryotic cell lines

Policy information about cell lines

Cell line source(s)	Jurkat, THP1, K562, HeLa, HEK-293T, HT1080, T24, MCF7, MCF10A from ATCC; GM12878 from Coriell
Authentication	Cell lines were obtained directly from the listed provider and used shortly thereafter.

Mycoplasma contamination	All cell lines tested negative for mycoplasma contamination prior to use in experiments.
Commonly misidentified lines (See ICLAC register)	None of the cell lines used in this study are listed in this database.
